# CLCC1 promotes hepatic neutral lipid flux and nuclear pore complex assembly

**DOI:** 10.1038/s41586-025-10064-4

**Published:** 2026-02-25

**Authors:** Alyssa J. Mathiowetz, Emily S. Meymand, Güneş Parlakgül, Niek van Hilten, Emily F. Torres, Leonardo L. Artico, Kirandeep K. Deol, Mike Lange, Stephany P. Pang, Cody E. Doubravsky, Melissa A. Roberts, Danielle M. Jorgens, Reena Zalpuri, Misun Kang, Casadora Boone, Brian W. Parks, Yaohuan Zhang, David W. Morgens, Emily Tso Newman, Yingjiang Zhou, Saswata Talukdar, Michael Grabe, Gregory Ku, Tim P. Levine, Ana Paula Arruda, James A. Olzmann

**Affiliations:** 1 Department of Molecular and Cell Biology, University of California, Berkeley, Berkeley, CA USA; 2 Department of Metabolic Biology and Nutrition, University of California, Berkeley, Berkeley, CA USA; 3 Cardiovascular Research Institute, University of California, San Francisco, San Francisco, CA USA; 4 Department of Pharmaceutical Chemistry, University of California, San Francisco, San Francisco, CA USA; 5 Electron Microscope Laboratory, University of California, Berkeley, Berkeley, CA USA; 6 Department of Nutritional Sciences, University of Wisconsin-Madison, Madison, WI USA; 7 Department of Medicine, Division of Endocrinology, University of California, San Francisco, San Francisco, CA USA; 8 Merck & Co., South San Francisco, CA USA; 9 Diabetes Center, University of California, San Francisco, San Francisco, CA USA; 10 University College London Institute of Ophthalmology, London, UK; 11 Biohub, San Francisco, CA USA

**Keywords:** Organelles, Metabolism

## Abstract

Imbalances in lipid storage and secretion lead to hepatic steatosis, the accumulation of lipid droplets in hepatocytes^1,2^. Our understanding of the mechanisms that govern the channelling of neutral lipids in hepatocytes towards cytosolic lipid droplets or secreted lipoproteins remains incomplete^3,4^. Here we performed a series of CRISPR–Cas9 screens under different metabolic states that led to the identification of CLCC1 as a critical regulator of neutral lipid storage and secretion in hepatocytes. Loss of CLCC1 resulted in the buildup of large lipid droplets in hepatoma cells and *Clcc1* knockout in mice caused liver steatosis. Lipid droplets were present in the lumen of the endoplasmic reticulum of the *Clcc1*-knockout hepatocytes and exhibited properties of lipoproteins, indicating a profound shift in neutral lipid flux. The loss of CLCC1 also led to the accumulation of nuclear membrane herniations accompanied by a reduction in nuclear pores. Remote homology searches identified a domain in CLCC1 that is homologous to yeast Brl1 and Brr6, factors that promote nuclear envelope fusion during nuclear pore complex assembly. Molecular dynamics simulations and mutagenesis studies support a model in which CLCC1 mediates membrane bending and fusion. We propose that CLCC1 mediates membrane fusion to promote hepatic neutral lipid flux and nuclear pore complex assembly.

## Abstract (web summary)

CRISPR–Cas9 screening identifies CLCC1 as a factor that increases neutral lipid flux to prevent hepatic steatosis and promotes nuclear pore complex assembly by promoting membrane bending and fusion.

## Main

Lipid droplets (LDs) are the primary lipid storage organelle in cells^3,4^, providing a safeguard against lipotoxicity and acting as a lipid repository that can be broken down rapidly to release lipids for cellular utilization^3,4^. LDs are derived from the endoplasmic reticulum (ER) and consist of a core of neutral lipids, such as triacylglycerol (TAG), which is encircled by a phospholipid monolayer decorated with regulatory proteins^3,4^. In liver hepatocytes, TAG may either be stored in cytoplasmic LDs or packaged into very low density lipoproteins (VLDLs) in the ER lumen for secretion^1,5,6^. The mechanisms that govern the storage and secretion of neutral lipids in hepatocyte LDs remain incompletely understood, and addressing this gap in knowledge is crucial for the development of new therapeutic strategies targeting hepatic steatosis^1,2^.

## Genetic modifiers of lipid storage

**Fig. 1:**
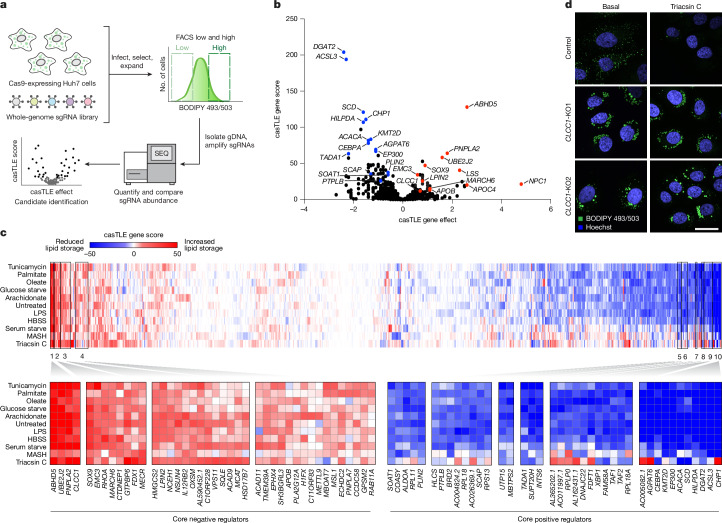
Parallel CRISPR–Cas9 screens reveal metabolic state-dependent genetic modifiers and identify CLCC1 as a key regulator of lipid storage. **a**, Schematic of FACS-based CRISPR–Cas9 screen approach to identify genes that regulate neutral lipid abundance, using BODIPY 493/503 as a neutral lipid reporter. gDNA, genomic DNA; SEQ, sequencing. **b**, Volcano plot indicating the gene effects (phenotype) and gene scores (confidence) for individual genes from batch retest screens in Huh7 cells. Gene effects and scores are calculated from two biological replicates. Genes of interest that increase (red) and decrease (blue) the amount of neutral lipid when deleted are highlighted. **c**, Heat map of clustered genes based on gene score across all conditions. Boxes 1–4 indicate clusters of core negative regulators that act to decrease LDs and boxes 5–10 indicate clusters of core positive regulators that act to increase LDs. HBSS, Hanks’ balanced salt solution; LPS, lipopolysaccharide; MASH, metabolic dysfunction-associated steatohepatitis. **d**, Representative confocal images of LDs labelled with BODIPY 493/503 in control (expressing safe-targeting sgRNA) and *CLCC1*-KO cells under basal conditions or following treatment with 1 µg ml^−1^ triacsin C for 24 h. Scale bar, 50 μm.

**Extended Data Fig. 1:**
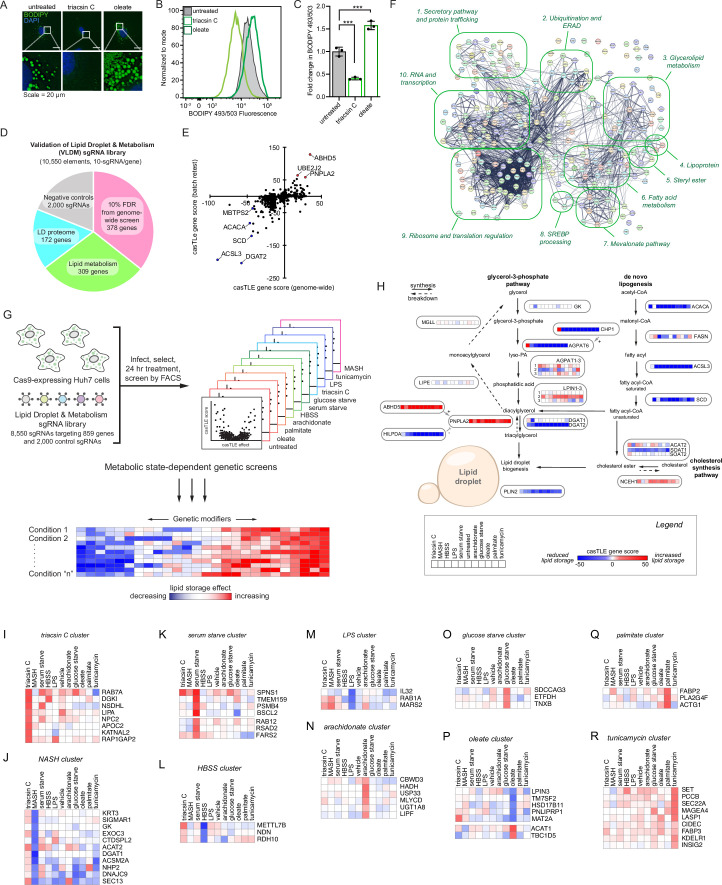
Analysis of lipid storage CRISPR-Cas9 screens and enriched pathways. A) Screen conditions were optimized in Huh7 cells treated for 24 h with 1 µg/mL triacsin C or 100 µM oleate and analyzed by fluorescence microscopy of cells labeled with BODIPY 493/503 (LDs) and DAPI (nuclei). B) Representative flow cytometry histograms of BODIPY 493/503 fluorescence in Huh7 cells untreated, treated with 1 µg/mL triacsin C, or treated with 100 µM oleate. C) Quantification of the amount of neutral lipid from flow cytometry histograms (representative histograms shown panel B) Data represent mean ± SD across three biological replicates. ****p < *0.0001 by one-way ANOVA with Dunnett’s multiple comparisons test*. D) Breakdown of the custom lipid metabolism CRISPR-Cas9 library used in batch retest screens. E) Pairwise comparison of gene scores between the genome-wide and batch retest screens. Scores were adjusted as positive or negative based upon the direction of gene effect. F) 265 of the 285 credible hits mapped in STRING confidence (text mining, experiments, physical interactions, genetic interactions, functional pathways) grouped manually by GO functional annotations. G) Schematic of parallel CRISPR screens under eleven different metabolic stress conditions. H) Schematic of LD synthesis and breakdown pathways. Genes are annotated with heatmaps corresponding to the gene score across metabolic conditions. Metabolic states follow the order indicated in the legend. I-R) Selected examples of gene clusters exhibiting metabolic state specific effects on neutral lipid storage. Source data

To identify genes involved in lipid storage, we performed a genome-wide fluorescence-activated cell sorting (FACS)-based CRISPR–Cas9 screen in Huh7 hepatoma cells using BODIPY 493/503 fluorescence as a reporter of neutral lipid storage (Fig. 1a, Extended Data Fig. 1a–c and Supplementary Table 1). Duplicate follow-up screens were performed using a custom validation of LD and metabolism (VLDM) single guide RNA (sgRNA) library to increase confidence and reduce false positives and negatives (Fig. 1b and Extended Data Fig. 1d,e). Genetic modifiers were associated with glycerolipid metabolism, including neutral lipid synthesis, lipolysis and additional processes that influence lipid metabolism (Fig. 1b, Extended Data Fig. 1f and Supplementary Fig. 1).

To provide insights into the genetic modifiers of lipid storage under different metabolic states, we performed duplicate FACS-based screens using our VLDM sgRNA library under 11 different metabolic conditions (Extended Data Fig. 1g and Supplementary Table 2). This series of 22 genetic screens provides chemical–genetic interaction data, enabling the clustering of genes with similar functional profiles and facilitating functional predictions for candidate regulators (Fig. 1c and Extended Data Fig. 1h–r). These findings highlight the importance of the glycerolipid metabolic pathways and identify differences in the utilization of members of enzyme families, such as the use of different 1-acylglycerol-3-phosphate O-acyltransferase (AGPAT), lipin and diacylglycerol acyltransferase (DGAT) enzymes under different conditions (Extended Data Fig. 1h–r and Supplementary Table 1). Genes that are known to have similar roles clustered together, such as the genes encoding the LD nucleation factor BSCL2 (also known as seipin) and its binding partner TMEM159 (also known as LDAF1 or promethin)^7^ (Extended Data Fig. 1k). These data establish a phenotype-rich compendium of genetic modifiers of neutral lipid storage. Screen data were deposited in the CRISPRlipid online data commons^8^ (https://crisprlipid.org/).

## CLCC1 controls LD dynamics

**Extended Data Fig. 2:**
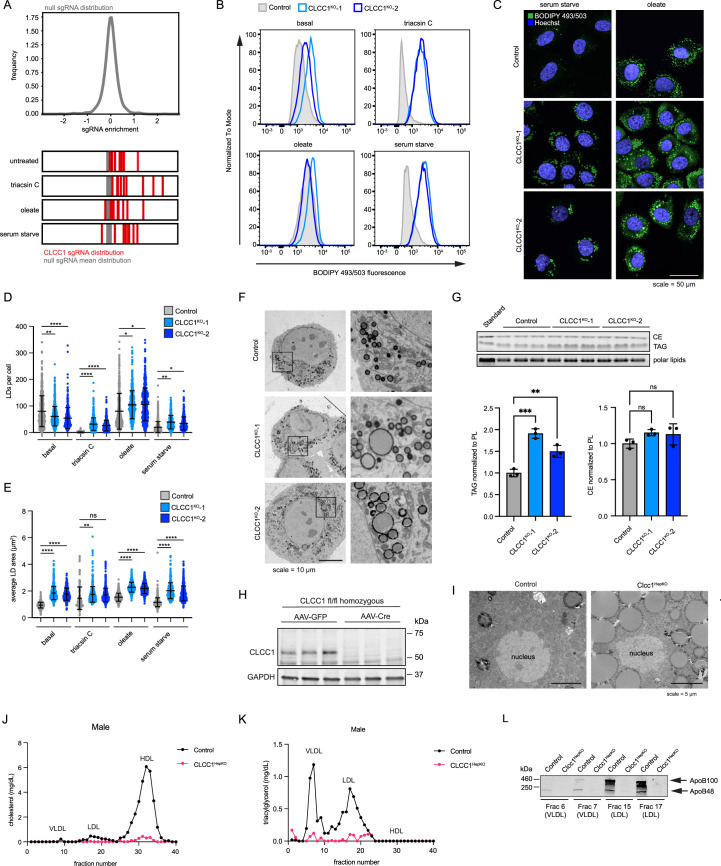
Loss of CLCC1 results in the accumulation of neutral lipids and enlarged LDs. A) Histogram indicating the distribution of control (null) sgRNAs. Shown below is the relative enrichment of CLCC1 targeting sgRNAs (red lines) under untreated, triacsin C, oleate, and serum starve conditions. The gray line indicates the mean of the control sgRNA distribution. B) Representative flow cytometry histograms of control and CLCC1^KO^ Huh7 cells following treatment with 1 µg/mL triacsin C for 24 h, 100 µM oleate for 24 h, serum starve for 48 h, or basal levels. C) Representative confocal images of lipid droplets using BODIPY 493/503 in control (expressing safe targeting sgRNA) and CLCC1^KO^ cells under 100 µM oleate for 24 h or serum starve for 48 h (also see Fig. 1d for imaging of untreated and triacsin C treated cells). D) Quantification of the number of LDs from images shown in Fig. 1d and Extended Data Fig. 2c. Data represent mean ± SD of > 100 cells across three biological replicates. E) Quantification of the area of LDs from images shown in Fig. 1d and Extended Data Fig. 2c. Data represent mean ± SD of > 100 cells across three biological replicates. F) Representative transmission EM images of negative stained control and CLCC1^KO^ Huh7 cells. G) *Top:* TLC resolving of TAG, CE, and polar lipids in control and CLCC1^KO^ Huh7 cells. *Bottom:* Quantified TAG (left graph) and CE (right graph) bands normalized to phospholipids (PL). Data represent mean ± SD of three biological replicates. H) Immunoblot of three Clcc1 fl/fl mice injected with either AAV-GFP (control) or AAV-Cre (Clcc1^HepKO^). Samples were analyzed four weeks post-injection. I) Representative transmission EM images of negative stained control and Clcc1^HepKO^ mice. J,K) Plasma fractionation by FPLC from male control and Clcc1^HepKO^ mice measuring (J) cholesterol in all fractions and (K) TAGs in all fractions. (see Fig. 2g, h for female mouse data) L) Immunoblot of apoB48 and apoB100 in indicated fractions for VLDL-associated apoB and LDL-associated apoB. *P < 0.05, **P < 0.01, ***P < 0.001, ****P < 0.0001; NS, not significant (*P* ≥ 0.05) by one-way ANOVA with Dunnett’s multiple comparisons test. Source data

CLCC1 emerged as a priority candidate for characterization because: (1) it ranked highly as a core negative regulator (Fig. 1c and Extended Data Fig. 2a); (2) it clustered with known lipolysis regulator genes *ABHD5* and *PNPLA2* (Fig. 1c); and (3) genetic variants in *CLCC1* in humans are associated with serum lipid alterations (Supplementary Fig. 2). Knockout of *CLCC1* in Huh7 cells led to increased lipid storage (Extended Data Fig. 2b). *CLCC1*-knockout (*CLCC1*-KO) cells accumulated large LDs under multiple metabolic conditions and the LDs were stable following induction of lipolysis with the acyl-CoA synthetase inhibitor triacsin C (Fig. 1d and Extended Data Fig. 2c–f). *CLCC1*-KO cells exhibited an increased amount of TAG (Extended Data Fig. 2g) as a result of both increased TAG biosynthesis and decreased TAG breakdown (Supplementary Fig. 3a,b), suggesting alterations of multiple aspects of neutral lipid metabolism.

## *Clcc1* loss disrupts hepatic lipid homeostasis

**Fig. 2:**
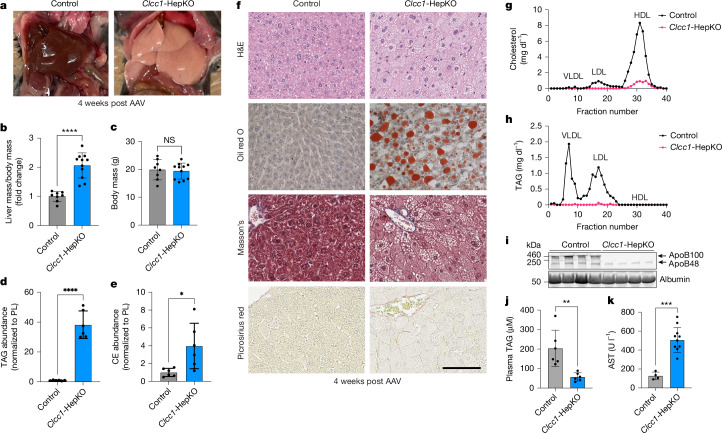
Liver-specific deletion of CLCC1 results in hepatic steatosis and reduced lipoprotein secretion. **a**, Representative images of livers from control and *Clcc1*-HepKO mice. **b**, Change in liver mass normalized to body mass for control and *Clcc1*-HepKO mice. *n* > 8. **c**, Body mass of the indicated control and *Clcc1*-HepKO mice. *n* > 8. **d**,**e**, Quantification of TAG (**d**) and cholesteryl ester (CE) (**e**) normalized to phospholipid (PL) content using thin layer chromatography (TLC). Data represent mean ± s.d. of six mice. **f**, Representative haematoxylin and eosin (H&E), oil red O, Masson’s trichrome and picrosirius red-stained liver sections from control and *Clcc1*-HepKO mice. Scale bar, 100 μm. **g**,**h**, Plasma fractionation by fast protein liquid chromatography (FPLC) from female control and *Clcc1*-HepKO mice measuring cholesterol (**g**) and TAG (**h**) in all fractions. LDL, low density lipoprotein. **i**, Western blot analysis of apoB and albumin in plasma from four female control mice and four *Clcc1*-HepKO mice. **j**, Quantification of TAG in plasma using TAG-Glo Assay (Promega, J3160). Data represent mean ± s.d. of six mice. **k**, Quantification of AST in serum of wild-type and *Clcc1*-HepKO mice. Data represent mean ± s.d. of more than four mice. *P < 0.05, **P < 0.01, ***P < 0.001, ****P < 0.0001; NS, not significant (*P* ≥ 0.05) by one-way ANOVA with Dunnett’s multiple comparisons test. Source data

*Clcc1* deletion is embryonic lethal in mice^9^. A spontaneous recessive mutation in mouse *Clcc1* and conditional knockout of mouse *Clcc1* cause ER stress and neurodegeneration^10,11^. However, the role of *Clcc1* in hepatocytes is unknown. Floxed *Clcc1* mice were injected with AAV8-TBG-Cre to delete *Clcc1* in hepatocytes (*Clcc1*-HepKO) (Extended Data Fig. 2h). Hepatic loss of *Clcc1* resulted in liver steatosis characterized by enlarged, whitened livers (Fig. 2a–c) with an increase in TAG and cholesteryl esters (Fig. 2d,e). Histology and electron microscopy revealed LD accumulation without evidence of fibrosis (Fig. 2f and Extended Data Fig. 2i). Analysis of plasma indicated a decrease in TAG and lipoproteins in the *Clcc1*-HepKO mice and a near complete abolishment of plasma apolipoprotein B (apoB)-containing lipoproteins (Fig. 2g–j and Extended Data Fig. 2j–l). However, plasma albumin levels were unchanged (Fig. 2i), indicating that not all secretion was impaired. Serum levels of aspartate aminotransferase (AST), a marker of liver damage, were also increased in the *Clcc1*-HepKO mice (Fig. 2k and Supplementary Fig. 3c). These findings demonstrate that CLCC1 has an important role in the regulation of hepatic lipid storage and secretion, and thereby protects hepatocytes from lipotoxicity.

## CLCC1 loss traps LDs in the ER

**Fig. 3:**
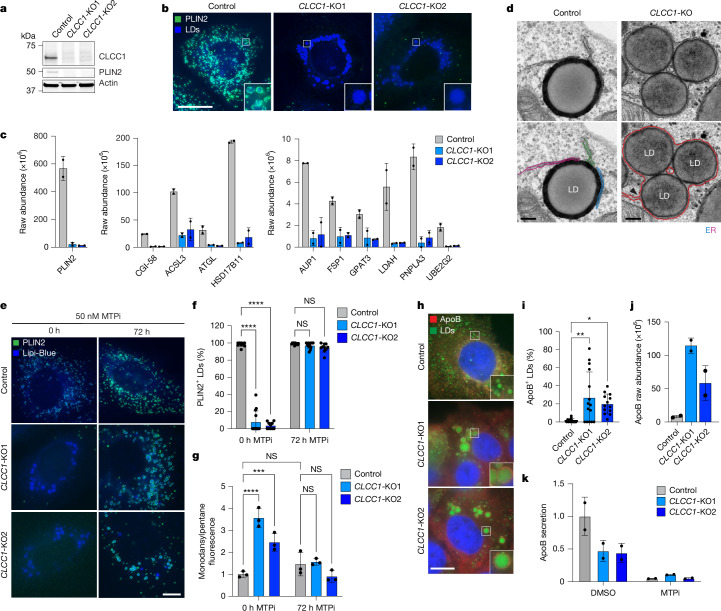
*CLCC1*-KO cells accumulate aberrant lipoproteins in the ER lumen. **a**, Immunoblot of PLIN2 in the indicated Huh7 cell lines. **b**, Fluorescence microscopy images of indicated Huh7 cell lines. PLIN2 was labelled with anti-PLIN2 and LDs were stained with 500 nM Lipi-Blue. Scale bar, 20 µm. **c**, Raw abundance values (in arbitrary units) for selected LD proteins obtained using proteomic analyses of buoyant LD-enriched fractions. Two technical replicates. **d**, Transmission electron microscopy of indicated negative-stained Huh7 cell lines. Scale bar, 200 nm. **e**, Fluorescence microscopy images of cells treated with 50 nM of the MTP inhibitor (MTPi) CP-346086 or 72 h. PLIN2 was labelled with rabbit anti-PLIN2 and LDs were stained with 500 nM Lipi-Blue. Scale bar, 10 µm. **f**, Quantification of PLIN2-positive LDs in **e**. *n* > 10 cells. **g**, Quantification of monodansylpentane fluorescence using flow cytometry. **h**, Fluorescence microscopy images of indicated cells. ApoB was labelled with anti-apoB and LDs were stained with 500 nM Lipi-Blue. Scale bar, 20 µm. **i**, Proportion of apoB-positive LDs in **h**. *n* > 10 cells. **j**, Raw abundance of apoB obtained using proteomic analyses of buoyant LD-enriched fractions. Two technical replicates. **k**, Quantification of secreted apoB in medium using ELISA. Two biological replicates. One-way ANOVA with Dunnett’s multiple comparisons test. *P < 0.05, **P < 0.01, ***P < 0.001, ****P < 0.0001; NS, not significant (*P* ≥ 0.005). Source data

**Extended Data Fig. 3:**
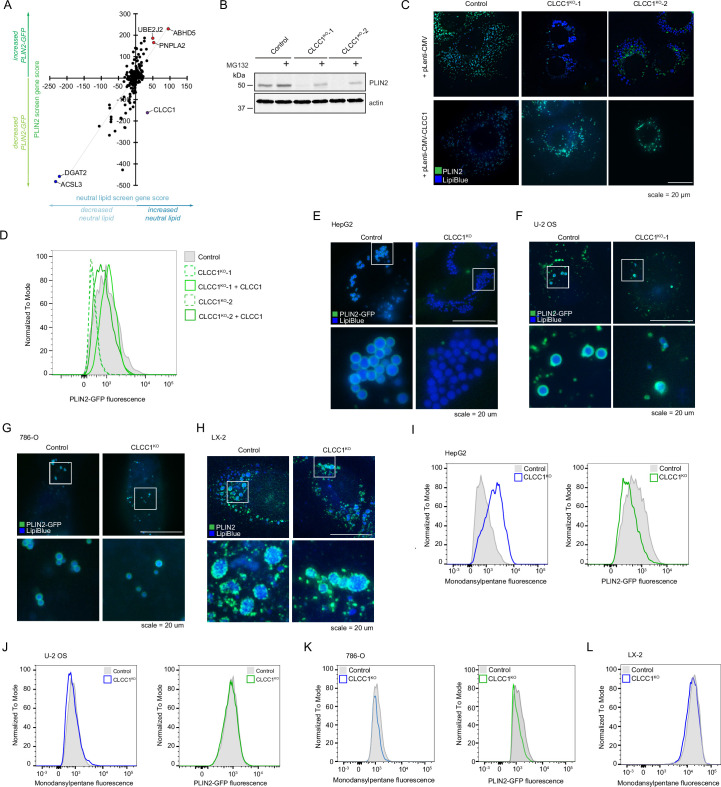
Loss of CLCC1 results in the accumulation of PLIN2-negative lipid droplets. A) Pairwise comparison of the BODIPY 493/503 neutral lipid batch retest screen (Fig. 1c and Supplementary Table 1) and a prior PLIN2-GFP batch retest screen^8^. B) Immunoblot of PLIN2-GFP levels in control and CLCC1^KO^ cells treated with 1 µM MG132 for 12 h. C) Representative fluorescence microscopy images of PLIN2-GFP in control and CLCC1^KO^ Huh7 cell lines expressing a control plasmid or an untagged CLCC1 expression plasmid. LDs are stained with LipiBlue (blue). Scale bar represents 20 µm. D) Flow cytometry histograms of PLIN2-GFP fluorescence in control and CLCC1^KO^ Huh7 cells expressing a control plasmid (dashed) or an untagged CLCC1 expression plasmid (solid). E-H) Representative fluorescence microscopy images of (E) PLIN2-GFP HepG2, (F) PLIN2-GFP U-2 OS, (G) PLIN2-GFP 786-O, and (H) LX-2 cells expressing a safe targeting sgRNA (control) or sgRNAs against CLCC1. LDs were stained with 500 nM Lipi-Blue and PLIN2 was stained with rabbit anti-PLIN2 (Abcepta) in LX-2 cells. Scale bar represents 20 µm. I-L) Flow cytometry histograms of PLIN2 and neutral lipid levels (monodansylpentane fluorescence) in (I) PLIN2-GFP HepG2, (J) PLIN2-GFP U-2 OS, (K) PLIN2-GFP 786-O, and (L) LX-2 cells expressing a safe targeting sgRNA (control) or sgRNAs against CLCC1.

In a pairwise comparison of the previously published PLIN2–GFP^8^ and current BODIPY 493/503 screens, CLCC1 was an outlier; CLCC1 knockout was linked to an increase in neutral lipids but a counterintuitive decrease in PLIN2–GFP levels (Extended Data Fig. 3a). This was surprising because members of the perilipin family of LD ‘coat’ proteins are constitutively present on LDs^12^ and the amount of PLIN2 generally correlates with LD abundance^8,13^. PLIN2 protein was undetectable in *CLCC1*-KO cells (Fig. 3a). Incubation with the proteasome inhibitor MG132 rescued PLIN2 expression, indicating that despite apparent high amounts of LDs, PLIN2 is degraded post-translationally by the proteasome in *CLCC1*-KO cells (Extended Data Fig. 3b). Immunofluorescence staining of PLIN2 also revealed a strong reduction in PLIN2-positive LDs in the *CLCC1*-KO cells, with large LDs appearing to be completely devoid of any PLIN2 staining (Fig. 3b). Overexpression of CLCC1 rescued PLIN2–GFP localization to LDs (Extended Data Fig. 3c) and the reduction in PLIN2–GFP levels (Extended Data Fig. 6d) in *CLCC1*-KO cells, consistent with these phenotypes reflecting on-target depletion of CLCC1. Proteomics analyses of LD-enriched buoyant fractions validated the decrease in PLIN2 on LDs and revealed the decrease in many well known LD proteins (Fig. 3c and Supplementary Table 3). These results indicate that although *CLCC1*-KO cells appear to accumulate large LDs, they lack canonical LD proteins.

To examine the generalizability of this phenotype, we generated *CLCC1*-KO HepG2 (hepatocellular carcinoma), 786-O (renal cell carcinoma), LX-2 (hepatic stellate cell) and U-2 OS (osteosarcoma) cell lines. The *CLCC1*-KO HepG2 cells exhibited enlarged PLIN2-negative LDs (Extended Data Fig. 3e), decreased PLIN2–GFP levels (Extended Data Fig. 3i) and an increase in neutral lipid staining (Extended Data Fig. 3i). By contrast, LDs in the non-hepatocyte cell types were of the expected size and were PLIN2-positive (Extended Data Fig. 3f–h), and the amounts of PLIN2 and neutral lipid were unchanged (Extended Data Fig. 3j–l). These data suggest that the observed LD phenotypes are restricted to hepatocytes.

**Extended Data Fig. 4:**
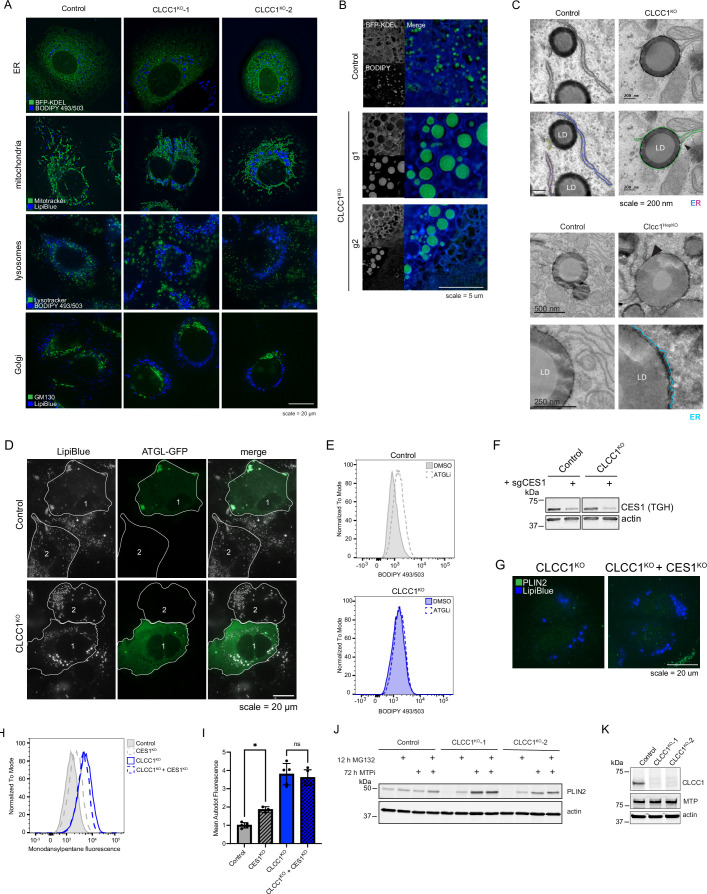
Analysis of organelle architecture and lipase functions in CLCC1 knockout cell lines. A) Representative fluorescence microscopy images of mitochondria, lysosomes, Golgi, and LDs in control and CLCC1^KO^ Huh7 cells. Mitochondria was labeled with 500 nM Mitotracker green, lysosomes were labeled with 75 nM Lysotracker DND-22, the Golgi apparatus was labeled with rabbit anti-GM130 antibody, and LDs were stained with either 500 nM Lipi-Blue or 1 µg/mL BODIPY 493/503. Scale bar represents 20 µm. B) Fluorescence imaging of ER and LDs in control and CLCC1^KO^ Huh7 cells. Two KO lines generated with independent sgRNA (g1 and g2) were analyzed. ER is labeled with a transiently transfected BFP-KDEL construct and LDs were stained with 1 µg/mL BODIPY 493/503. Scale bar represents 5 µm. C) *Top*: Transmission EM of negative stained control and CLCC1^KO^ Huh 7 cells. ER is highlighted in color and bifurcations, locations where the ER separates and a bilayer is found to encircle LDs, are denoted with black arrows. Scale bar represents 200 nm. *Bottom*: Transmission EM of negative stained control and Clcc1^HepKO^ mouse liver. ER is highlighted in color and bifurcations are denoted with black arrows. Scale bar represents 250 or 500 nm as indicated. D) Representative fluorescence images of control and CLCC1^KO^ Huh7 cells expressing ATGL-GFP. LDs were stained with 500 nM Lipi-Blue. Scale bar represents 20 µm. E) Flow cytometry histograms of BODIPY 493/503 fluorescence in control and CLCC1^KO^ Huh7 cells treated with DMSO (solid) or 50 nM of the ATGL inhibitor NG-497 (ATGLi) for 24 h (dashed). F) Immunoblot of CES1 (i.e. TGH) knockdown efficiency in control and CLCC1^KO^ cells. G) CES1/TGH KO and imaging of PLIN2-GFP and LDs. H) Flow cytometry histograms of monodansylpentane fluorescence in control, CES1^KO^, CLCC1^KO^, and CLCC1^KO^/CES1^KO^ cells. I) Quantification of (H). Data represent mean ± SD across three biological replicates. *P < 0.05 by one-way ANOVA with Dunnett’s multiple comparisons test. NS, not significant (*P* ≥ 0.05). J) Immunoblot of PLIN2-GFP levels in control and CLCC1^KO^ cells treated with 1 µM MG132 for 12 h and/or 50 nM MTPi for 72 h. K) Immunoblot of MTP levels in control and CLCC1^KO^ cells. Source data

We examined the distribution of several organelles and their relationship with LDs in the *CLCC1*-KO cells by immunofluorescence. The distribution and morphology of the Golgi, lysosomes and mitochondria were unchanged (Extended Data Fig. 4a). However, the ER exhibited an atypical morphology, which encircled the enlarged LDs in *CLCC1*-KO cells (Extended Data Fig. 4a,b). Under electron microscopy, we observed the expected ER–LD contact sites in control cells and mouse liver (Fig. 3d and Extended Data Fig. 4c). By contrast, in *CLCC1*-KO cells and *Clcc1*-HepKO mice, LDs were surrounded by their typical monolayer as well as a secondary bilayer membrane (Fig. 3d and Extended Data Fig. 4c). In some cases, we observed an ER sheet that was connected to the bilayer encircling one or more LDs (Fig. 3d). These data indicate that LDs in *CLCC1*-KO cells emerged into the ER lumen, where they are spatially segregated from cytoplasmic LD proteins. Consistent with this model, overexpression of the LD lipase ATGL led to the consumption of LDs in control cells but not *CLCC1*-KO cells (Extended Data Fig. 4d), and inhibition of ATGL or knockout of the ER lipase CES1 increased neutral lipid content in control cells but had no effect in *CLCC1*-KO cells (Extended Data Fig. 4e–i). These data demonstrate that the LDs in *CLCC1*-KO cells accumulate in the ER lumen and are inaccessible to ER and cytoplasmic lipases.

## LDs in *CLCC1*-KO hepatocytes are MTP-dependent

A unique aspect of hepatocytes is their ability to form and secrete lipoproteins (VLDLs)^1,6^. Similar to LDs, lipoproteins consist of a neutral lipid core surrounded by a phospholipid monolayer embedded with functionally important proteins, such as apoB^6^. The formation of lipoproteins involves microsomal TAG transfer protein (MTP)-mediated transfer of TAG into the ER lumen^6^. Incubation of *CLCC1*-KO cells with the MTP inhibitor CP-346086 rescued the biogenesis of PLIN2-positive LDs (Fig. 3e,f), PLIN2 levels (Extended Data Fig. 4j) and the amount of neutral lipids (Fig. 3g). MTP levels were unchanged in *CLCC1*-KO cells (Extended Data Fig. 4k). We observed an increased percentage of apoB-positive LDs in the *CLCC1*-KO cells (Fig. 3h,i) and higher apoB peptide counts in buoyant, LD-enriched fractions (Fig. 3j). ApoB secretion into the medium by *CLCC1*-KO Huh7 cells was reduced (Fig. 3k), whereas the levels of albumin in the medium were unaltered (Supplementary Fig. 4a). In addition, a luciferase-based assay of ER protein folding and secretion^14,15^ showed no impairment in the *CLCC1*-KO cells (Supplementary Fig. 4b).

Together, these findings indicate that the enlarged LDs in *CLCC1*-KO hepatocytes are aberrant lipoproteins that exhibit reduced secretion. The near complete lack of cytoplasmic PLIN2-positive LDs suggests a shift in overall neutral lipid flux away from cytoplasmic LDs towards lumenal MTP-dependent lipoproteins. It is noteworthy that VLDLs typically have a diameter of 50–80 nm, whereas the mean diameter of the lumenal LDs in the *CLCC1*-KO cells was 1.84 µm. Thus, the lipoproteins that accumulate in the *CLCC1*-KO cells are exceptionally large compared with normally secreted VLDL particles (more than 10,000-fold larger).

## Potential link between CLCC1 and ER scramblases

Loss of CLCC1 has been associated with an increase in ER stress^10,11^. However, there was no increase in mRNA transcripts of common ER stress targets (Supplementary Fig. 4c) or protein levels of the ER chaperone grp78 (also known as BiP) (Supplementary Fig. 4d) in *CLCC1*-KO cells. Although induction of ER stress by treatment with tunicamycin and thapsigargin altered LD distribution in control cells, the LDs remained PLIN2–GFP positive (Supplementary Fig. 4e). These findings indicate that ER stress is not necessary for the formation of lumenal LDs in *CLCC1*-KO cells and is insufficient to trigger the accumulation of ER lumenal LDs.

**Extended Data Fig. 5:**
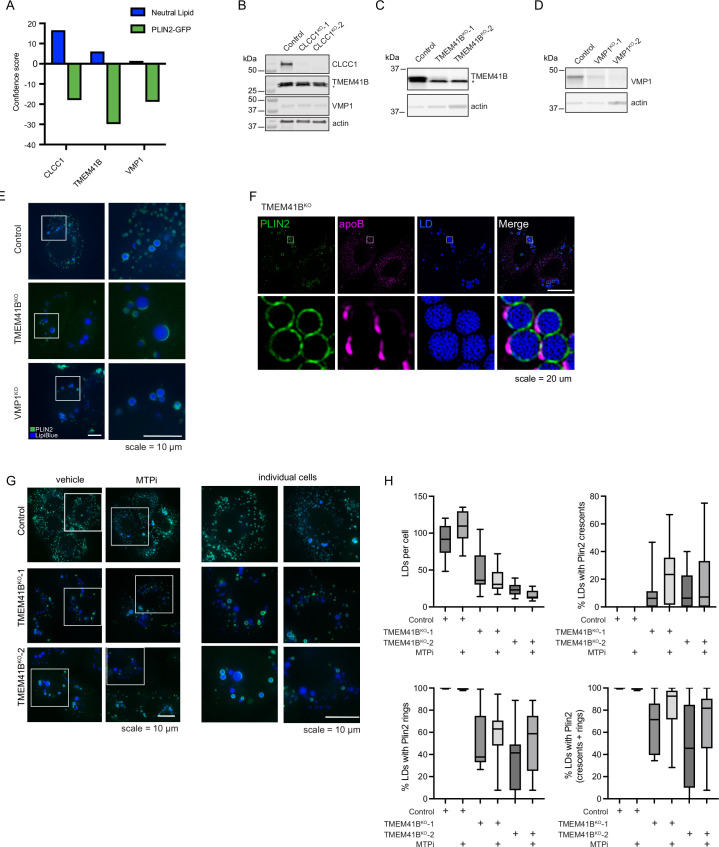
Analysis of ER scramblases and lumenal lipid droplets. A) Confidence score for CLCC1, TMEM41B, and VMP1 from two genome wide CRISPR screens, the neutral lipid (i.e., BODIPY 493/503) screen in the current manuscript and a previous PLIN2-GFP screen^8^. The sign, positive or negative, indicates the effect of gene depletion on neutral lipids or PLIN2-GFP levels. B) Immunoblot of the indicated proteins in control and CLCC1^KO^ Huh7 cells. C) Immunoblot of TMEM41B in control and TMEM41B^KO^ Huh7 cells. D) Immunoblot of VMP1 in control and VMP1^KO^ Huh7 cells. E) Representative fluorescence microscopy images of PLIN2 (green) and LDs (blue) in control, TMEM41B^KO^ and VMP1^KO^ Huh7 cells. Scale bar represents 10 µm. F) Representative image of PLIN2 (green) and apoB (purple) crescents on an LD in TMEM41B^KO^ Huh7 cells. Scale bar represents 20 µm. G) Representative fluorescence microscopy images of PLIN2 (green) and LDs (blue) in control and TMEM41B^KO^ Huh7 cells treated with 50 nM CP-346086 (MTPi) for 72 h. Zoom images of the boxed regions are shown on the right. Scale bar represents 10 µm. H) Quantification of LDs and LD PLIN2 staining of control and TMEM41B^KO^ Huh7 cells incubated in with DMSO or 50 nM CP-346086 (MTPi) as in panel F. Data represent mean ± SD across three biological replicates. Source data

In our genome-wide screens, the depletion of *TMEM41B* was associated with an increase in neutral lipids and a decrease in PLIN2–GFP (Extended Data Fig. 5a), similar to our observations with *CLCC1* depletion. TMEM41B is an ER scramblase that interacts with apoB and regulates hepatic lipoprotein secretion^16^. Knockout of *Tmem41b* in mice reduces lipoprotein secretion and results in severe hepatic steatosis and the accumulation of LDs encapsulated by ER membranes^16^. Knockout of a second ER scramblase gene, *Vmp1*, causes hepatic steatosis and the buildup of PLIN2-negative LDs, similarly suggesting an association between phospholipid scrambling and ER lumenal LDs in hepatocytes^17,18^. However, loss of *VMP1* in our screens was associated with a reduction in PLIN2–GFP but no change in neutral lipid staining (Extended Data Fig. 5a).

There was no change in the levels of TMEM41B or VMP1 in *CLCC1*-KO cells (Extended Data Fig. 5b). LDs were PLIN2-positive (that is, cytoplasmic) in the *VMP1*-knockout cells (Extended Data Fig. 5c–e). A portion of LDs in *TMEM41B*-knockout (*TMEM41B*-KO) cells was enlarged and PLIN2-negative (Extended Data Fig. 5e). However, in contrast to *CLCC1*-KO cells, *TMEM41B*-KO cells often exhibited PLIN2 crescent staining (Extended Data Fig. 5e). A portion of these LDs also stained positive for apoB (Extended Data Fig. 5f), suggesting that a fraction of LDs in the *TMEM41B*-KO cells is trapped in the membrane with half of the LD facing the cytoplasm and the other half facing the ER lumen. In addition, although MTP inhibition completely rescued LD cytoplasmic emergence in the *CLCC1*-KO cells (Fig. 3e,f), MTP inhibition only had a partial effect on *TMEM41B*-KO cells and the effect was not significant (Extended Data Fig. 5g,h). Finally, overexpression of TMEM41B or VMP1 in *CLCC1*-KO cells had no effect on the amount of PLIN2-negative and PLIN2-positive LDs (Supplementary Fig. 5a–c). Thus, cells lacking CLCC1 and TMEM41B exhibit similar phenotypes and suggest a possible functional relationship, which is supported by recent findings^19^. However, our data suggest that dysregulation of ER scramblases alone is not sufficient to account for the profound alterations in neutral lipid flux in the *CLCC1*-KO cells.

## CLCC1 is structurally related to Brl1 and Brr6

CLCC1 has been suggested to be a chloride channel^10,20^. However, there is no conclusive evidence for ion conductance by CLCC1, it has no overall sequence similarity with known channel families^20^ and its predicted AlphaFold structure is markedly different from those of canonical chloride intracellular channels (Supplementary Fig. 6a,b). Thus, we considered the possibility that CLCC1 has alternative biochemical functions.

**Fig. 4:**
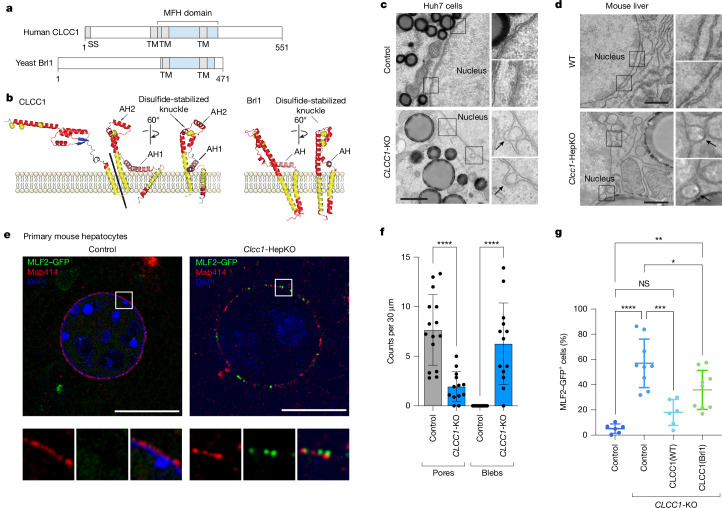
CLCC1 is the human orthologue of yeast Brl1 and promotes nuclear pore assembly. **a**, Domain structure of human CLCC1 and yeast Brl1 indicating transmembrane domains (TM), signal sequence (SS) and the homologous MFH domain (blue). **b**, AlphaFold structures of human CLCC1 and the homologous region of yeast Brl1. Ribbon colours: yellow, TMH; pink, amphipathic helix (AH); red, other helix; blue, sheet. Yellow spheres represent conserved cysteines. **c**,**d**, Transmission electron microscopy of negative-stained control and *CLCC1*-KO Huh7 cells (**c**) and wild-type (WT) and *Clcc1*-HepKO mouse liver (**d**). Closer views (right) highlight the nuclear envelope ultrastructure. Scale bars, 1 μm. **e**, Superresolution imaging of FG-rich nucleoporins (labelled with Mab414), nuclear blebs (labelled with MLF2–GFP) and nuclei (DAPI) in control and *Clcc1*-KO primary mouse hepatocytes. Scale bars, 10 µm. **f**, Quantification of nuclear pores and blebs from electron microscopy of the indicated cells. Data represent mean ± s.d. of more than 15 sections across 5 cells. **g**, Quantification of MLF2–GFP puncta in control and *CLCC1*-KO cells that were untransfected or transfected with wild-type CLCC1 or a chimeric CLCC1 containing the shared domain of Brl1 (CLCC1(Brl1)). Data represent mean ± s.d. of more than six replicates. One-way ANOVA with Dunnett’s multiple comparisons test. *P < 0.05, **P < 0.01, ***P < 0.001, ****P < 0.0001; NS, not significant (*P *≥ 0.05). Source data

An HHpred search for remote homologues, which takes into account sequence and structural homology, revealed a strong relationship between amino acids 204–378 of CLCC1 with the yeast paralogues Brl1 (probability score 94%) and Brr6 (probability score 86%) (Fig. 4a and Supplementary Fig. 7). The AlphaFold predicted structures show a homology domain with similar features (Fig. 4b). Both predicted structures contain an elongated transmembrane helix (TMH) with a portion of the helix entering deep into the ER lumen, a sharp turn followed by an anti-parallel helix (knuckle region), a perpendicular amphipathic helix and another TMH. The knuckle region of Brl1 and Brr6 contains two cysteine pairs that stabilize the structure with intramolecular disulfide bonds^21^, and the knuckle of CLCC1 contains a cysteine pair plus an extra short amphipathic helix (AH2) (Fig. 4b). Co-immunoprecipitation data with differentially tagged CLCC1 indicated that CLCC1 self-associates^10^, and proteome-scale predictions of homo-oligomeric assemblies predict a CLCC1 dimer that is stabilized by intermolecular disulfides in a lumenal N-terminal domain not shared with Brl1 and Brr6^22^ (Supplementary Fig. 8a,b). Consistent with this model, we find that CLCC1 migrates as a dimer and addition of the reducing agent dithiothreitol yields the expected CLCC1 monomer (Supplementary Fig. 8c). Thus, CLCC1 is structurally homologous to yeast Brl1 and Brr6 but exists as a disulfide-stabilized dimer.

## CLCC1 is essential for NPC assembly

Brl1 and Brr6 function in the assembly of nuclear pore complexes (NPCs), which are large nuclear envelope protein complexes that perforate the inner and outer membranes of the nuclear envelope to enable exchange between the nucleus and cytoplasm^21,23–27^. Although the architecture of NPCs has been determined^28^, the mechanism of NPC membrane insertion remains incompletely understood. Conditional disruptions in *BRL1* and *BRR6*, both of which are essential in yeast, result in nuclear membrane herniations (often referred to as nuclear blebs) indicative of disruptions in NPC insertion^21,23–27^. It is noteworthy that there is precedent for factors that have roles in both NPC assembly and hepatic neutral lipid storage. The conditional loss of the ER lumenal AAA ATPase torsinA, or its cofactors Lull1 or Lap1, result in defects in NPC biogenesis and nuclear membrane herniations as well as hepatic steatosis and reduced lipoprotein secretion^29,30^.

**Extended Data Fig. 6:**
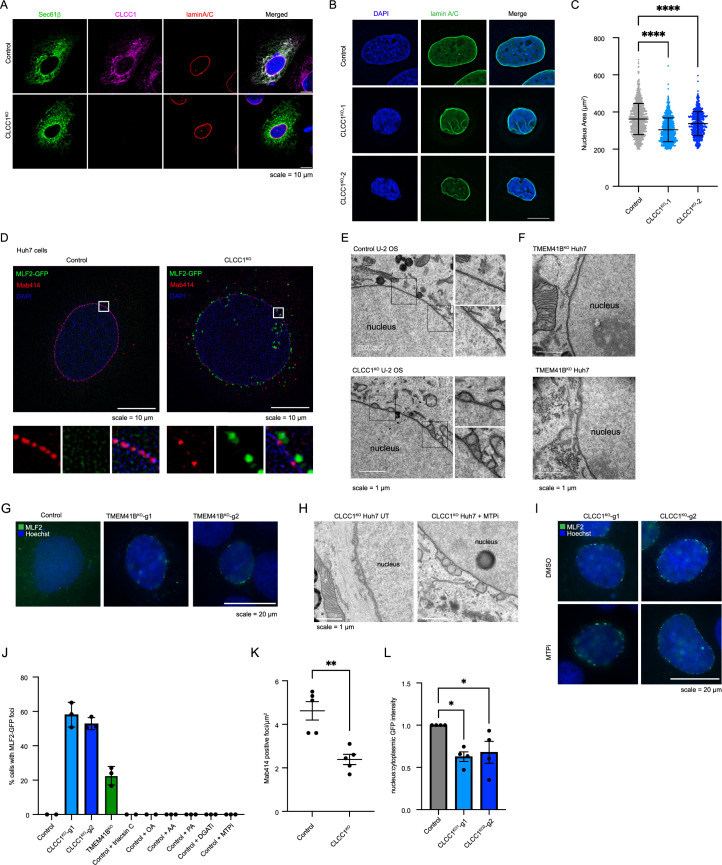
Analyses of nuclear architecture and nucleocytoplasmic transport. A) Confocal fluorescence microscopy images of CLCC1 (purple), the ER marker Sec61β (green), the nuclear membrane marker lamin A/C (red), and NuclearMask in control and CLCC1^KO^ Huh7 cells. B) Representative fluorescence microscopy images of nuclei (blue) and lamin A/C (green) in control and CLCC1^KO^ Huh7 cells. C) Quantification of nucleus area by DAPI fluorescence in control and CLCC1^KO^ Huh7 cells. Data represent mean ± SD of > 500 cells across three biological replicates. ****p < *0.0001 by one-way ANOVA with Dunnett’s multiple comparisons test*. D) Superresolution imaging of FG-rich nucleoporins (red), nuclear blebs (green), and nuclei (blue) in control and CLCC1^KO^ Huh7 cells. Insets show juxtaposition of NPC components at the nuclear membrane and nuclear blebs budding into the cytosol. Scale bar represents 10 µm. E) Transmission EM of negative stained control and CLCC1^KO^ U-2 OS cells. F) Transmission EM of negative stained control and TMEM41B^KO^ Huh7 cells. G) Fluorescence imaging of MLF2-GFP (nuclear bleb marker) in control and TMEM41B^KO^ Huh7 cells.H) Transmission EM of negative stained CLCC1^KO^ Huh7 cells treated in the presence and absence of CP-346086 (MTPi) for 72 h. I) Fluorescence imaging of MLF2-GFP (nuclear bleb marker) in CLCC1^KO^ Huh7 cells treated in the presence and absence of CP-346086 (MTPi) for 72 h. J) Quantification of % cells with MLF2-GFP foci in control, CLCC1^KO^, or TMEM41B^KO^ Huh7 cells treated with fatty acids or drugs to alter lipid metabolism. Data represent mean ± SD of > two biological replicates. K) Quantification of nuclear pores per µm^2^ of nuclear membrane from (Supplementary Fig. 10a). Data represent mean ± SD of five cells. L) Quantification of nuclear GFP intensity:cytoplasmic GFP intensity from (Supplementary Fig. 10b). Data represent mean ± SD of four biological replicates. *P < 0.05, **P < 0.01, ***P < 0.001, ****P < 0.0001 by one-way ANOVA with Dunnett’s multiple comparisons test. Source data

Given the structural homology between CLCC1 with yeast Brl1 and Brr6, we examined a potential relationship of CLCC1 with nuclear structure. Co-essentiality analyses^31^ indicate a functional relationship between CLCC1 and NPC genes (Supplementary Fig. 9a) and proteomic analyses of isolated organelles^32^ indicate that CLCC1 is present in the ER, with similar localization profiles as the torsin activators Lap1 (also known as TOR1AIP1) and Lull1 (also known as TOR1AIP2) as well as the scramblases TMEM41B and VMP1 (Supplementary Fig. 9b). Consistent with these data and past studies^9,11^, endogenous CLCC1 localized to both the ER and nuclear envelope (Extended Data Fig. 6a). Notably, *CLCC1*-KO cells showed alterations in lamin A/C staining, nuclear morphology and nuclear size (Extended Data Fig. 6b,c). Electron microscopy also revealed nuclear envelope herniations in cultured *Clcc1*-KO cells (Fig. 4c) and in liver tissue from *Clcc1*-HepKO mice (Fig. 4d). Nuclear envelope herniations occur when the inner and outer membranes of the nuclear envelope fail to fuse during NPC insertion, resulting in membrane protrusions that contain extruded nucleoplasm. Consistent with this structure, puncta of GFP-tagged MLF2, a marker of nuclear envelope herniations that accumulates in the nucleoplasmic interior of the nuclear envelope herniation^33^, decorated the nuclear envelope of *Clcc1*-HepKO hepatocytes and *CLCC1*-KO Huh7 cells but not that of control cells (Fig. 4e and Extended Data Fig. 6d). Knockout of *CLCC1* in the osteosarcoma cell line U-2 OS also led to the accumulation of nuclear envelope herniations (Extended Data Fig. 6e), indicating a generalizable role of CLCC1 in non-hepatocyte cells. Nuclear envelope herniations were observed in *TMEM41B*-KO cells, although they were less abundant compared with the *CLCC1*-KO cells (Extended Data Fig. 6f,g). MTP inhibition had no effect on the nuclear envelope herniations in the *CLCC1*-KO cells (Extended Data Fig. 6h,i) and the addition of fatty acids or inhibition of acyl-CoA synthetases or DGAT enzymes did not induce nuclear envelope herniations (Extended Data Fig. 6j). These data suggest that nuclear envelope herniations in *CLCC1*-KO cells are not the result of altered ER neutral lipid flux or the presence of ER lumenal LDs.

The presence of nuclear envelope herniations are consistent with a defect in NPC assembly. Indeed, in *Clcc1*-HepKO hepatocytes, MLF2–GFP marked herniations were often associated with Mab414 immunostaining of nucleoporins containing FxFG repeats (Fig. 4e and Extended Data Fig. 6d). Furthermore, *CLCC1*-KO cells exhibited a reduction in NPC amount and in nucleocytoplasmic transport (Fig. 4f, Extended Data Fig. 6k,l and Supplementary Fig. 10a,b). To explore the evolutionary relationship between CLCC1 and Brl1 we tested the effect of expressing a CLCC1–Brl1 chimera, in which their shared domain is swapped. The CLCC1–Brl1 chimera partially rescued the nuclear envelope herniations marked by MLF2–GFP but did not rescue neutral lipid levels or PLIN2-positive LDs (Fig. 4g and Supplementary Fig. 11a–d). The expression of the CLCC1–Brl1 chimera was lower than that of wild-type CLCC1, potentially accounting for the partial rescue. Together, our findings implicate CLCC1 as the human homologue of Brl1 and Brr6 that promotes NPC assembly.

## CLCC1 functions in hepatic NPC assembly

**Fig. 5:**
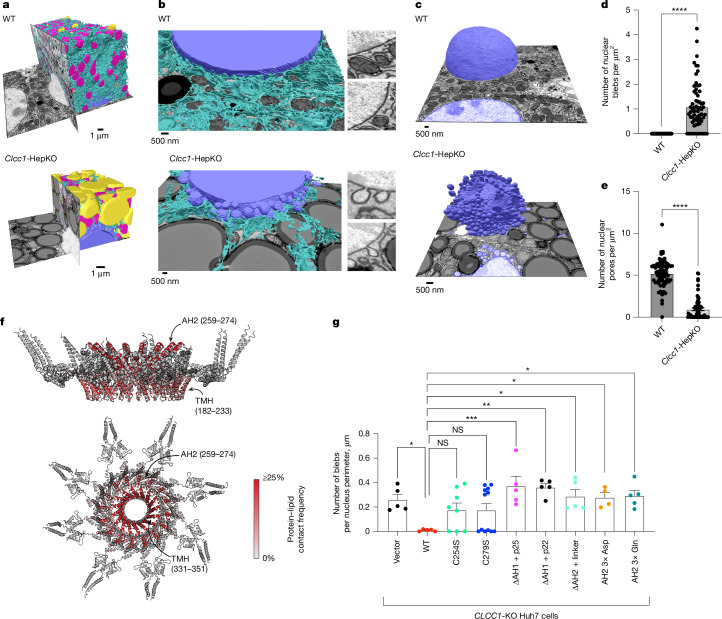
CLCC1 facilitates nuclear pore insertion and MTP-dependent neutral lipid channelling. **a**, Partial reconstruction of segmented raw FIB-SEM data with ER (cyan), mitochondria (magenta), nuclei (blue) and LDs (yellow) from hepatocytes of wild-type (top) and *Clcc1*-HepKO (bottom) mice. **b**, Magnified views of reconstructions in **a** show examples of nuclear membrane in the wild-type (top) and *Clcc1*-HepKO (bottom) mice. **c**, Partial reconstruction of segmented raw FIB-SEM data with nuclei (blue), from hepatocytes of wild-type (top) and *Clcc1*-HepKO (bottom) mice. **d**,**e**, Quantification of nuclear blebs (**d**) and nuclear pores (**e**) from FIB-SEM of control and *Clcc1*-HepKO hepatocytes. Nuclei were segmented into 1 µm^2^ segments as described in Methods. Data represent mean ± s.e.m. ****P < 0.0001 by Student’s *t*-test. **f**, Ensemble averaged and normalized residue–lipid tail contact map derived from self-assembly coarse-grained molecular dynamics simulation (Supplementary Table 4a). The three main lipid-interacting regions are the TMHs (amino acids 182–233 and 331–351) and the lumenal amphipathic helix (259–274). **g**, Quantification of nuclear bleb counts (normalized to nuclear perimeter) for the indicated constructs. Each data point corresponds to a single image. Quantification was performed using the analysis pipeline outlined in Supplementary Fig. 13. Data represent mean ± s.e.m. One way ANOVA with Dunnett’s multiple comparison test. *P < 0.05, **P < 0.005, ***P < 0.001; NS, not significant (*P*  ≥ 0.05). Source data

**Extended Data Fig. 7:**
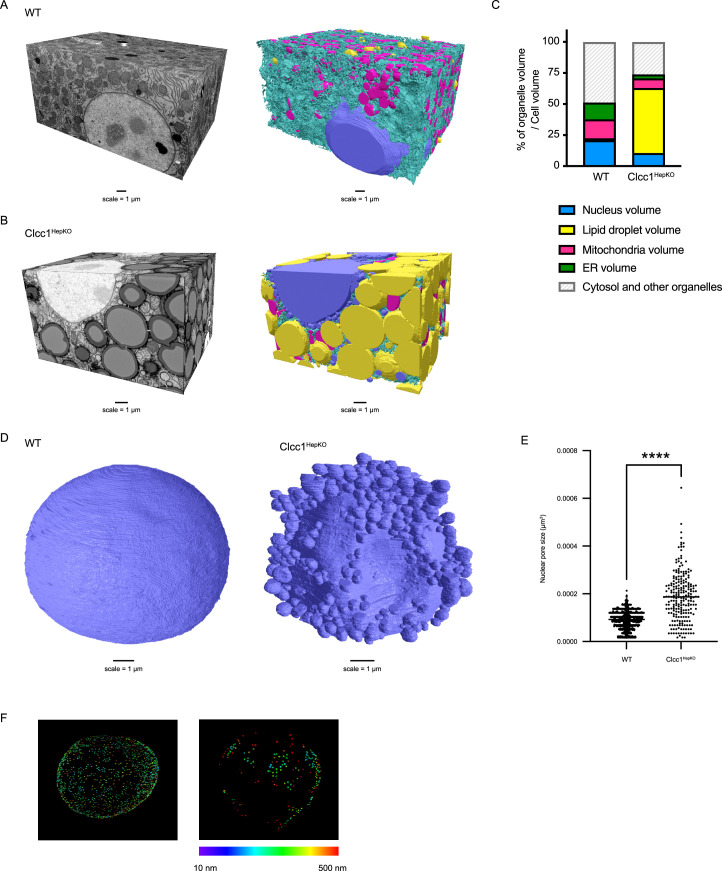
Analysis of organelle architecture using FIB-SEM. A,B) 3D reconstruction of FIB-SEM images and convolutional neural network based automated segmentation of liver volumes derived from liver volumes from WT (A) and Clcc1^HepKO^ (B) mice. ER (Cyan), mitochondria (magenta), LDs (yellow), and nucleus (purple). C) Quantification of organelle volume as a percentage of cellular volume in FIB-SEM reconstructions (A,B) from WT and Clcc1^HepKO^ mice. D) Reconstruction of segmented raw FIB-SEM data for nuclei (blue) from hepatocytes of WT and Clcc1^HepKO^ mice. E) Quantification of nuclear pore size from FIB-SEM of control and Clcc1^HepKO^ hepatocytes. Data represent mean ± SD of > 1771 WT NPCs and 223 KO NPCs. ****P < 0.0001 by Student’s *t*-test. F) Distribution of nuclear pores across the surface of the NE in control and Clcc1^HepKO^ hepatocytes. Color represents the average distance between each individual nuclear pore and its two nearest neighbors. Source data

To characterize the effect of Clcc1 on organelle architecture in hepatocytes, we performed focused ion beam scanning electron microscopy (FIB-SEM) analyses of liver tissue from control and *Clcc1*-HepKO mice (Fig. 5a–c, Extended Data Fig. 7a,b and Supplementary Videos 1 and 2). This analysis revealed the accumulation of enlarged LDs that occupied a considerable percentage of the cell volume at the expense of other organelles (Fig. 5a–c and Extended Data Fig. 7a–c). One of the most prominent phenotypes in the *Clcc1*-HepKO liver tissue was the presence of extensive nuclear envelope herniations decorating the majority of the nucleus (Fig. 5b,c and Extended Data Fig. 7d). Most of the blebs were connected to the nuclear envelope by a thin membrane stalk (Fig. 5b). We also observed large indentations in the nucleus with reduced amounts of nuclear envelope herniations that were generated by juxtanuclear LDs pressing into and deforming the nucleus (Fig. 5c and Extended Data Fig. 7d). Quantification confirmed the increase in nuclear envelope herniations (Fig. 5d) and decrease in nuclear pores in the hepatocytes from *Clcc1*-HepKO mice (Fig. 5e). The nuclear pore sizes were larger (Extended Data Fig. 7e) and the distribution of nuclear pores was altered (Extended Data Fig. 7f) in the *Clcc1*-HepKO mice, forming clusters of nuclear pores rather than the more even distribution observed in control mice. These findings demonstrate a role for CLCC1 in NPC assembly in vivo.

## Molecular dynamics of CLCC1 function

**Extended Data Fig. 8:**
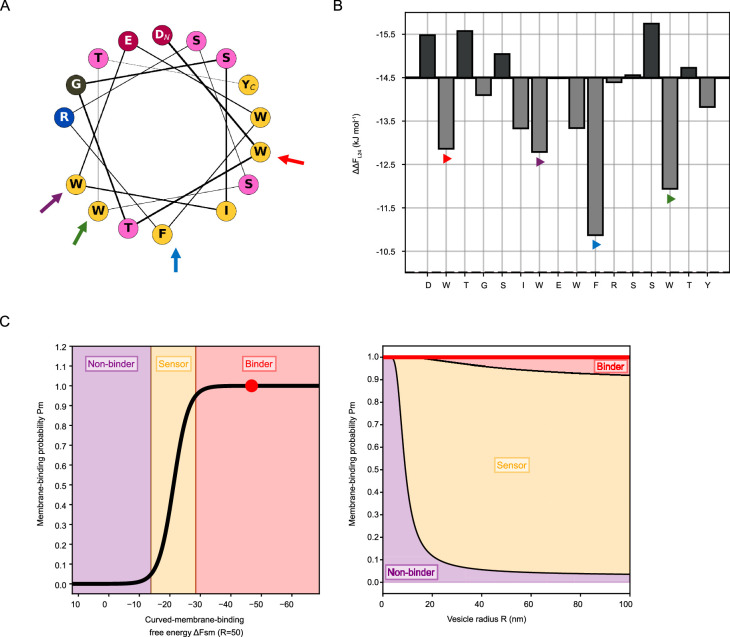
Design and expression of CLCC1 mutants. A) Helical wheel representation of CLCC1 AH-2 (residues 259-274). Arrows indicate key hydrophobic residues predicted to promote membrane binding. B) PMIpred graph of the predicted curvature-sensing free energy, extrapolated to 24-residue length. Arrows indicate key hydrophobic residues predicted to promote membrane binding. C) PMIpred prediction of CLCC1 AH-2 membrane-binding probability.

**Extended Data Fig. 9:**
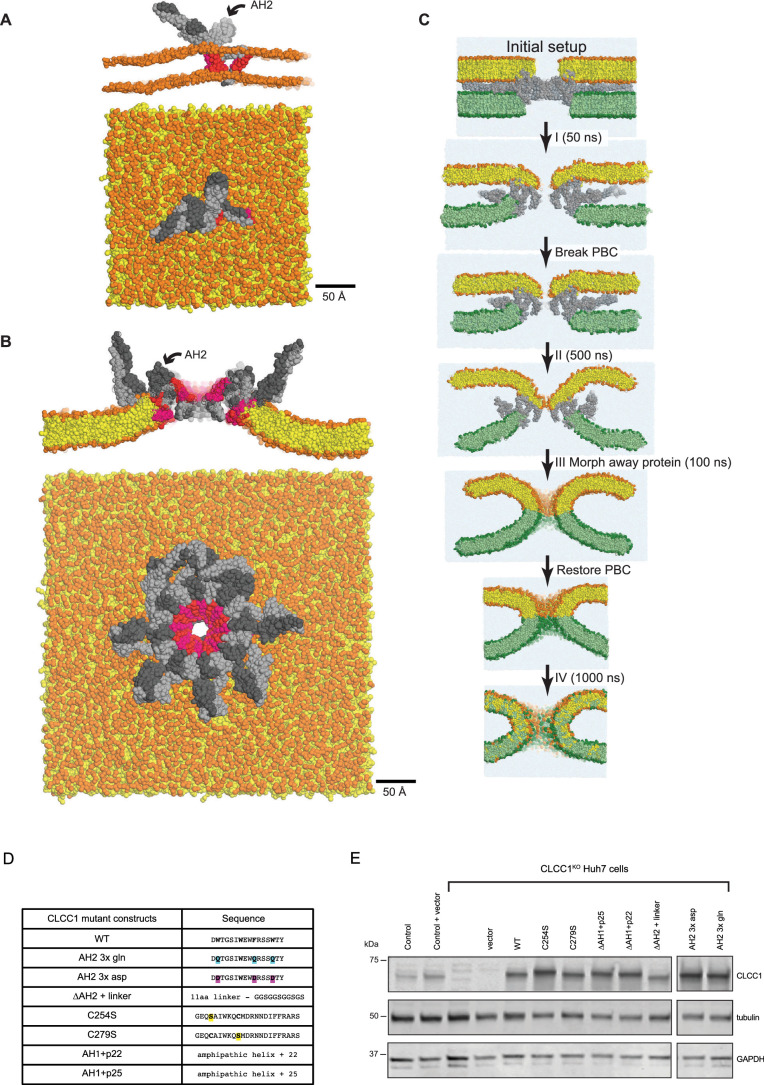
CG MD simulations of CLCC1 oligomers and expression of CLCC1 mutants. A) Side- and top view of the final CG MD simulation frame of a CLCC1 dimer initially embedded in a homogenous DOPC membrane bilayer (Supplementary Table 4b). B) Side- and top view of the final CG MD simulation frame of a CLCC1 16-mer initially embedded in a homogenous DOPC membrane bilayer (Supplementary Table 4b). In B,C: Light/dark grey: alternating protein monomers; Red/Magenta: TMHs 182-233 and 331-351 in alternating subunits. Yellow/orange: DOPC lipids (tail atoms are hidden in B for clarity). C) Simulated step-wise process of a CLCC1 16-mer promoting the fusion of two juxtaposed DOPC membranes in CG MD simulation. Yellow: upper leaflet lipids; green: lower leaflet lipids; grey: protein; blue: water/ions. I-IV correspond to Supplementary Table 4c. D) CLCC1 mutants, including (i) substitution of key hydrophobic residues in amphipathic helix AH-2 to ala, gln, and asp, (ii) swap of the amphipathic helix AH-2 for a linker, (iii) substitution of cysteine 254 and 279 for serine, and (iv) extension of the juxtamembrane amphipathic helix AH-1. p22 indicates the addition of 2 repeats of residues 297-307, inserted after F307, and p25 indicates the addition of residues 298-323, inserted after L326. E) Western blot of CLCC^KO^ cells expressing the indicated CLCC1 mutant constructs.

Structural analysis of the homologous domain of CLCC1, as well as Brl1 and Brr6, using ColabFold predicts the formation of a large oligomeric ring structure (Supplementary Fig. 12a,b and Supplementary Videos 3–8). To provide insights into the interactions of CLCC1 with membranes, we conducted coarse-grained molecular dynamics simulations. We simulated the predicted structures of the CLCC1 dimer, an oligomer of the homologous domain, and oligomers that include the CLCC1 N-terminus, starting from a system containing randomly placed 1,2-dioleoyl-*sn*-glycero-3-phosphocholine (DOPC) lipids (Supplementary Table 4a). Protein–lipid interaction frequencies were calculated from these self-assembly simulations, yielding a lipid contact map that confirmed expected lipid interactions with predicted transmembrane domains, but also revealed two spatially separated hydrophobic patches in the context of the full complex (Fig. 5f). Notably, one of the two patches creates a hydrophobic face on the CLCC1 oligomer formed by a short amphipathic helix (AH2) in the lumenal knuckle region (Fig. 5f and Extended Data Fig. 8a). Amphipathic helices are known to mediate membrane insertion, particularly into regions of phospholipid packing defects (for example, the monolayer of LDs^34,35^) and AH2 in CLCC1 is predicted by PMIpred^36^ to be capable of binding membranes (Extended Data Fig. 8b,c). The angular arrangement of the transmembrane domains of CLCC1 induces substantial membrane bending (Extended Data Fig. 9a,b and Supplementary Table 4b). All of the transmembrane regions can be accommodated with a bent membrane for the isolated dimer (Extended Data Fig. 9a). However, for the full complex, the hydrophobic patch at the lumenal side of the oligomer remained solvent exposed (Extended Data Fig. 9b). We added a second bilayer to the system to examine whether this could resolve this high energy state. Indeed, the CLCC1 oligomer juxtaposed with two flat membranes resulted in the formation of a stable fusion intermediate and a pore structure within 100 ns (Extended Data Fig. 9c and Supplementary Table 4c), satisfying all the protein–lipid interactions predicted in the self-assembly simulations. To mimic complex dissociation, we gradually morphed away the CLCC1 oligomer from this intermediate structure, which resulted in complete membrane fusion and lipid exchange between the bilayers (Extended Data Fig. 9c).

Our molecular dynamics simulations predict that two key features of CLCC1 promote membrane fusion, the lumenal AH2 insertion into adjacent membranes and the oligomeric structure remodelling of the local membrane. To evaluate the functional importance of the AH2 and the oligomeric structure of CLCC1, we performed rescue experiments in *CLCC1*-KO Huh7 cells by expressing a series of CLCC1 mutants that disrupt the hydrophobic face of AH2 or are predicted to disrupt CLCC1 oligomerization (Extended Data Fig. 9d). All mutants were expressed at similar levels to the wild-type CLCC1 (Extended Data Fig. 9e).

The expression of wild-type CLCC1 fully restored the formation of PLIN2-positive LDs and suppressed the accumulation of nuclear membrane blebs marked by MLF2–GFP (Fig. 5g and Supplementary Fig. 13). By contrast, none of the AH2 mutant constructs or the oligomerization mutants rescued the nuclear blebs (Fig. 5g and Supplementary Fig. 13). The deletion of AH2 and the glutamine mutant completely blocked the ability of CLCC1 to promote PLIN2-positive LDs, whereas the aspartate mutant had a more modest effect (Supplementary Fig. 13). These findings indicate that oligomerization and the lumenal AH2 domain are important for the cellular functions of CLCC1. We also tested mutants of the conserved cysteines (C254S and C279S) that are predicted to form an intramolecular disulfide bridge. These mutants rescued the LD phenotype and exhibited a heterogenous effect on nuclear herniations (Fig. 5g and Supplementary Fig. 13), suggesting that the intramolecular disulfide may be partially required for the role of CLCC1 in NPC assembly but is dispensable for LD regulation.

Together, our structural modelling, molecular dynamics simulations and mutagenesis studies support a model in which CLCC1 dimers assemble into an oligomer such that its TMH and lumenal AH2 regions are reconfigured to drive membrane bending and intermembrane association. The simulations further suggest that complete membrane fusion is likely to require the subsequent disassembly of the oligomeric CLCC1 complex.

## Discussion

**Extended Data Fig. 10:**
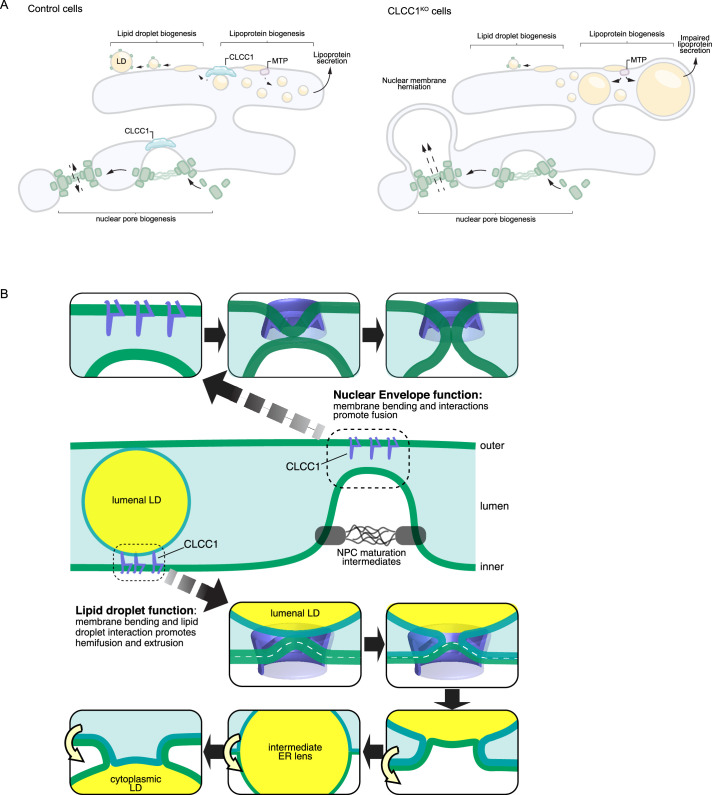
Models of CLCC1 actions at the nuclear envelope and in the ER. A) Model depicting the cellular functions of CLCC1 and the impact of CLCC1 depletion. CLCC1 is an ER and NE multi-pass transmembrane protein that promotes NPC assembly and is required for the fusion of the inner and outer NE membranes. In the absence of CLCC1, NPC assembly is impaired and large nuclear membrane herniations (i.e., blebs) accumulate. CLCC1 also regulates neutral lipid flux between cytoplasmic LDs and lumenal lipoproteins. CLCC1^KO^ cells exhibit reduced neutral lipid channeling into cytoplasmic LDs and instead neutral lipids are channeled into enlarged MTP-dependent lipoproteins that build up in the ER lumen and fail to be properly secreted. B) Our findings provide strong evidence for a role of CLCC1 in NPC assembly and ER neutral lipid channeling in hepatocytes. Similar to Brl1 and Brr6 in yeast, we propose that CLCC1 acts at the membrane fusion step of NPC assembly, which is essential for the insertion of nuclear pores during interphase. Following the insertion of nuclear pore subunits into the inner nuclear membrane, CLCC1 may be recruited to form a homo-oligomeric rings that bend the membrane towards the perinuclear space and inserts a lumenal amphipathic helix into the adjacent membrane to promote fusion. Full fusion may require disassembly of the CLCC1 oligomer. Oligomeric rings formed by Brl1 and its homologs would fulfil the same functions in yeast and organisms in all five eukaryote supergroups^57^. Within the ER, similar structural features of CLCC1 or CLCC1 homo-oligomers promote correct ER neutral lipid flux. In the absence of CLCC1 there is an aberrant increase in neutral lipid flux towards the ER lumen. One possible model is that CLCC1 reduces lumenal LD accumulation by mediating membrane fusion between the inner leaflet of the ER and a lumenal LD. This fusion would form a membrane bridge, allowing neutral lipids to migrate from the lumenal LD into the ER membrane, and emerge in cytosolic LDs via Ostwald ripening. ER scramblases such as TMEM41B (yellow arrows) could act at this step to ensure that there are sufficient phospholipids for cytosolic emergence. This could represent an LD salvage pathway to provide a homeostatic balance for maintenance of correct amounts of lumenal and cytosolic LDs.

In the current study, we establish CLCC1 as a membrane-remodelling factor with dual roles: an evolutionarily conserved function in NPC assembly and a hepatocyte-specific role in neutral lipid partitioning between LDs and lipoproteins (Extended Data Fig. 10a,b).

Cells that undergo open mitosis form NPCs through two mechanisms, either by forming NPCs on membranes associating with chromatin prior to the reformation of a sealed nuclear envelope or through inside-out insertion of the NPC into the intact nuclear envelope during interphase^27^. During inside-out NPC insertion, the inner and outer nuclear membranes fuse through a poorly understood process, perforating the nuclear envelope and enabling the completion of a mature NPC^27^. The identification of Brl1, Brr6 and their binding partner Apq12 as nuclear envelope assembly factors in yeast was an important breakthrough^21,23–27^. However, traditional sequence-based searches (PSI-BLAST, HMMER) were unable to identify a metazoan homologue of these proteins. Our data identify CLCC1 as the structural and functional human homologue of yeast Brl1 and Brr6, demonstrating a conserved role in NPC assembly (Extended Data Fig. 10).

CLCC1 and its yeast homologues share a conserved transmembrane–amphipathic helix–transmembrane architecture, which we refer to as the membrane fusion h-shaped (MFH) domain (see Supplementary Information for additional details and discussion). Structural predictions suggest that MFH proteins form oligomers, with the juxtamembrane amphipathic helix allowing them to reside in curved membranes. Our findings support a model in which CLCC1 dimers assemble into ring-shaped oligomers that bend bilayers and drive the formation of fusion intermediates, potentially involving the insertion of AH2 into the juxtaposed membrane. CLCC1 is the first example, to our knowledge, of a membrane fusogen within the lumen of the secretory pathway. Although these predictions highlight how CLCC1 could couple membrane remodelling to NPC assembly and lipid flux, important questions remain, including the stoichiometry of the oligomer, the possibility of *cis* versus *trans* interactions, and the need for experimental structural validation beyond AlphaFold models.

Our data also implicate CLCC1 in the control of hepatocellular neutral lipid flux and lipoprotein biogenesis (Extended Data Fig. 10). One possibility is that CLCC1 provides a lipid salvage pathway using a similar membrane fusogenic function as in NPC assembly, but with hemifusion of the ER membrane to the single leaflet of lumenal LDs to allow channelling of the neutral lipids into cytosolic LDs through Ostwald ripening (Extended Data Fig. 10b, bottom). Given the similar phenotypes, it is tempting to speculate that torsinA, CLCC1 and TMEM41B function together, perhaps with CLCC1 oligomers as substrates of torsinA and ER scramblases influencing nuclear envelope membrane curvature or cytosolic LD emergence. Indeed, CLCC1 is proposed to act as a positive regulator of TMEM41B^19^. Finally, recent studies indicate that CLCC1 has additional roles that require nuclear envelope membrane fusion, such as herpesvirus^37^ and large ribonucleoprotein^38^ nuclear egress. Although many questions remain for future studies, our findings represent an important advance by identifying a role for CLCC1 in hepatic neutral lipid flux and NPC assembly.

## Methods

### Cell lines and culture conditions

Huh7, U-2 OS, HEK293T and LX-2 cells were cultured in DMEM containing 4.5 g l^−1^ glucose and l-glutamine (Corning, 10-017-CM), supplemented with either 10% FBS (Thermo Fisher Scientific and Gemini Bio Products) for Huh7, U-2 OS and HEK293T or 2% FBS for LX-2, penicillin and streptomycin. HepG2 and 786-O cells were cultured in RPMI 1640 medium (Gibco, 11875093) containing l-glutamine, supplemented with 10% FBS, penicillin and streptomycin. All cells were maintained at 37 °C and 5% CO_2_. All cell lines were tested every 6 months for mycoplasma contamination.

Generation of endogenously labelled PLIN2–GFP Huh7, HepG2, U-2 OS and 786-O reporter cells was as described^8^.

### Plasmids and cloning

All knockout cell lines (in Huh7, HepG2, U-2 OS, LX-2 and 786-O cells) were generated using the pMCB320 plasmid, a gift from M. Bassik (Addgene 89359). Guide sequences for *CLCC1*, *TMEM41B*, *VMP1*, *CES1* (TGH) and safe targets (sgSAFE 5784) (Supplementary Table 2) were selected from the Bassik Human CRISPR Knockout Library (Addgene plasmids 101926, 101927, 101928, 101929, 101930, 101931, 101932, 101933 and 101934). Guide sequences were cloned into pMCB320 using the restriction enzymes BstXI and BlpI.

For exogenous protein expression, CLCC1 (DNASU, HsCD00951632), 1× Flag (DYKDDDDK)-tagged CLCC1, TMEM41B (DNASU, HsCD00829148), and VMP1 (DNASU, HsCD00080545) were cloned using Gibson assembly (New England Biolabs, E2611S) and the Gateway system (Thermo Fisher, 11791020) into a pLenti-CMV-Hygro vector (Addgene 17454). A pLenti-CMV-Hygro vector containing GFP with a tandem nuclear localization signal (NLS) (PKKKRKV) and nuclear export signal (NES) (LALKLAGLDI) sequence was ordered from GenScript. For the CLCC1-Brl1 chimera, the MFH domain (G362–Y434) of Brl1 (a gift from E. Ünal) was swapped with the MFH domain (D276–H362) of CLCC1 (DNASU, HsCD00951632) using Gibson assembly and was cloned into the pLenti-CMV-Hygro vector (Addgene plasmid 17454). Wild-type and mutant CLCC1 constructs used for structure–function analyses were synthesized by Twist Bioscience and cloned into the pLenti-CMV-Hygro vector (Addgene 17454). The mutant sequences were as follows: C254S (C254S), C279S (C279S), AH1 + p22 (TKALAVTFTTF-TKALAVTFTTF inserted after V307), AH1 + p25 (KALAVTFTTFVTEPLKHIGKGTGEF inserted after L326), ∆AH2 + linker (GGSGGSGGSGS between K257 and K275), AH2 3× Asp (W260D/F268D/W272D) and AH2 3× Gln (W260Q/F268Q/W272Q). Each mutant construct included two silent mutations, one within the PAM sequence and another within the CLCC1 sgRNA target site, in addition to the mutation of interest. These silent mutations (numbered according to the CLCC1 wild-type sequence) were: PAM mutation (1011A > C) and sgRNA target site mutation (1017G > T). This design allowed stable expression of CLCC1 variants in Cas9-expressing *CLCC1*-KO Huh7 cells, preventing re-editing by the expressed sgRNA. Lentiviral particles were produced as described above and used to transduce *CLCC1*-KO Huh7 cells. Transduced cells were selected in hygromycin-containing medium (200 μg ml^−1^) until all uninfected control cells were eliminated.

### Generation of CRISPR–Cas9 genome-edited cell lines

To generate lentiviral particles, lentiCas9-Blast plasmid (Addgene 52962) was co-transfected with third-generation lentiviral packaging plasmids (pMDLg/pRRE, pRSV-Rev, and pMD2.G) into HEK293T cells. Lentiviral medium was collected 72 h after transfection, passed through a 40-µm filter, and then used to infect Huh7 (wild type, PLIN2–GFP), HepG2 (wild type, PLIN2–GFP) and U-2 OS (wild type, PLIN2–GFP) cells. Cells were selected in medium containing blasticidin (4 μg ml^−1^ in Huh7 and U-2 OS; 6 μg ml^−1^ in HepG2) for 5 days. Active Cas9 expression was validated by flow cytometry analysis following infection with a self-cleaving mCherry plasmid (pMCB320 containing mCherry and an sgRNA targeting the mCherry gene).

Lentiviral particles with sgRNA-containing pMCB320 plasmids were generated as described above and used to infect cells stably expressing Cas9. After 72 h of growth, infected cells were selected in medium containing puromycin (2 μg ml^−1^ in Huh7 and HepG2 cells; 1 μg ml^−1^ in U-2 OS cells) until over 90% cells were mCherry positive and all uninfected control cells were dead. Huh7 *CLCC1*-KO and *TMEM41B*-KO clones (in wild type and PLIN2–GFP backgrounds) were isolated using serial dilutions. Knockout efficiencies were confirmed via immunoblotting.

### Genome-wide Huh7 CRISPR–Cas9 screens

All CRISPR–Cas9 screens reported here were performed as described previously^8,39^. Genome-wide CRISPR–Cas9 screens were performed using the Bassik Human CRISPR Knockout Library. The library consists of 9 sublibraries, comprising a total of 225,171 elements, including 212,821 sgRNAs targeting 20,549 genes (∼10 sgRNAs per gene) and 12,350 negative-control sgRNAs. Lentiviral particles containing each sublibrary were generated as described above. Huh7 cells stably expressing Cas9 were transduced with lentiviral packaged sublibraries (one sublibrary at a time) in 8 μg ml^−1^ polybrene. After 72 h of growth, infected cells were selected in medium containing 2 μg ml^−1^ puromycin until over 90% of cells were mCherry positive (via flow cytometry). Cells were then recovered for 3–5 days in medium lacking puromycin and frozen in liquid nitrogen.

For the screen, library infected cells were thawed (one sublibrary at a time) and maintained at 1,000× coverage (1,000 cells per library element) in 500 cm^2^ plates (about 2 × 10^7^ cells per plate). Library-infected cells were passaged once before sorting. On the day of the sort, cells were dissociated using 0.25% Trypsin-EDTA (Gibco), collected by centrifugation at 300*g* for 3 min, stained with 1 µg µl^−1^ BODIPY 493/503 (Thermo Fisher Scientific, D3922) in DPBS on ice for 30 min, then washed once with DPBS. Cells were resuspended in phenol red-free medium (HyClone, 16777-406) supplemented with 3% FBS and 1% fatty acid-free BSA and kept on ice until FACS.

Cells were sorted on a BD FACS Aria Fusion equipped with 4 Lasers (488 nm, 405 nm, 561 nm and 640 nm). sgRNA-expressing, mCherry^+^ cells were gated into the brightest 30% and dimmest 30% by the 488 nm laser. Cells were sorted into 15 ml Falcon tubes containing DMEM with 4.5 g l^−1^ glucose and l-glutamine supplemented with 10% FBS. For each sort, 1,000 cells were collected (500 in each gate). Sorted cells were collected and sequenced as previously described^39^. Results from the genome-wide screen are available in Supplementary Table 1.

### LD and metabolism library CRISPR–Cas9 screens

The custom human VLDM library contains 10,550 elements, with 8,550 sgRNAs targeting 857 genes (∼10 sgRNAs per gene) and 2,000 negative-control sgRNAs. Guide sequences were from the Bassik Human CRISPR Knockout Library, and the library construction protocol and cell line generation were previously described.

For each screen, cells were thawed and expanded at >1,000× coverage. For all screens, cells were seeded into 500 cm^2^ plates at 1,000-fold library coverage. For the Huh7 metabolic state-dependent screens, cells were treated the following day with: (1) no treatment; (2) 1 μg ml^−1^ triacsin C for 24 h; (3) 100 μM oleate–BSA complex for 24 h; (4) HBSS (Gibco, 14025092) for 24 h; (5) 0.2% FBS-containing DMEM (serum starve) for 48 h; (6) glucose-free DMEM (Gibco, 11966025) for 24 h; (7) 50 μM palmitic acid for 24 h; (8) 5 μM arachidonic acid for 24 h; (9) 5 μg ml^−1^ tunicamycin for 24 h; (10) 500 ng ml^−1^ LPS for 24 h; or (11) MASH stress mix (10 mM glucose, 5 mM fructose, 400 µM oleic acid, 200 µM palmitic acid, 100 ng ml^−1^ LPS and 30 ng ml^−1^ TNF) for 16 h. Cells were screened by FACS as described above. Results from each metabolic state screen are available in Supplementary Table 1.

### CRISPR screen data analysis

Sequence reads were aligned to the sgRNA reference library using Bowtie 2 v.2.3.4.3 software. For each gene, a gene effect and score (likely maximum effect size and score) and *P* values were calculated using the Cas9 high-throughput maximum likelihood estimator (castle v.1.0) statistical framework as previously described.

Morpheus (https://software.broadinstitute.org/morpheus/) was used to perform unbiased gene clustering on metabolic state screens. Genes were ranked according to casTLE score and complete Euclidean linkages. Functional interactions and protein-protein interactions for high confidence candidate regulators were identified using the STRING database using STRING v.12.0^40^.

### General animal care

All procedures involving mice were approved by the UC Berkeley IACUC (protocol AUP-2022-02-15079-1, approved May 2022) and conducted in accordance with the NIH Guide for the Care and Use of Laboratory Animals, PHS Policy, and the Animal Welfare Act. Mice were maintained up to 12 weeks of age on a 12 h light:12 h dark cycle at ambient temperature (23 °C) and 30–70% relative humidity in the UC Berkeley pathogen-free barrier facility with free access to water and standard laboratory chow diet (LabDiet, 5053). We used *Clcc1*^*flox/flox*^ in the C57BL/6 J genetic background (stock no. 000664). Experimentation was performed between 8 and 12 weeks of age. In mouse experiments, all measurements were included in the analysis. Mice were randomly allocated to groups; the only criteria were sex and age as explained above. The sample size and number of replicates for this study were chosen based on previous experiments performed in our laboratory and others. No specific power analysis was used to estimate sample size. Imaging studies could not be done blinded owing to the evident intrinsic features of the datasets. In vivo studies could not be blinded owing to the viral injection protocol. Experimental and control samples were processed together using the same conditions.

### Floxed *Clcc1* mouse generation

*Clcc1* floxed mice (generated by the Knockout Mouse Project) (C57BL/6N-Atm1Brd Clcc1tm1a(KOMP)Mbp/JMmucd), where exon 7 is floxed, were obtained from the University of California Davis Mouse Biology Program. The neomycin selection cassette and *lacZ* reporter were removed by breeding to CAG-Flpo (C57BL/6N-Tg(CAG-Flpo)1Afst/Mmcd). Mice were then bred for at least four generations to C57BL/6 J mice, removing the CAG-Flpo. Genotyping for the floxed allele from genomic DNA was performed with the following PCR primers: TCATGACATGAACCATATGTGAATTCC and CACCATGCCTGGCTACAAATGC.

### Adeno-associated virus mediated deletion of *Clcc1*

To deplete CLCC1, 8-week-old homozygous *Clcc1-*floxed mice were injected with either AAV8-TBG-Cre (Addgene 107787, a gift from J. M. Wilson) or AAV8-TBG-GFP (Addgene 105535, a gift from J. M. Wilson), via tail vein at a titre of 1.0 × 10^11^ genome copies per mouse. Mice were euthanized by CO_2_ four weeks after injection and livers were photographed. More than four mice per group were analysed per experiment. The liver was weighed and divided into pieces, which were flash frozen in liquid nitrogen, transferred on dry ice, and stored at −80 °C.

### Mouse plasma collection and analysis

Blood was collected via submandibular vein puncture and centrifuged at 2,000*g* in microtainer SST tubes (BD, 365967) for 15 min at 4 °C to isolate plasma. Plasma was flash frozen in liquid nitrogen and stored at −80 °C. AST, ALT, and lipoprotein levels were analysed via Clinical Analyzer (Merck & Co). TAGs were quantified by a luciferase-based assay (Promega, J3160).

### FPLC separation of mouse plasma

Size-exclusion chromatography was performed using an AKTA FPLC (Amersham Pharmacia Biotech). Equivalent volumes of plasma from each group of mice were pooled, totalling 400 μl (females) or 300 μl of plasma (males). Plasma was diluted in PBS so total sample volume equalled 1,000 μl and was applied to a Superose 6 followed in tandem with a Superdex 200 column and separated into lipoprotein classes in 10 mM PBS, pH 7.4, containing 0.02% sodium azide and collected into 48× 0.5 ml fractions. Fractions were then analysed for cholesterol (C7510) and TAGs (T7532) with indicated colorimetric kits (Pointe Scientific).

### Flow cytometry

Cells were washed twice in DPBS, dissociated using TrypLE Express (Gibco, 12605010), collected by centrifugation at 500*g* for 5 min, and stained with 1 µg µl^−1^ BODIPY 493/503 or 200 µM monodansylpentane (MDH, Abcepta, SM1000b) in DPBS on ice for 30 min.

For all flow cytometry assays, fluorescence was analysed using an LSR Fortessa (BD Biosciences) using BD FACS v.6.2 (BD Biosciences). The following filter sets were used: FITC (GFP, BODIPY 493/503), Pacific Blue (BFP, MDH) and Texas-Red (mCherry). FlowJo v.10 software (BD Biosciences) was used to quantify fluorescence and generate representative histograms. Example gating strategy is shown in Supplementary Fig. 14.

### Immunoblotting

For intracellular proteins from tissue culture, cells were lysed in 1% SDS and sonicated at 15% amplitude for 15 s. For secreted proteins from tissue culture, cells were incubated for 24 h in FBS-free DMEM, proteins were precipitated from the medium with acetone, and the pellet was resuspended in 100 μl 1% SDS. Mouse plasma samples were diluted 1:25 in 1% SDS. Animal tissues were homogenized in NP-40 lysis buffer (1% NP-40, 50 mM Tris-HCl, 5 mM EDTA, 2 mM EGTA, 30 mM NaF, 10 mM sodium pyrophosphate and 40 mM B glycerolphosphate) with an immersion homogenizer for 15 s. Protein concentrations were determined and normalized using a BCA protein assay (Thermo Fisher Scientific, 23225). Equal amounts of protein were combined with Laemmli buffer, boiled for 10 min at 95 °C, separated on 4–20% polyacrylamide gradient gels (Bio-Rad Laboratories) and transferred onto 0.45-mm nitrocellulose membranes at 25 V for 3 min (Bio-Rad Laboratories). Membranes were incubated in 5% nonfat milk in PBS with 0.1% Tween-20 (PBST) for 30 min to reduce nonspecific antibody binding. Membranes were then incubated overnight at 4 °C in 5% BSA in PBST containing the following primary antibodies at 1:1,000 dilution: rabbit anti-CLCC1 (Thermo Fisher Scientific, HPA009087), rabbit anti-PLIN2 (Abcepta, AP5118c), rabbit anti-albumin (Proteintech, 16475-1-AP), mouse anti-MTP (Santa Cruz, sc-515742), goat anti-CES1/TGH (R&D Systems, AF4920SP), rabbit anti-BiP (Cell Signaling, C50B12), rabbit anti-TMEM41B (Proteintech, 29270-1-AP), rabbit anti-VMP1 (Cell Signaling, D1Y3E), mouse anti-lamin A/C (Cell Signaling, 4777), rabbit anti-calnexin (Cell Signaling, C5C9), mouse anti-actin (Cell Signaling, 4970), and rabbit anti-GAPDH (Cell Signaling, 2118). Membranes were incubated for at least 1 h in IRDYE secondary antibodies (LI-COR, 926-68074, 926-68070, 926-32211) at a 1:10,000 dilution in PBST containing 5% nonfat milk. Immunoblots were visualized on a LI-COR imager (LI-COR Biosciences) running Odyssey v.3.0, and Fiji/ImageJ v.1.53e (NIH) was used for quantification of protein levels.

For immunoblotting apoB, FPLC fractions were diluted with 2× Laemmli buffer and run as described above. Gels were then transferred to 0.45-μm nitrocellulose membranes at 350 mA for 1.5 h. Membranes were blocked in 5% nonfat milk in PBST overnight at 4 °C, incubated with 1:1,000 anti-apoB antibody (Abcam, ab20737) for 2 h at room temperature and subsequently incubated with 1:10,000 goat anti-rabbit secondary antibody (LI-COR, 926-68074) for 1 h at room temperature.

### Nuclei isolation

Nuclei were isolated using the Nuclei EZ Prep Kit (Sigma, NUC101). Samples were diluted 1:1 with 1% SDS and were immunoblotted for purity with lamin A/C, calnexin and CLCC1 as described above.

### Fluorescence microscopy

For widefield microscopy of live cells, Huh7, HepG2, U-2 OS and 786-O cells were grown in 4-well or 8-well Nunc LabTek II Chambered Coverglass (Borosilicate Glass 1.5; Thermo Fisher Scientific, 155360) coated with poly-l-lysine. LDs were stained with 1 μM BODIPY 493/503 for 30 min or 500 nM Lipi-Blue for 30 min, nuclei were stained with 5 μg ml^−1^ Hoechst 33342 (Thermo Fisher Scientific, 62249) for 30 min, lysosomes were stained with 75 nM Lysotracker DND-22 (Thermo Fisher Scientific, L7525) for 30 min, and mitochondria were stained with 500 nM Mitotracker Green (Thermo Fisher Scientific, M7514) for 30 min. For imaging the ER, cells were transiently transfected with BFP-KDEL (Addgene 49150) and imaged 48 h later. For imaging of nuclear blebs, cells were transiently transfected with MLF2-GFP-Flag-pCDNA3.1 (a gift from C. Shlieker) and imaged 48 h later. Prior to imaging, cells were washed twice with DPBS and imaged in fresh phenol red-free medium supplemented with 10% FBS. Live cells were imaged using a Zeiss Axio Observer 7 fitted with a 63× oil objective using DAPI, GFP, Cy-3 and Cy-5 filters. Cells were imaged at 37 °C with 5% CO_2_. *z*-stacks of 0.2-μm thickness were acquired using ZEISS ZEN v.3.2 (ZEISS Microscopy) software.

To evaluate the endogenous localization of CLCC1 and ER, Sec61β–GFP was overexpressed in control (expressing sgSAFE) and *CLCC1*-KO Huh7 cells. Twenty-four hours after transfection, cells were fixed with 4% paraformaldehyde for 10 min at room temperature and washed with 1× PBS three times, followed by a 20 min permeabilization using 0.2% Triton X-100 plus 3% BSA at room temperature. Cells were washed three times with 1× PBS before staining. Endogenous CLCC1 and Lamin A/C proteins were stained with rabbit anti-CLCC1 (Thermo Fisher Scientific, HPA009087) and mouse anti-Lamin A/C (Cell Signaling, 4777) antibodies overnight at +4 °C, at concentrations of 1:50 and 1:200, respectively, in 1× PBS plus 3% BSA. Cells were then washed three times with 1× PBS and stained using fluorescent secondary antibodies diluted at 1:1,000 in 1× PBS plus 3% BSA for 2 h in the dark at room temperature. Nuclear staining was performed using HCS NuclearMask Stains (Invitrogen, H10325,), 1:2,000 in 1× PBS at room temperature for 10 min. For the analysis of the effects of CLCC1 mutants on LD content, PLIN2 localization, and MLF2–GFP nuclear blebs, Huh7 cells stably expressing each CLCC1 construct were transfected with MLF2–GFP in the Flag-pCDNA3.1 vector. After 48 h, cells were fixed, processed as described above, and stained for LDs using Lipi-Blue and for PLIN2 (Proteintech, 15294-1-AP).

For widefield and Lattice Structural Illumination Microscopy (SIM) of fixed cells, Huh7 cells were grown either in 12-well plates on glass coverslips coated with poly-l-lysine or in 35 mm dishes (Invitrogen, C10046). Cells were washed three times with DPBS, fixed for 15 min in 4% (w/v) PFA in DPBS and washed three times again with DPBS. Cells were permeabilized for 15 min with 1% BSA in DPBS containing 0.1% Triton X-100 when staining for ER, Golgi, or nuclear proteins or 0.01% digitonin when staining for LD proteins and then washed three times with DPBS. Cells were then incubated for 2 h in the dark at room temperature with the following antibodies diluted 1:1,000 in 1% BSA in DPBS: rabbit anti-PLIN2 (Abcepta, AP5118c), GM130 (Cell Signaling, 12480), rabbit anti-CLCC1 (Thermo Fisher Scientific, HPA009087), KDEL (Abcam, ab176333), mouse anti-lamin A/C (Cell Signaling, 4777), goat anti-ApoB (Rockland, AB742) or Mab414 (Abcam, ab24609). Cells were washed three times with DPBS before staining for LDs with 1 μM BODIPY 493/503 for 30 min or 500 nM Lipi-Blue for 30 min, nuclei with 1 µg ml^−1^ DAPI, and fluorescent secondary antibodies (Thermo Fisher Scientific, A21202, A-21109) diluted at 1:1,000 in 1% BSA in DPBS for 30 min in the dark. Cells were washed three times with DPBS and coverslips were mounted on 1 mm glass slides using Fluoromount-G (SouthernBiotech, 0100-01). Widefield fluorescence images were acquired using a Zeiss Axio Observer 7 as above and Lattice-SIM images were acquired on a Zeiss Elyra7 superresolution fluorescence microscope, equipped with dual sCMOS PCO Edge 4.2 cameras for simultaneous two channel acquisition, with a 63×/1.4 oil objective. For each focal plane 13 phase images were acquired. Lattice-SIM reconstruction was performed with the SIM processing Tool of the ZEN 3.0 SR Black v.16 (ZEISS Microscopy) software.

For live-cell high-throughput confocal microscopy, Huh7 cells were grown in 24-well glass bottom plates (170 μm coverglass bottom; Eppendorf, 0030741021; Cellvis, P24-1.5H-N). LDs were stained with 1 μM BODIPY 493/503 and nuclei were stained with 5 μg ml^−1^ Hoechst 33342 in DPBS for 30 min. Prior to imaging, cells were washed twice with DPBS and imaged in fresh phenol red-free medium supplemented with 10% FBS. Live cells were imaged using an Opera Phenix Plus High-Content Screening System (Perkin Elmer) confocal microscope equipped with a 40× water immersion objective using DAPI and GFP filters. Cells were imaged at 37 °C with 5% CO_2_. *z*-stacks of 0.3-μm slices were acquired.

### Fluorescence microscopy analysis

Widefield and superresolution images were merged and brightness and contrast adjusted using Fiji/ImageJ. PLIN2^+^ and apoB^+^ LDs were quantified by hand. Nuclear pores were quantified by randomly selecting nuclei using the DAPI channel, outlining the nuclei manually, dividing the nucleus into 1 µm^2^ segments, and quantifying the number of foci per nucleus using the ‘analyzer particles’ function with a noise tolerance of 15. Foci were averaged per nucleus and graphed in Prism v.9 (Graphpad).

LDs and nuclear blebs (MLF2–GFP foci) were quantified from confocal images by creating custom analysis sequences using Harmony High Content Image Analysis Software v.4.9 (Perkin Elmer). For each field, maximum projection *z*-stacks were processed with advanced flatfield correction. Nuclei and cytoplasm were defined using the DAPI and GFP channels, respectively, and border cells were automatically excluded from analyses. LDs and nuclear blebs were defined using the ‘find spots’ building block (GFP channel), thresholding for size, intensity, and roundness. For each cell, LD number and area or number of nuclear blebs were quantified. Nucleus size and GFP intensity were also quantified using these methods. Quantification data were graphed and analysed in Prism 9 (GraphPad). The quantification of nuclear blebs (MLF2–GFP foci) and PLIN2 signal around the LDs in Huh7 cells expressing the various CLCC1 mutants (Supplementary Fig. 13) were performed following the pipeline described in Supplementary Fig. 13k, using an ImageJ macro (https://github.com/gparlakgul).

### Transmission electron microscopy

For cell lines, Huh7 and U-2 OS cells were grown on 3 cm LabTek dishes and fixed in 2% paraformaldehyde and 2% glutaraldehyde in PBS. Samples were stained with 1% osmium tetroxide and 1.5% potassium ferrocyanide for 1 h and 1% uranyl acetate overnight. The next day, samples were washed and subsequently dehydrated in grades of ethanol (10 min each; 30%, 50%, 70%, 95% and twice for 10 min at 100%). Samples were embedded in increasing concentrations of eponate resin mixed with ethanol (30 min each; 1:2, 1:1, 2:1 and 100% acetone) followed by polymerization in 100% eponate overnight at 50 °C.

For liver tissues, mice were anaesthetized with 300 mg kg^−1^ ketamine and 30 mg kg^−1^ xylazine in PBS and perfused with 10 ml of DPBS followed by 10 ml of fixative buffer containing 4 parts of FP stock (2.5% PFA, 0.06% picric acid in 0.2 M sodium cacodylate buffer pH 7.4) and 1 part of 25% glutaraldehyde. After perfusion, small pieces (1–2 mm^3^) of liver were sliced at 300 μm thickness with a compresstome, transferred into a fresh fixative solution containing and incubated at 4 °C overnight. Samples were then washed in ice-cold 0.15 M sodium cacodylate buffer for 5 min, three times, and then incubated in 0.1 M sodium cacodylate solution containing 1% osmium tetroxide and 1.5% potassium ferrocyanide for 1 h at 4 °C. Samples were rinsed three times with water and incubated for 20 min in 1% thiocarbohydrazide and rinsed again three times for 5 min with water. Samples were incubated in 2% osmium tetroxide for 30 min and then rinsed three times for 5 min with water, followed by washing three times and incubation overnight at 4 °C in 1% uranyl acetate in maleate buffer. The next day, samples were washed and subsequently dehydrated in grades of acetone (10 min each; 50%, 70%, 90% and twice for 10 min at 100%). Samples were embedded in increasing concentrations of eponate resin mixed with acetone (30 min each; 50%, 70%, 90% and 100% acetone) followed by incubation in 100% eponate for 4 h. The samples were moved to fresh 100% eponate and polymerized at 65 °C for 24 h.

The resin-embedded sample blocks were trimmed, and 70 nm ultrathin sections were cut using a Leica UC6 ultramicrotome (Leica Microsystems) and collected onto formvar-coated slot grids. Sections were imaged to find target regions using a Tecnai 12 120 kV TEM (FEI) and data recorded using an Gatan Rio16 CMOS camera and GMS3 software (Gatan).

### FIB-SEM

Mouse livers were fixed and prepared as described above. The trimmed sample blocks were glued with silver paint (Ted Pella) onto Al stubs, and sputter coated (Pd/Au) with a Tousimis sputter coater on top of a Bio-Rad E5400 controller. FIB-SEM imaging was performed using a Zeiss Crossbeam 550 (Carl Zeiss Microsystems). The sample was tilted at 54° in order to be perpendicular to ion beam. The FIB milling and SEM imaging of the target area were set up using Atlas 5 3D tomography (Carl Zeiss Microsystems). Slices with a thickness of 10 nm were milled from the target area using the 30 kV 300pA ion beam. Energy-selective Backscattered (ESB) images were collected at 1.5 kV, 1 nA, with a dwell time of 18 ns, image pixel size of 10 nm, and tilt correction angle of 36°. The collected images were aligned with slice registration in Dragonfly 2022.2 (Comet Technologies).

### FIB-SEM data segmentation, quantification and visualization

Ground truth labels were generated by manually annotating each class (ER, mitochondria, nucleus and LDs) in five consecutive full-size images using Napari v.0.4.18. Tunable 2D-U-Net networks (DLSIA) were used to obtain rough predictions for each class^41^. These rough predictions were manually proofread and corrected in Napari. A block consisting of at least 250 × 250 × 250 voxels was used to train and fine-tune 3D-U-Net network models with Incasem^42^. Additional proofreading and manual corrections were performed in Napari. Objects, images, videos and quantifications from each class were generated using Arivis Vision 4D v.3.6.0 (ZEISS Microscopy).

Nuclear pores were manually labelled and annotated in Napari, image by image (1,142 images for wild type and 897 images for the knockout), in both datasets. After manual annotation, nuclear pore objects were reconstructed in Arivis Pro software to visualize the three-dimensional continuity and organization of the nuclear pores. To quantify the density distribution, the nuclear membrane surface was divided into ~100 patches using a Python 3.0 script (https://github.com/gparlakgul/nuclear_pore), with each patch assigned a unique pixel intensity as an identifier (Supplementary Fig. 15). These patches were imported into Arivis Pro. False nuclear surfaces (flat surfaces adjacent to the dataset borders) were excluded. Nuclear pores were assigned to the corresponding nuclear surface patch, and the number of nuclear pores per patch was calculated. The quantifications were normalized by the surface area of each nuclear membrane patch. Nuclear blebs were separated from the nuclear membrane, and the number of blebs was quantified.

### Liver histology

Liver preparation was performed as described above with 4% PFA. Liver pieces were embedded in OCT, frozen, and cryosectioned into 5-µm-thick sections. Liver sections were fixed in 4% paraformaldehyde for 20 min and stained with either haematoxylin and eosin, oil red O, Masson’s trichrome, or picrosirius red by HistoWiz. Images were analysed using PathologyMap 2.0 software.

### Primary hepatocyte isolation

Mice were anaesthetized using 300 mg kg^−1^ ketamine and 30 mg kg^−1^ xylazine in PBS. The livers were perfused with 50 ml of buffer I (11 mM glucose, 200 μM EGTA, 1.17 mM MgSO_4_ heptahydrated, 1.19 mM KH_2_PO_4_, 118 mM NaCl, 4.7 mM KCl and 25 mM NaHCO_3_, pH 7.32) through the portal vein with an osmotic pump set to the speed of ~4 ml min^−1^ until the liver turned pale. The speed was gradually increased until ~7 ml min^−1^ afterwards. When the entire buffer I had been infused, it was substituted for 50 ml of buffer II (11 mM glucose, 2.55 mM CaCl_2_, 1.17 mM MgSO_4_ heptahydrated, 1.19 mM KH_2_PO_4_, 118 mM NaCl, 4.7 mM KCl, 25 mM NaHCO_3_, BSA (fatty acid-free) 7.2 mg ml^−1^ and 0.18 mg ml^−1^ type IV collagenase (Worthington Biochemical, LS004188)), BSA and collagenase were added immediately before use. The buffers were kept at ~37 °C during the entire procedure. After perfusion, the primary hepatocytes were carefully released and sedimented at 500 rpm for 2 min, washed twice and suspended with Williams E medium supplemented with 5% CCS and 1 mM glutamine (Invitrogen, A1217601). To separate live from dead cells, the solution of hepatocytes was layered on a 30% Percoll gradient and centrifuged ~1,500 rpm for 15 min. The healthy cells were recovered at the bottom of the tube and plated in 35 mm imaging dishes for experimentation.

### BODIPY 558/568 C12 incorporation assay

Huh7 safe-targeting control and *CLCC1*-KO cells were seeded in 60-mm plates at 350 cells per plate. To determine the rate of LD biogenesis, cells were incubated in BODIPY C12-BSA complex (complete medium with 1% BSA and 1 μM BODIPY 558/568 C12 (Invitrogen, D3835)) for 0, 1, 3 or 6 h. Cells were collected by washing them twice, collecting in cold DPBS, and transferring to Eppendorf tubes (Eppendorf, 022363352). Cells were centrifuged at 500*g* for 5 min, washed in DPBS, and centrifuged again. Cell pellets were stored at −80 °C until the lipid extraction step. For the lipolysis assay (measuring loss of esterified C12), cells were incubated in BODIPY C12-BSA complex for 16 h. Cells were then washed three times with medium and incubated in fresh medium for 1 h. Cells were then treated with 1 µg ml^−1^ triacsin C for 0, 6 or 24 h. Cells were collected, and pellets stored at −80 °C as described above.

### TAG measurements by TLC

Cell pellets were thawed at room temperature and resuspended in 50 μl DPBS. Liver tissues (approximately 30 mg, 3 per mouse) were homogenized in 1 ml methanol using an immersion homogenizer for 5 min at 4 °C. Lipids were extracted by adding *tert*-butyl methyl ether (1.25 ml) and methanol (375 μl). The mixture was incubated on an orbital mixer for 1 h at room temperature. To induce phase separation, water (315 μl) was added, and the mixture was incubated on an orbital mixer for 10 min at room temperature. Samples were centrifuged at 1,000*g* for 10 min at room temperature. The upper organic phase was collected and subsequently dried in vacuo.

Dried lipid extracts were reconstituted in 30 μl (cells) or 200 μl (liver) chloroform/methanol (2:1, v/v). Lipids were then separated using HPTLC Silica gel 60 F254 plates (Sigma, 1137270001). Ten microlitres of the cell samples and 2 μl of the liver samples were spotted onto TLC plates and developed in CHCl_3_/ethanol/triethylamine/H_2_O (5:5:5:1, v/v). Plates were imaged on a ChemiDoc MP Imaging System (Bio-Rad Laboratories). Band densitometry analysis was performed using Image Lab v.6.0.1 (Bio-Rad Laboratories). The reported mean ± standard deviation was determined from three biological replicates.

### Proteomic analysis of LD proteins

Safe-targeting and *CLCC1*-KO cell lines were grown until confluent in 500 cm^2^ plates of cells were collected by scraping in DPBS, centrifuged for 10 min at 500*g*, and stored at −80 °C. Buoyant fractions containing 1% SDS were acidified to a final concentration of 15% trifluoroacetate. Samples were then cooled on ice for 30 min and centrifuged at 20,000*g* for 30 min at 4 °C. The protein pellet was washed three times with 500 μl of ice-cold acetone and centrifuged for 10 min between each wash. The protein pellet was then dried in a vacuum evaporator for 10 min. Dried, precipitated proteins were resuspended in 0.1% RapiGest with 6 μl of sequencing-grade trypsin (Promega, 0.5 μg μl^−1^) added to each sample and digested overnight at 37 °C. Trypsinized samples were quenched with a final concentration of 5% TFA. Samples were desalted using the Waters Sep-pak 1cc (50 mg) C18 cartridge.

Peptides were resuspended in 1% formic acid and 0.5 μg of peptides were separated on an Easy nLC 100 UHPLC equipped with a 15 cm nano-liquid chromatography column. Using a flow rate of 300 nl min^−1^, the linear gradient was 5% to 35% over B for 90 min, 35% to 95% over B for 5 min and 95% hold over B for 15 min (solvent A 0.1% formic acid in water, solvent B 0.1% formic acid in acetonitrile). Peptide identified and relative abundances were determined using Proteome Discoverer v.2.4 (Thermo Fisher Scientific). Results are represented as mean ± s.d. of duplicates.

### RNA sequencing

Triplicates of safe-targeting and *CLCC1*-KO cells were seeded in 6 cm plates. RNA was isolated using the Monarch Spin RNA Isolate Kit (New England Biolabs, T2110S). Sequencing results were analysed using Partek Flow (Illumina) running DESeq2 v.1.5.

### ApoB ELISA assay

Safe-targeting and *CLCC1*-KO cells were seeded in 6 cm plates and treated with 1 µg ml^−1^ DMSO or 50 nM MTPi for 72 h. Twenty-four hours before collection, cells were changed into FBS- and phenol red-free medium. Medium was collected and apoB ELISA Assay (Sigma Aldrich, RAB069) was performed according to protocol. ApoB levels were normalized to cell protein levels and results are represented as mean ± s.d. of two biological duplicates.

### ASGR luciferase assay

The ASGR reporter plasmid was generated by the laboratory of G. S. Hotamisligil as previously described^15^. Safe-targeting and *CLCC1*-KO cells were infected with lentivirus containing the ASGR construct. For the experiment, cells were changed to a fresh phenol red-free medium and incubated for 24 h with or without increasing doses of thapsigargin. 10 µl of medium was transferred to 96-well white plates (Corning, 3917) for luciferase assays following the manufacturer’s protocol. In brief, 50 µl of luciferase substrate (1 µM *Cypridina* (CLUC) or 10 mM coelenterazine (GLUC) in 100 mM tris buffer, pH 7.5) was added to the 10 µl medium and incubated in the dark for 5 min. The luminescence was read on Infinite 200 PRO plate reader using i-control software (Tecan).

### Structure predictions

Monomeric and multimeric sequences were submitted to AlphaFold2 using MMseqs2 using either the Google Colabatory^43^ or COSMIC2^44^ or were submitted to DMFold, MultiFOLD, or trRosetta^45^. The pLDDT of core homology regions as monomers and ring oligomers predicted by AlphaFold2: CLCC1 residues 209-353: monomer 79.2%, 16-mer: best 80.9%, average 80.2%; Brr6 residues 44-185: monomer 80.5%, 16-mer: best 78.1%, average 77.0%^46,47^.

### General molecular dynamics simulation details

Simulations were performed with Gromacs (v.2023.3^40^) using the molecular dynamics integrator (unless stated otherwise), and the Martini 3 force field (v.3.0.0^48^) at a 20 fs time step. A 1.1 nm cut-off was used for reaction-field electrostatics and Van der Waals potentials^49^. Temperatures were held constant at 310 or 400 K (Supplementary Table 4) by the velocity rescaling^50^ thermostat (*τ*_*T*_ = 1 ps). Parrinello–Rahman^51^ semi-isotropic pressure coupling was applied to maintain a 1 bar reference pressure (*τ*_*P*_ = 12 ps, compressibility = 3 × 10^−4^). All images were rendered using PyMOL (v.2.5.0)^52^.

### Simulations system setup

Coarse-grained representations of AlphaFold2 protein models were generated using martinize2^53^, with secondary structure assignment by the DSSP (Dictionary of Secondary Structure in Proteins) algorithm^54^. Intermolecular and intramolecular orientations between backbone atoms within a 5–10 Å distance were constrained by an elastic network with a 500 kJ mol^−1^ nm^−2^ force constant, except for the flexible linkers (residue 164–172) in the full-length 16-mer. If specified in Supplementary Table 4, position restraints in *x*, *y* and *z* were applied to protein atoms with a 1,000 kJ mol^−1^ nm^−2^ force constant. Self-assembly runs (Supplementary Table 4a) were initiated from simulation boxes with a centred protein model and randomly placed DOPC molecules. DOPC membranes (Supplementary Table 4b,c) were built around the protein model using insane^55^. To avoid clashes between protein and lipid beads, DOPC molecules within 6 nm or 4 nm radii from the box centre (in *xy*) were removed from the system for the lower and upper bilayers, respectively (Supplementary Table 4b,c; see ‘initial setup’ in Extended Data Fig. 9c). All systems were solvated using Gromacs, including 150 mM NaCl and additional Na^+^ ions for charge neutralization. All simulations were preceded by energy minimization and a 5 ns number pressure temperature (NPT) equilibration.

Steps II–IV of simulating the CLCC1-induced fusion process (Supplementary Table. 4c and Extended Data Fig. 9c) required several additional settings:

- Step II: To break the periodicity of the bilayers, the simulation box was expanded by 90 Å in the *x* and *y* dimensions, followed by recentring and resolvation with water and ions as described. Flat-bottom potentials (*k* = 500 kJ mol^−1^ nm^−2^) were applied to retain all DOPC lipid molecules in a box-centred cylinder with a 240 Å radius and 200 Å height to prevent them from crossing the periodic boundaries.
- Step III: Van der Waals and electrostatic interactions between protein beads and the rest of the system (protein, lipid, water and ions) were switched off linearly using Gromacs’ lambda code (*λ* = 0 → *λ* = 1, with Δ*λ* = 10^−5 ^ns^−1^). The stochastic dynamics integrator was used with the flat-bottom potentials from step II. After completion, the now uncoupled protein atoms were deleted from the system.
- Step IV: Membrane periodicity was restored by removing all lipid and solvent molecules outside of a 300 × 300 × 230 Å^3^ system-centred box. Simulation was continued without flat-bottom potentials.

### CLCC1–lipid contact simulation analysis

For every frame (1 ns^−1^) in the three self-assembly simulations (Supplementary Table 4a), interactions (<7 Å) between the outermost protein sidechain beads (or backbone, for Gly) and any DOPC tail bead were counted. Contact frequency (%) was calculated as the fraction of simulation frames where a contact occurred, averaged over the eight dimers. The final contact map (Fig. 5f) was constructed by mapping the per-residue contact frequencies to the full-length CLCC1 AlphaFold model using the B-factor field in the PDB file (Supplementary Data 1, CLCC1_lipid_contact_map.pdb).

### Statistics and reproducibility

In vitro assays were run as biological triplicates—three independently seeded and processed cultures on separate days—to demonstrate reproducibility across preparations and reduce batch effects; this level of replication is standard for mechanistic cell-biology readouts. Figure 3k and Extended Data Fig. 6j contain sample sizes of *n* = 2 due to technical limitations, and therefore statistics are not derived. Fluorescence and transmission electron microscopy images are representative of at least *n* = 10 imaged cells, except for Extended Data Fig. 6k which is *n* = 5 imaged cells. Batch retest CRISPR screens in Fig. 1 were performed in biological duplicates because of the large sample sizes and results are shown as ‘combination’ scores derived from casTLE analysis. Proteomics in Fig. 3c were performed as technical duplicates which is standard protocol for such a large dataset.

For in vivo work, we used *n* > 4 mice per group, balancing statistical precision with the 3Rs (replacement, reduction, refinement). Based on prior or pilot variability for our endpoints, this sample size was expected to detect large effect sizes (≥(1.5–2.0) × s.d.); smaller effects were outside the scope of this study and would motivate a larger, confirmatory cohort. Mice were divided between males and females and sexes are specified in figure legends and methods. All mouse experiments were produced twice with similar results, except for Fig. 2g,h and Extended Data Fig. 2j, k, which could only be performed as one replicate but were further supported by Fig. 2i,j and Extended Data Fig. 2l.

All FIB-SEM data in Fig. 5, Extended Data Fig. 7, and Supplementary Fig. 15 have a sample size of *n* = 1 cell because of the high density of data per cell.

All statistical analyses were performed using Prism 9 (GraphPad). For each panel, the number of biological replicates (*n*), *P* values, and statistical tests employed are reported in figure legends and methods.

### Reporting summary

Further information on research design is available in the Nature Portfolio Reporting Summary linked to this article.

## Online content

Any methods, additional references, Nature Portfolio reporting summaries, source data, extended data, supplementary information, acknowledgements, peer review information; details of author contributions and competing interests; and statements of data and code availability are available at 10.1038/s41586-025-10064-4.

## Supplementary information

Supplementary Information This file contains Supplementary Discussion, Supplementary Figs. 1–15 and Supplementary References.

Reporting Summary

Supplementary Table 1 CRISPR–Cas9 genetic screen data. Full casTLE results for the CRISPR–Cas9 screen employing the genome-wide library of sgRNAs and batch retest sublibrary of sgRNAs under different metabolic conditions.

Supplementary Table 2 LD and metabolism batch retest library. List of target genes, sgRNA sequences, and primer sequences used for amplification.

Supplementary Table 3 Proteomics of LD-enriched buoyant fractions. List of proteins identified in proteomics analyses of LD-enriched buoyant fractions isolated from control and *CLCC1*-KO cells.

Supplementary Table 4 Summary of coarse-grained molecular dynamics simulations. A) Self-assembly coarse-grained molecular dynamics started from randomly placed lipids in a simulation box with the protein at the centre. B) Coarse-grained molecular dynamics simulations of proteins embedded in a single DOPC bilayer. C) Series of coarse-grained molecular dynamics simulations of the CLCC1 16-mer between two DOPC bilayers. I–IV correspond to the labels in Extended Data Fig. 9c. Each run was initiated from the final frame of the previous simulation.

Supplementary Table 5 RNA sequencing analyses of transcript alterations in *CLCC1*-KO cells. 

Supplementary Data 1 CLCC1 lipid contact map. The final contact map (PDB file) mapping the per-residue contact frequencies to the full-length CLCC1 AlphaFold model using the B-factor field.

Supplementary Data 2 Unprocessed blots for Figs. 2 and 3, Extended Data Figs. 2–5 and 9 and Supplementary Figs. 3–5, 8 and 11.

Supplementary Data 3 Source data files for Supplementary Figs. 3, 4 and 13.

Supplementary Video 1 FIB-SEM of *Clcc1*-HepKO mouse liver tissue. FIB-SEM analysis of liver tissue from *Clcc1*-HepKO mouse.

Supplementary Video 2 FIB-SEM of control mouse liver tissue. FIB-SEM analysis of liver tissue from control mouse.

Supplementary Video 3 Side view of CLCC1 Brl1p homology domain multimer (ribbon). Side view of 8 subunits of the CLCC1 Brl1p homology domain (ribbon) using ColabFold.

Supplementary Video 4 Top view of CLCC1 Brl1p homology domain multimer (ribbon). Top view of 16 subunits of the CLCC1 Brl1p homology domain (ribbon) using ColabFold.

Supplementary Video 5 Side view of CLCC1 Brl1p homology domain multimer (space-filling). Side view of 8 subunits of the CLCC1 Brl1p homology domain (space-filling) using ColabFold.

Supplementary Video 6 Side view of Brr6 multimer (ribbon). Side view of 8 subunits of Brr6 (ribbon) using ColabFold.

Supplementary Video 7 Top view of Brr6 multimer (ribbon). Top view of 16 subunits of Brr6 (ribbon) using ColabFold.

Supplementary Video 8 Side view of Brr6 multimer (space-filling). Side view of 8 subunits of Brr6 (space-filling) using ColabFold.

## Source data

Source Data Fig. 2

Source Data Fig. 3

Source Data Fig. 4

Source Data Fig. 5

Source Data Extended Data Fig. 1

Source Data Extended Data Fig. 2

Source Data Extended Data Fig. 4

Source Data Extended Data Fig. 5

Source Data Extended Data Fig. 6

Source Data Extended Data Fig. 7

## Data Availability

The custom human VLDM sgRNA library will be shared upon request from the corresponding authors. Raw data for Fig. 1b,c and Extended Data Fig. 1e,h–r can be accessed in Supplementary Table 1. Raw data for Fig. 3c,j can be accessed in Supplementary Data 3. Raw data for Fig. 5f and Extended Data Fig. 9 can be accessed in Supplementary Table 4. Raw data for Extended Data Fig. 1f and Supplementary Fig. 1 are publicly available from the STRING database (https://string-db.org). Raw data for Supplementary Fig. 2 are publicly available on the Multi-marker Analysis of GenoMic Annotation (MAGMA) database (https://cncr.nl/ctg). Raw data for Supplementary Fig. 9 are publicly available from FIREWORKS (https://mendillolab.shinyapps.io/fireworks) and the Native Organelle Immunoprecipitation database (https://organelles.sf.czbiohub.org). All unique and stable reagents generated in this study are available from the corresponding author with a completed Materials Transfer Agreement. Source data are provided with this paper.

## Code availability

The custom codes for quantification of nuclear pores and PLIN2 signal around LDs are available at https://github.com/gparlakgul/nuclear_pore (tag v.1.0, commit 7a1f93d4e345a004c00396701c634e1782dcddfb). The repository contains a readme and environment specification. The custom code to perform molecular dynamics simulations is available at 10.5281/zenodo.17459728 (ref. ^56^).
