# Supplementary material for: CLCC1 promotes hepatic neutral lipid flux and nuclear pore complex assembly
Source: Nature. 2026 Feb 25;652(8109):462–70. doi: 10.1038/s41586-025-10064-4 (PMC13061601; doi:10.1038/s41586-025-10064-4)
Supplement: Supplementary file 1 — This file contains Supplementary Discussion, Supplementary Figs. 1–15 and Supplementary References. [file 41586_2025_10064_MOESM1_ESM.pdf]

nature portfolio

https://doi.org/10.1038/s41586-025-10064-4

Supplementary information

CLCC1 promotes hepatic neutral lipid flux and nuclear pore complex assembly

In the format provided by the authors and unedited

Supplementary Discussion

MFH domain and structural models

One conserved feature of MFH domains is the separation of TMH1 and TMH2 by the juxtamembrane AH (AH1 in CLCC1), which leads to the TMHs being unconstrained by each other, so that they need not be parallel. This allows MFH proteins to reside in curved membranes. A second feature is that all of TMH1, AH1 and TMH2 are strongly predicted by Alphafold Multimer [and similar algorithms - same results from DMFold and Multifold] to contain extensive interfaces that oligomerize side-by-side. The whole domain has a triangular cross-section, as shown by predicted dimers with the AHs pointing in different directions and the overall shape, which is the basis upon which ring oligomers can be predicted[1]. These features together provide the first explanation at a molecular/physical level for how Brl1p / Brr6p impact NPC biogenesis, which has been missing since they were first linked genetically to this process. Finally, MD mapping of lipid interactions identified a second, shorter AH (i.e., AH2) within the lumenal knuckle region of the human protein, which forms a lumenal hydrophobic face of the predicted CLCC1 oligomer. We obtained ring structures between 12-20 protomers using Alphafold Multimer, with the major difference being the size of the central pore (1.4-5.8nm). Determining the stoichiometry of this putative oligomer is an important research direction that could shed light on the CLCC1 mechanism of action. Our MD simulations suggest a potential mechanism in which CLCC1 dimers dynamically associate to form a homo-oligomer. Our MD simulations of predicted CLCC1 dimer and 16-mer structures show their ability to locally bend the membrane. Assembly of the ring-shaped homo-oligomer, causes the TMHs to adopt increasingly greater angles away from those that cross the membrane perpendicularly, so bending the membrane inwards (Supplementary Fig. 12a,b), and also

positions the lumenal AHs to insert into and drive association with an adjacent membrane, ultimately forming a fusion intermediate that is later resolved following oligomer disassembly. Brl1p and Brl6p lack the second AH, and it may be that the yeast and human proteins employ divergent mechanisms to promote membrane fusion. Indeed, modeling suggests the possibility of trans interactions between Brl1p and/or Brr6p on opposing inner and outer NE membranes (Supplementary Fig. 12c), raising the possibility that interactions between opposing oligomers could also promote fusion. A limitation of our study is the analysis of predicted AlphaFold CLCC1 structures, and experimentally determining the structure of different oligomeric forms of CLCC1 is an important future direction.

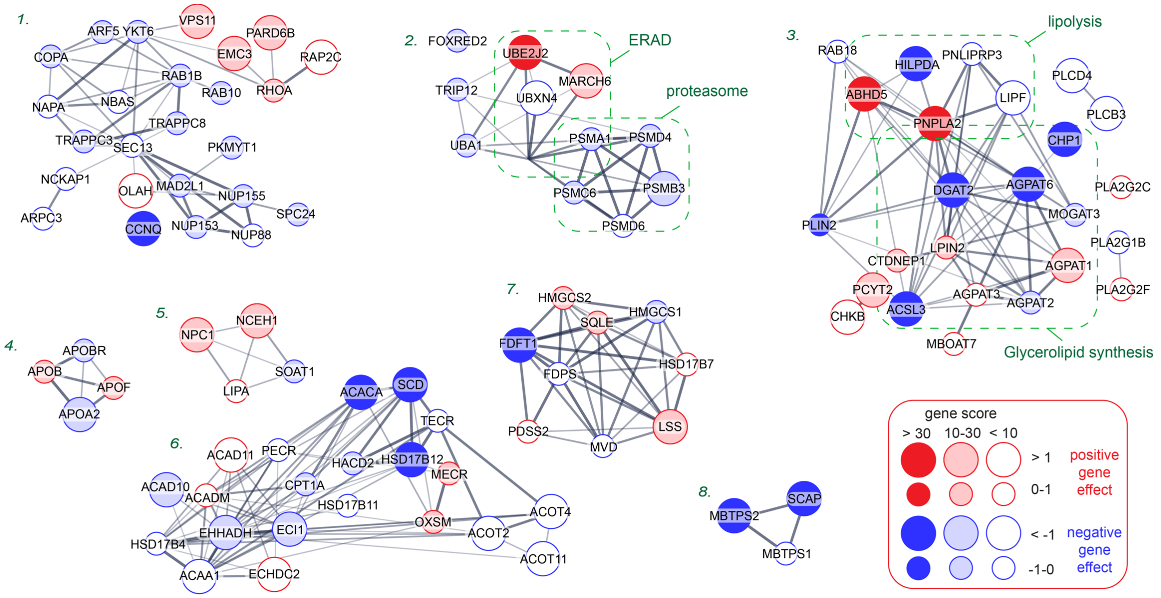

Supplementary Figure 1. Network analysis of enriched gene clusters.

Significant gene clusters from Extended Data Fig 1F. Nodes are marked based on directionality of effect (red or blue), gene effect size, and confidence score.

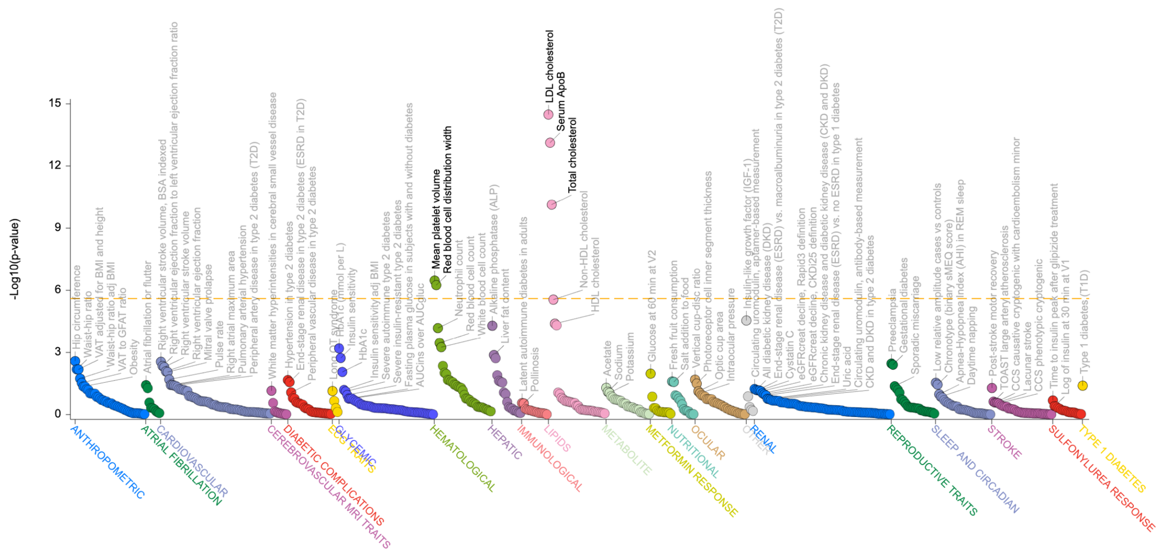

Phenotype	P-Value	Variants	Z-Stat	Sample Size
LDL cholesterol	3.27e-15	-	7.79	2,099,821
Serum ApoB	7.60e-14	34	7.39	434,341
Total cholesterol	7.39e-11	-	6.41	1,912,019
Mean platelet volume	3.39e-7	-	4.97	830,950
Red blood cell distribution width	5.64e-7	-	4.87	941,639
Non-HDL cholesterol	2.82e-6	-	4.54	994,596

Supplementary Figure 2. Genetic association of CLCC1 variants with altered serum lipids.

Common variant gene-level associations for CLCC1 from the Common Metabolic Diseases Knowledge Portal. The plot shows phenotypic associations for CLCC1 based upon genetic associations using Multi-marker Analysis of GenoMic Annotation (MAGMA)[2].

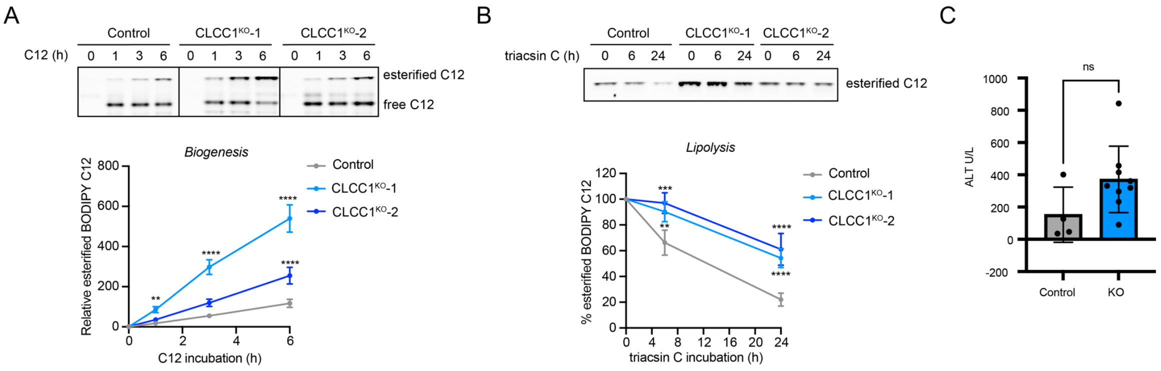

Supplementary Figure 3. Analysis of lipid metabolism and markers.

A) Representative TLC resolving esterified and free BODIPY C12 558/568 in Huh7 cells expressing a safe targeting sgRNA or sgRNAs against CLCC1. Cells were incubated with BODIPY C12 for the indicated times, followed by lipid extraction and TLC. A graph of the quantification of esterified BODIPY C12 levels is shown. BODIPY C12 levels at each time point were quantified relative to time 0 for each cell line. Data represent mean ± SD of three biological replicates. ** *p<0.01 and ****p<0.0001 by two-way ANOVA with Dunnett's multiple comparisons test.*

B) Representative TLC resolving esterified BODIPY C12 558/568 in Huh7 cells expressing a safe targeting sgRNA or sgRNAs against CLCC1. Cells were incubated with BODIPY C12 for 16 h followed by triacsin C treatment for the indicated times, followed by lipid extraction and TLC. A graph of the quantification of esterified BODIPY C12 levels is shown. BODIPY C12 levels at each time point were quantified relative to time 0 for each cell line. Data represent mean ± SD of three biological replicates. ***p<0.01, **p<0.001 and ****p<0.0001 by two-way ANOVA with Dunnett's multiple comparisons test.*

C) Quantification of ALT from clinical analyzer. Data represent mean ± SD of > four mice. *ns, not significant (P ≥ 0.05) by two-way ANOVA with Dunnett's multiple comparisons test.*

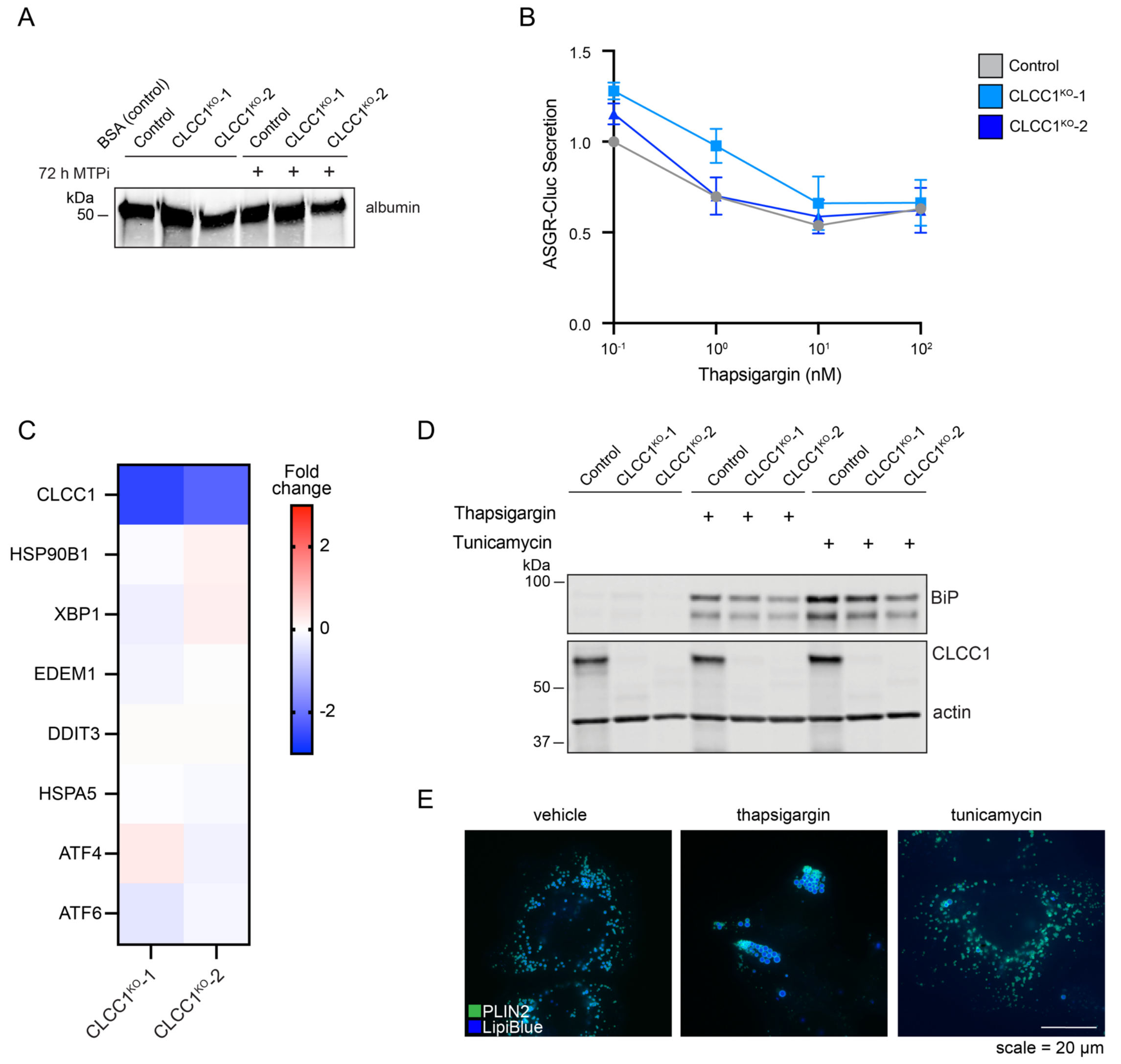

Supplementary Figure 4. ER stress and secretion are unchanged in CLCC1^{KO} cells.

A) Immunoblot of albumin secretion from control and CLCC1^{KO} cells. Conditioned serum-free media was collected and precipitated before immunoblotting.

B) Quantification of ASGR-Cluc secretion in CLCC1^{KO} cells across three biological replicates.

C) Fold change of selected mRNA transcripts in CLCC1^{KO} cells relative to control cells, measured using RNA sequencing. See Supplementary Table 5.

D) Immunoblot of BiP levels in control and CLCC1^{KO} cells treated with 5 µg/mL tunicamycin or 1 µM thapsigargin for 24 h.

E) Representative fluorescence microscopy images of PLIN2 (green) and LDs (blue) in Huh7 cells treated with ER stress inducers thapsigargin and tunicamycin. Scale bar represents 20 µm.

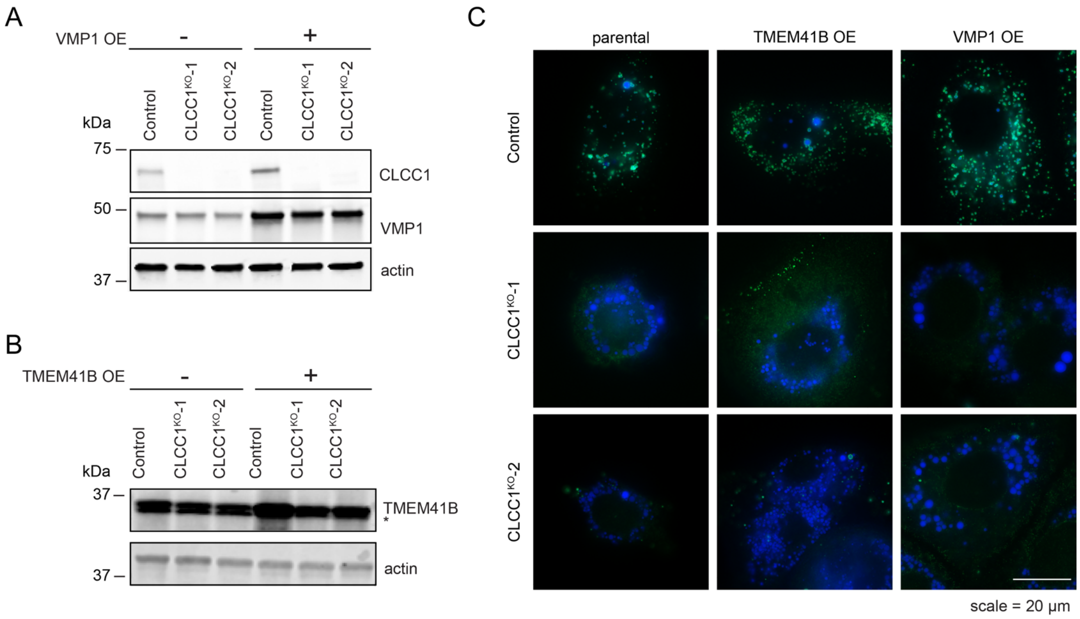

Supplementary Figure 5. Analysis of ER scramblases in CLCC1^{KO} cells.

A) Immunoblot of VMP1 overexpression in control and CLCC1^{KO} cells.

B) Immunoblot of TMEM41B overexpression in control and CLCC1^{KO} cells.

C) Representative fluorescence microscopy images of PLIN2 (green) and LDs (blue) in control and CLCC1^{KO} Huh7 cells overexpressing TMEM41B and VMP1, as indicated. Scale bar represents 20 µm.

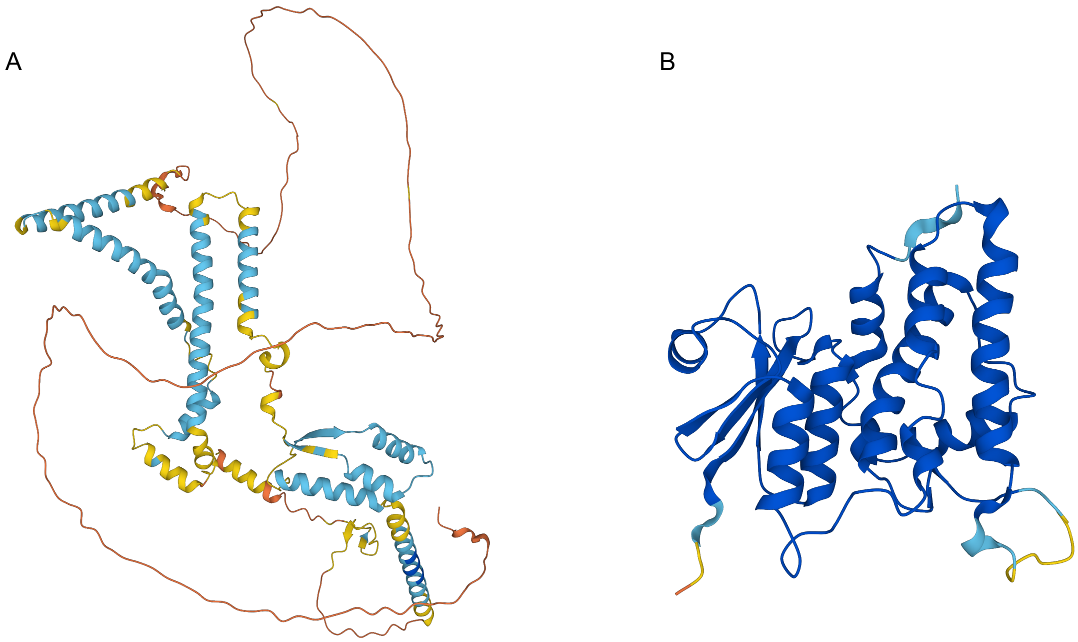

Supplementary Figure 6. CLCC1 and CLIC1 Alphafold structural predictions.

A, B) Alphafold structural predictions of CLCC1 and CLIC1. Images colored by pLDDT (predicted local distance difference test), where blue indicates a confident prediction (light – high, dark – very high), while yellow/orange/red represent predictions of progressively low confidence.

A Human ➡ yeast. Seed CLCC1 (551 aa)

>NP_011901.1 **Brl1p** [Saccharomyces cerevisiae S288c]

Probability: 93.92%, E-value: 0.29, Score: 47.42, Aligned cols: 150, Identities: 11%, Similarity: 0.104, Template Neff: 4.6

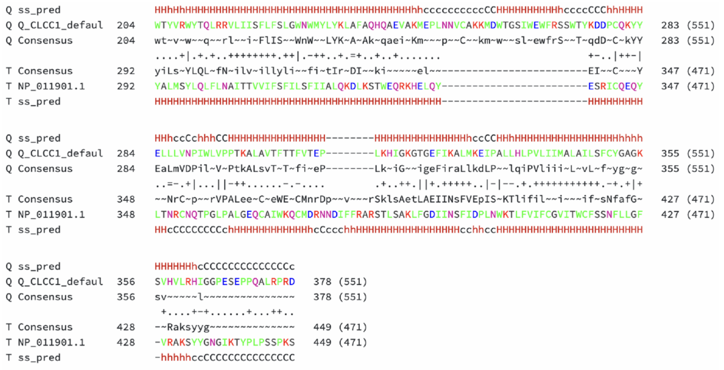

>NP_011267.1 **Brr6p** [Saccharomyces cerevisiae S288c]

Probability: 86.26%, E-value: 2.5, Score: 35.67, Aligned cols: 124, Identities: 15%, Similarity: 0.157, Template Neff: 5.8

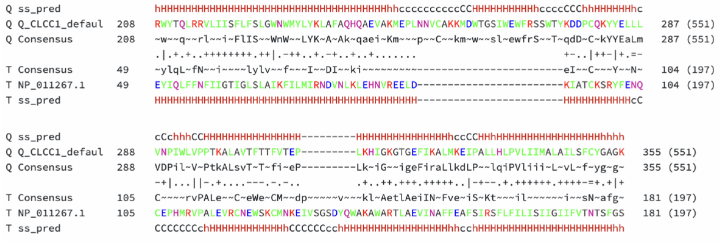

B Yeast ➡ human.

Seed: Brl1 (471 aa)

>NP_055942.1 **chloride channel CLIC-like protein 1 isoform 2 precursor** [Homo sapiens]

Probability: 95.72%, E-value: 3, Score: 42.02, Aligned cols: 143, Identities: 13%, Similarity: 0.101, Template Neff: 4.1

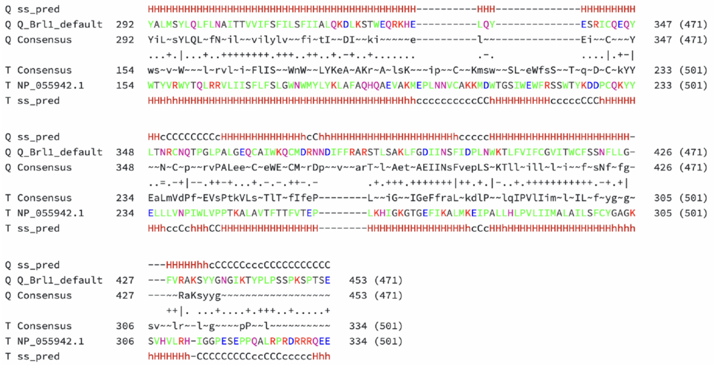

Seed: Brr6 (197 aa)

>NP_055942.1 **chloride channel CLIC-like protein 1 isoform 2 precursor** [Homo sapiens]

Probability: 94.87%, E-value: 2, Score: 50, Aligned cols: 153, Identities: 10%, Similarity: 0.102, Template Neff: 4.1

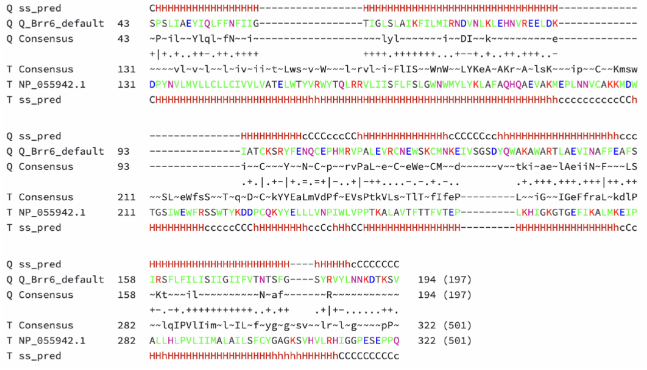

Supplementary Figure 7. Structure homology analysis of CLCC1 with Brl1p and Brr6p.

HHpred remote homology searches using default settings: (A) in S. cerevisiae using human CLCC1 as the query protein; (B) in human using S. cerevisiae Brl1p or Brr6p as query proteins.

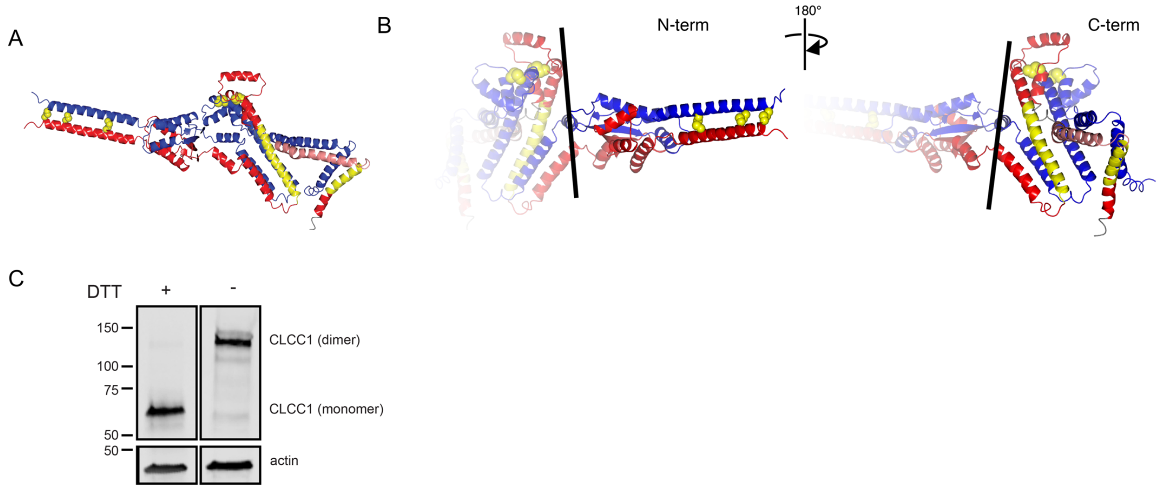

Supplementary Figure 8. CLCC1 forms a disulfide-stabilized dimer.

A) Predicted disulfide-stabilized CLCC1 dimer. Q96S66_V1_5 created by the Levy lab[1] (downloaded from 3D-Beacons database) Colors: one protomer as in Fig 4B, one all blue.

B) Additional visualizations highlighting the N- and C-termini of the predicted disulfide-stabilized CLCC1 dimer structure. The two protomers are colored as in Fig 4B.

C) Western blot analysis of CLCC1 in the presence and absence of the reducing agent DTT.

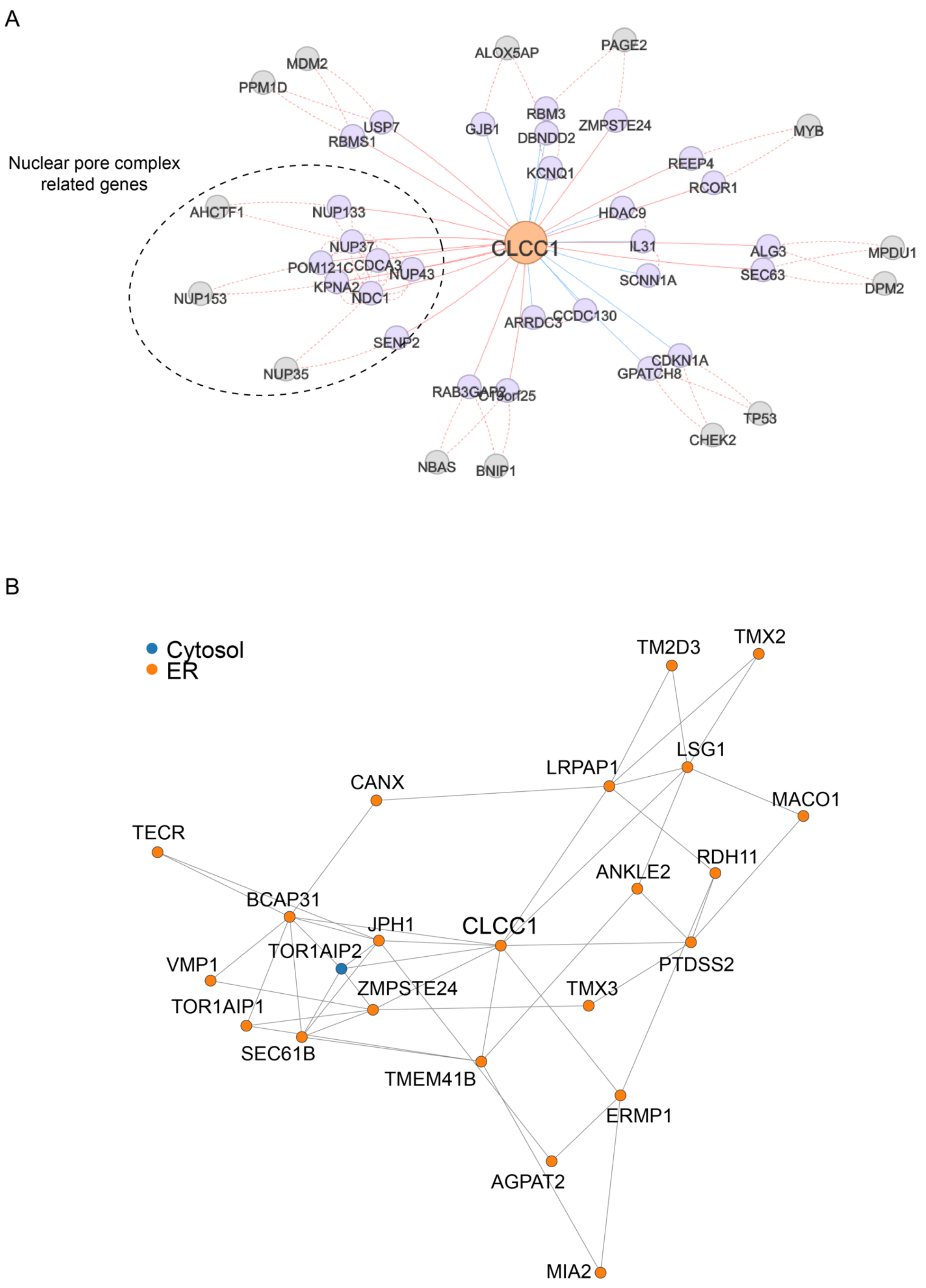

Supplementary Figure 9. Analyses of CLCC1 co-essentiality and localization neighborhood.

A) CLCC1 co-essentiality network using FIREWORKS interactive web tool to reveal gene-gene relationships[3].

B) CLCC1 localization neighborhood using proteomic profiling data of affinity purified organelles[4].

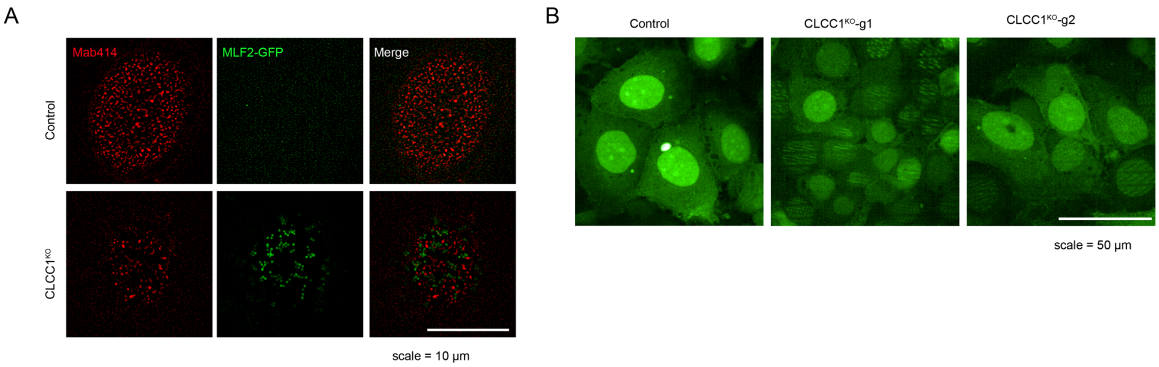

Supplementary Figure 10. Analysis of NPCs and nucleocytoplasmic transport in CLCC1^{KO} cells.

A) Superresolution images of FG-rich nucleoporins (red) and nuclear blebs (green) in control and CLCC1^{KO} Huh7 cells. Scale bar represents 10 µm. (See quantification in Extended Data Fig 6K)

B) Representative confocal images of GFP-NLS-NES expression in control and CLCC1^{KO} Huh7 cells. (See quantification in Extended Data Fig 6L)

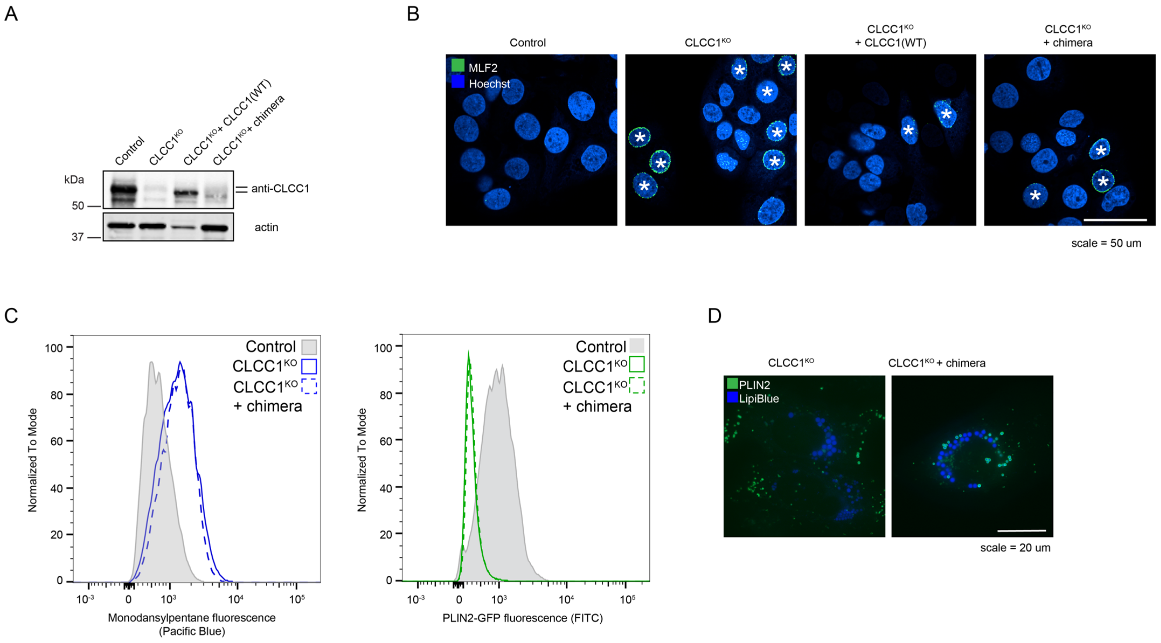

Supplementary Figure 11. Analysis of the CLCC-Brl1p chimera.

A) Immunoblot of CLCC1 or CLCC1/Brl1 chimera in control and CLCC1^{KO} Huh7 cells.

B) Representative confocal images of MLF2-GFP in control, CLCC1^{KO}, CLCC1^{KO} + CLCC1 (WT), and CLCC1^{KO} + CLCC1/Brl1 chimera Huh7 cells. Cells with MLF2-GFP foci are marked with a white asterisk. Scale bar represents 50 µm.

C) Flow cytometry histograms of monodansylpentane and PLIN2-GFP fluorescence in control, CLCC1^{KO}, and CLCC1^{KO} + CLCC1/Brl1 chimera Huh7 cells.

D) Representative fluorescence microscopy images of PLIN2-GFP and LDs in CLCC1^{KO} and CLCC1^{KO} + CLCC1/Brl1 chimera Huh7 cells. LDs were stained with 500 nM Lipi-Blue. Scale bar represents 20 µm.

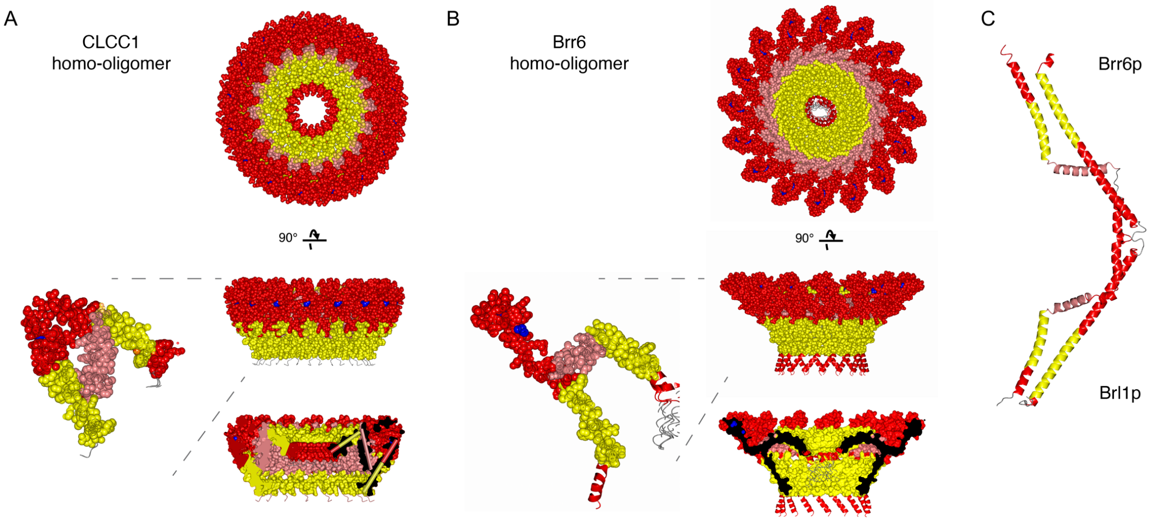

Supplementary Figure 12. Alphafold and Colabfold structural predictions.

A) Colabfold structural prediction of CLCC1 (205-360aa) homo-oligomer (16-subunits). Colors: yellow =TMH, pink = AH, red = other helix, blue spheres = conserved cysteines.

B) Colabfold structural prediction of Brr6p (28-197aa) homo-oligomer (16-subunits). Colors as in (A).

C) RoseTTAFold structural prediction of Brr6p/Brl1p heterodimer (38-197aa & 281-438)[5]. Colors as in (A).

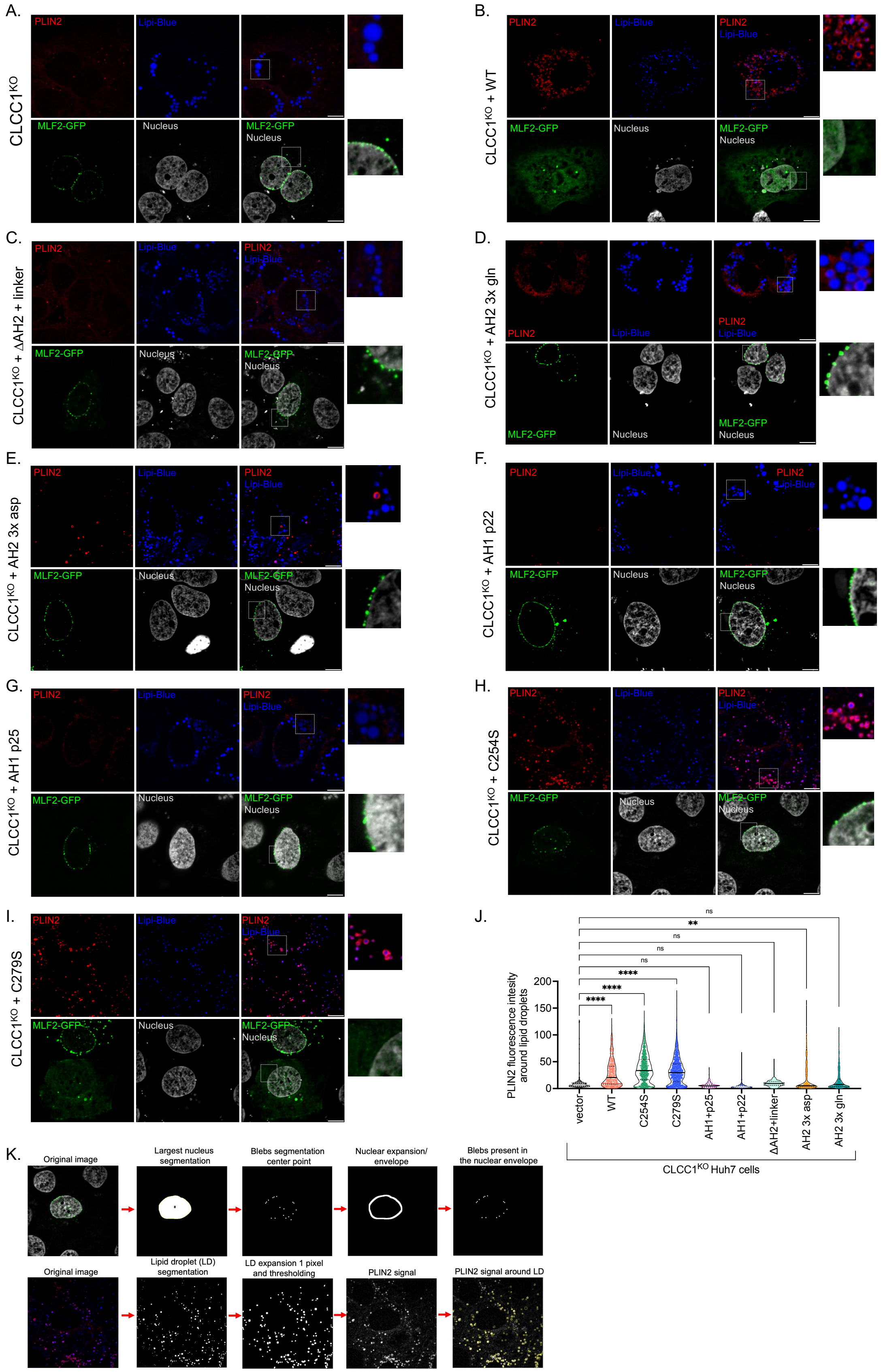

A.
CLCC1KO
PLIN2
Lipi-Blue
MLF2-GFP
Nucleus
B.
CLCC1KO + WT
C.
CLCC1KO + ΔAH2 + linker
D.
CLCC1KO + AH2 3x gln
E.
CLCC1KO + AH2 3x asp
F.
CLCC1KO + AH1 p22
G.
CLCC1KO + AH1 p25
H.
CLCC1KO + C254S
I.
CLCC1KO + C279S
J.
PLIN2 fluorescence intesity around lipid droplets
200
150
100
50
0
ns
**
****
vector
WT
C254S
C279S
AH1+p25
AH1+p22
ΔAH2+linker
AH2 3x asp
AH2 3x gln
CLCC1KO Huh7 cells
K.
Original image
Largest nucleus segmentation
Blebs segmentation center point
Nuclear expansion/ envelope
Blebs present in the nuclear envelope
Lipid droplet (LD) segmentation
LD expansion 1 pixel and thresholding
PLIN2 signal
PLIN2 signal around LD

Supplementary Figure 13. Immunofluorescence analysis and quantification of the effects of CLCC1 mutants on nuclear bleb formation and lipid droplet emergence.

Confocal imaging of $CLCC1^{KO}$ Huh7 cells stably expressing (A) empty vector, (B) full length (C) CLCC1, ∆AH2 + linker, (D) AH2 3x glutamine, (E) AH2 3x aspartate, (F) AH1 p22, (G) AH1 p25, (H) C254S, (I) C279S. Cells were transiently transfected with MLF2-GFP construct to mark nuclear membrane herniations, immunostained with anti-PLIN2 antibodies to assess cytoplasmic LD biogenesis, stained with LipiBlue to mark LDs, and stained with NuclearMask to mark nuclei. (J) Quantification of PLIN2 signal intensity surrounding lipid droplets for the indicated constructs. Quantification was performed using the analysis pipeline outlined in (K). Data represent mean +/- s.e.m ***p<0.001 and ****p<0.0001 by one-way ANOVA with Dunnett's multiple comparisons test. ns, not-significant (P ≥ 0.05).* Images are representative of two independent experiments. Scale bar = 10 μm.

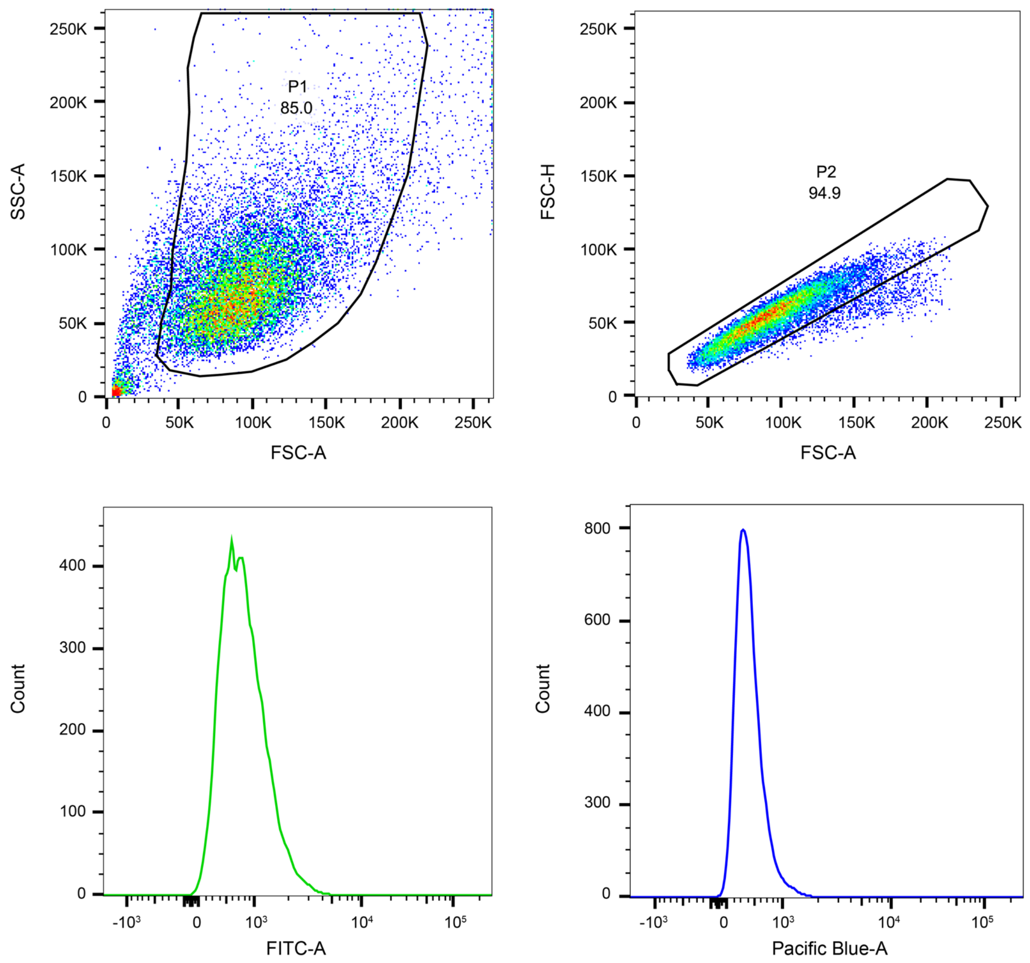

Supplementary Figure 14. Representative flow cytometry analysis gates.

Representative gating strategies are shown for analysis of Huh7 cells FITC (e.g., BODIPY 493/503 and PLIN2-GFP) and Pacific Blue (e.g., Monodansylpentane) fluorescence.

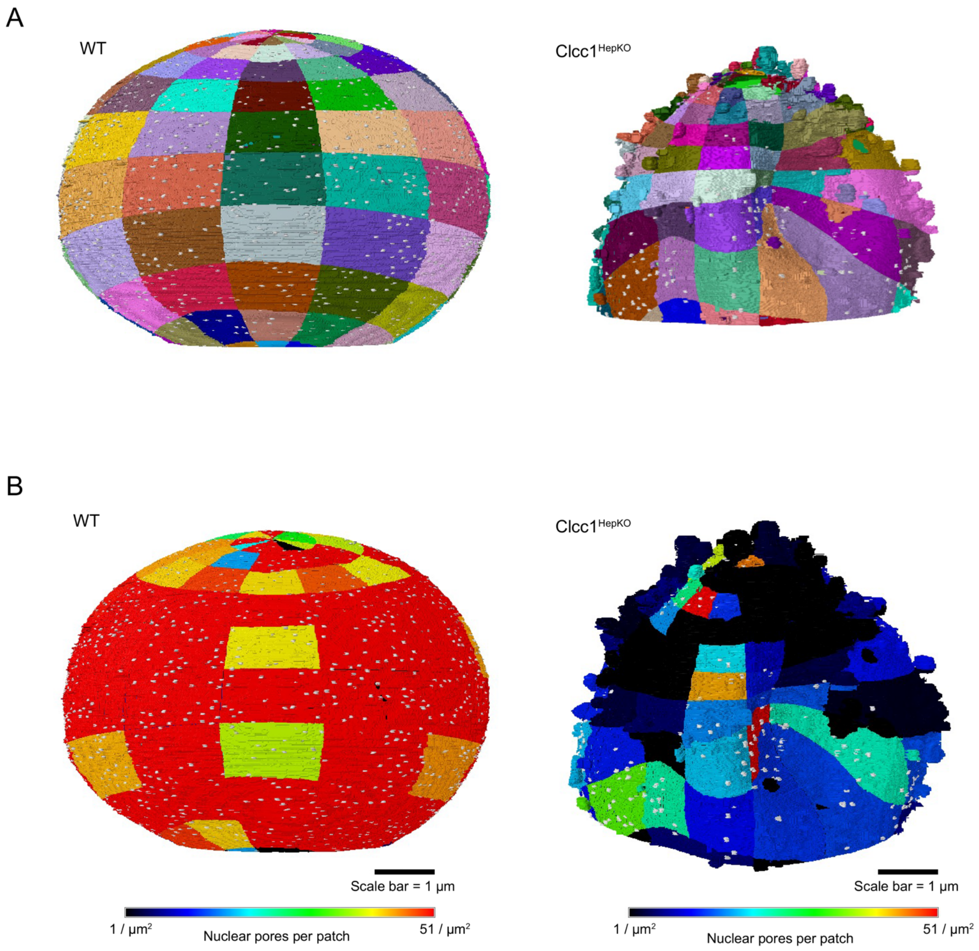

Supplementary Figure 15. FIB-SEM analysis of nuclear pore and bleb density.

A) Quantification of nuclear pore and bleb densities was achieved by segmenting the nuclear membrane surface into approximately 100 distinct patches using a custom Python script (described in Methods). Each patch was assigned a unique color for identification and visualization purposes.

B) Using the patches defined in panel A, the number of nuclear pores and blebs per patch was quantified and visualized according to the corresponding color scale.

Supplemental Table Legends

Supplementary Table 1. CRISPR-Cas9 genetic screen data.

Full casTLE results for the CRISPR-Cas9 screen employing the genome-wide library of sgRNAs and batch retest sublibrary of sgRNAs under different metabolic conditions.

Supplementary Table S2. Lipid droplet and metabolism batch retest library.

List of target genes, sgRNA sequences, and primer sequences used for amplification.

Supplementary Table S3. Proteomics of LD-enriched buoyant fractions.

List of proteins identified in proteomics analyses of LD-enriched buoyant fractions isolated from control and CLCC1^{KO} cells.

Supplementary Table 4. Summary of CG MD simulations.

A) Self-assembly CG MD started from randomly placed lipids in a simulation box with the protein at the centre.

B) CG MD simulations of proteins embedded in a single DOPC bilayer.

C) Series of CG MD simulations of the CLCC1 16-mer between two DOPC bilayers. I-IV correspond to the labels in Extended Data Fig 22D. Each run was initiated from the final frame of the previous simulation.

Supplemental Data Legend

Supplementary Data 1. CLCC1 lipid contact map.

The final contact map (PDB file) mapping the per-residue contact frequencies to the full-length CLCC1 AlphaFold model using the B-factor field.

Supplemental Video Legends

Supplementary Video 1. FIB-SEM of Clcc1HepKO mouse liver tissue

Focused ion beam scanning EM (FIB-SEM) analysis of liver tissue from Clcc1HepKO mouse.

Supplementary Video 2. FIB-SEM of control mouse liver tissue

Focused ion beam scanning EM (FIB-SEM) analysis of liver tissue from control mouse.

Supplementary Video 3. Side view of CLCC1 Brl1p homology domain multimer (ribbon)

Side view of 8 subunits of the CLCC1 Brl1p homology domain (ribbon) using ColabFold.

Supplementary Video 4. Top view of CLCC1 Brl1p homology domain multimer (ribbon)

Top view of 16 subunits of the CLCC1 Brl1p homology domain (ribbon) using ColabFold.

Supplementary Video 5. Side view of CLCC1 Brl1p homology domain multimer (space-filling)

Side view of 8 subunits of the CLCC1 Brl1p homology domain (space-filling) using ColabFold.

Supplementary Video 6. Side view of Brr6 multimer (ribbon)

Side view of 8 subunits of Brr6 (ribbon) using ColabFold.

Supplementary Video 7. Top view of Brr6 multimer (ribbon)

Top view of 16 subunits of Brr6 (ribbon) using ColabFold.

Supplementary Video 8. Side view of Brr6 multimer (space-filling)

Side view of 8 subunits of Brr6 (space-filling) using ColabFold.

Supplementary References

1. Schweke, H. *et al.* An atlas of protein homo-oligomerization across domains of life. *Cell* **187**, 999-1010.e15 (2024).
2. De Leeuw, C. A., Mooij, J. M., Heskes, T. & Posthuma, D. MAGMA: Generalized Gene-Set Analysis of GWAS Data. *PLoS Comput Biol* **11**, e1004219 (2015).
3. Amici, D. R. *et al.* FIREWORKS: a bottom-up approach to integrative coessentiality network analysis. *Life Sci Alliance* **4**, (2021).
4. Hein, M. Y. *et al.* Global organelle profiling reveals subcellular localization and remodeling at proteome scale. Preprint at https://doi.org/10.1101/2023.12.18.572249 (2023).
5. Humphreys, I. R. *et al.* Computed structures of core eukaryotic protein complexes. *Science* **374**, eabm4805 (2021).
