# Supplementary material for: CLCC1 promotes hepatic neutral lipid flux and nuclear pore complex assembly
Source: Nature. 2026 Feb 25;652(8109):462–70. doi: 10.1038/s41586-025-10064-4 (PMC13061601; doi:10.1038/s41586-025-10064-4)
Supplement: Supplementary file 2 — Reporting Summary [file 41586_2025_10064_MOESM2_ESM.pdf]

nature portfolio

Corresponding author(s): James Olzmann

Last updated by author(s): Jan 14, 2025

Reporting Summary

Nature Portfolio wishes to improve the reproducibility of the work that we publish. This form provides structure for consistency and transparency in reporting. For further information on Nature Portfolio policies, see our Editorial Policies and the Editorial Policy Checklist.

Statistics

For all statistical analyses, confirm that the following items are present in the figure legend, table legend, main text, or Methods section.

n/a	Confirmed	
☐	☒	The exact sample size (*n*) for each experimental group/condition, given as a discrete number and unit of measurement
☐	☒	A statement on whether measurements were taken from distinct samples or whether the same sample was measured repeatedly
☐	☒	The statistical test(s) used AND whether they are one- or two-sided<br>*Only common tests should be described solely by name; describe more complex techniques in the Methods section.*
☒	☐	A description of all covariates tested
☐	☒	A description of any assumptions or corrections, such as tests of normality and adjustment for multiple comparisons
☐	☒	A full description of the statistical parameters including central tendency (e.g. means) or other basic estimates (e.g. regression coefficient) AND variation (e.g. standard deviation) or associated estimates of uncertainty (e.g. confidence intervals)
☐	☒	For null hypothesis testing, the test statistic (e.g. *F*, *t*, *r*) with confidence intervals, effect sizes, degrees of freedom and *P* value noted<br>*Give P values as exact values whenever suitable.*
☒	☐	For Bayesian analysis, information on the choice of priors and Markov chain Monte Carlo settings
☒	☐	For hierarchical and complex designs, identification of the appropriate level for tests and full reporting of outcomes
☐	☒	Estimates of effect sizes (e.g. Cohen's *d*, Pearson's *r*), indicating how they were calculated

Our web collection on statistics for biologists contains articles on many of the points above.

Software and code

Policy information about availability of computer code

Data collection	BD FACSDiva v6.2 (BD BIOSCIENCES), Image Lab v6.0.1 (Bio-Rad Laboratories), ZEISS ZEN v3.2(ZEISS Microscopy), ZEN 3.0 SR Black, v16 (ZEISS Microscopy), Odyssey v3.0 (LI-COR Biosciences), ChemiDoc MP Imaging System (Bio-Rad Laboratories), PathologyMap 2.0 (HistoWiz), nAKTA fast-protein liquid chromatography (FPLC) (Amersham Pharmacia Biotech), GMS3 software (Gatan Inc.), Dragonfly 2022.2 (Comet Technologies), i-control (Tecan),
Data analysis	BowTie 2 v2.3.4.3, casTLE statistical framework vl.0, Prism v9 (Graphpad Software), DESeq2 vl.5, Partek Flow (Illumina) FlowJo vl0 (BD Biosciences), Fiji/ImageJ vl.53e (NIH), Harmony High Content Image Analysis Software v4.9 (Perkin Elmer), STRING vl2.0, Morpheus v1.0 (Broad Institute), Image Lab v6.0.l (Bio-Rad Laboratories), Napari v0.4.18, Proteome Discoverer 2.4 (Thermo Fisher Scientific), Colaborary (Google), COSMIC2, DMFold, MultiFOLD, trRosetta, AlphaFold2, Gromacs v2023.3, Martini 3 force field v3.0.0, PyMOL v2.5.0, martinize2 (python 3 package) Arivis Pro 4.2 (ZEISS Microscopy) Github repository for custom python 3.0 script for dividing nucleus into patches and ImageJ macro for measuring PLIN2 signal around LDs: https://github.com/gparlakgul/nuclear_pore

For manuscripts utilizing custom algorithms or software that are central to the research but not yet described in published literature, software must be made available to editors and reviewers. We strongly encourage code deposition in a community repository (e.g. GitHub). See the Nature Portfolio guidelines for submitting code & software for further information.

Data

Policy information about availability of data

All manuscripts must include a data availability statement. This statement should provide the following information, where applicable:

- Accession codes, unique identifiers, or web links for publicly available datasets
- A description of any restrictions on data availability
- For clinical datasets or third party data, please ensure that the statement adheres to our policy

All data that support the conclusions in this manuscript are available from the corresponding author upon reasonable request. Raw data for Figure 1C, Figure 1F, Extended Data Figure 1C-D, and Extended Data Figure 2 can be accessed in Supplementary Dataset 1. Raw data for Extended Data Figure 4D can be accessed in Supplementary Dataset 3. Raw data for Raw data for Extended Data Figure 22 can be accessed in Supplementary Dataset 4. Raw data for Extended Data Figure 9C can be accessed in Supplementary Dataset 5. Raw data for Figure 15A are publicly available from from FIREWORKS (mendillolab.shinyapps.io/fireworks/). Raw data for Extended Data Figure 15B are publicly available from the Native Organelle Immunoprecipitation database (organelles.sf.czbiohub.org).

Research involving human participants, their data, or biological material

Policy information about studies with human participants or human data. See also policy information about sex, gender (identity/presentation), and sexual orientation and race, ethnicity and racism.

Reporting on sex and gender	N/A
Reporting on race, ethnicity, or other socially relevant groupings	N/A
Population characteristics	N/A
Recruitment	N/A
Ethics oversight	N/A

Note that full information on the approval of the study protocol must also be provided in the manuscript.

Field-specific reporting

Please select the one below that is the best fit for your research. If you are not sure, read the appropriate sections before making your selection.

☒ Life sciences ☐ Behavioural & social sciences ☐ Ecological, evolutionary & environmental sciences

For a reference copy of the document with all sections, see nature.com/documents/nr-reporting-summary-flat.pdf

Life sciences study design

All studies must disclose on these points even when the disclosure is negative.

Sample size	In vitro assays were run as biological triplicates—three independently seeded and processed cultures on separate days—to demonstrate reproducibility across preparations and reduce batch effects; this level of replication is standard for mechanistic cell-biology readouts. Figure 3K and Extended Data Figure 6J contain sample sizes of n=2 due to technical limitations, and therefore statistics are not derived. Fluorescence and transmission electron microscopy images are representative of at least n= l0 imaged cells, except for Extended Data Figure 18A which is n=5 imaged cells. Batch retest CRISPR screens in Figure 1 were performed in biological duplicates because of the large sample sizes and results are shown as "combination" scores derived from casTLE analysis. Proteomics in Figure 3C were performed as technical duplicates which is standard protocol for such a large dataset.<br>For in vivo work, we used n > 4 mice per group, balancing statistical precision with the 3Rs (Replacement, Reduction, Refinement). Based on prior/pilot variability for our endpoints, this sample size was expected to detect large effect sizes (≈ ≥1.5–2.0 SD); smaller effects were outside the scope of this study and would motivate a larger, confirmatory cohort. Mice were divided between males and females and sexes are specified in figure legends and methods. Figure 31,J and Extended Data Figure SA,B were performed as one replicate containing plasma from 4 individual mice or 2 individual mice, respectively, though this is standard protocol since a large amount of plasma is required for this assay.<br>All FIB-SEM data in Figure 6, Extended Data Figure 20, and Extended Data Figure 24 have a sample size of n= l cell because of the high density of data per cell.
Data exclusions	No data were excluded from these analyses.
Replication	All attempts at replication were successful. Figures, including Western blots, flow cytometry, and luminescence/fluorescence/absorbance curves are representative of three biological replicates except for the following, which show single experiments: Figure 3K, Extended Data Figure 4F, and Supplementary Figure 4A. Figure 2G-H and Extended Data Figure 2J-K could only be performed as one replicate but were validated by Figure 2I-J and Extended Data Figure 2L.
Randomization	Randomization was not required because this was a mechanistic in-vitro study using genetically uniform cell lines and predefined perturbations under identical conditions, with rigor ensured by internal controls, replication, and blinded quantification.
Blinding	Because image acquisition and analysis were performed by the same investigator, blinding was not possible; instead we mitigated bias via quantitative metrics, fixed analysis thresholds, and processing all samples in mixed batches under identical settings.

Reporting for specific materials, systems and methods

We require information from authors about some types of materials, experimental systems and methods used in many studies. Here, indicate whether each material, system or method listed is relevant to your study. If you are not sure if a list item applies to your research, read the appropriate section before selecting a response.

Materials & experimental systems

n/a	Involved in the study
☐	☒ Antibodies
☐	☒ Eukaryotic cell lines
☒	☐ Palaeontology and archaeology
☐	☒ Animals and other organisms
☒	☐ Clinical data
☒	☐ Dual use research of concern
☒	☐ Plants

Methods

n/a	Involved in the study
☒	☐ ChIP-seq
☐	☒ Flow cytometry
☒	☐ MRI-based neuroimaging

Antibodies

Antibodies used

CLCC1 (Thermo, HPA009087), PLIN2 (Abcepta, AP5118c, RRID; AB_10662954), albumin (Proteintech, 16475-1-AP, RRID; AB_2242567), MTP (Santa Cruz, sc-515742), CES1/ TGH (R&D Systems, AF4920SP), BiP (Cell Signaling, C50B12), TMEM41B (Proteintech, 29270-1-AP, RRID; AB_2918264), VMP1 (Cell Signaling, D1Y3E, RRID; AB_2714018), lamin A/C (Cell Signaling, 4777), calnexin (Cell Signaling, C5C9), actin (Cell Signaling, 4970, RRID; AB_2223172), GAPDH (Cell Signaling, 2118, RRID; RRID;AB_561053), apoB (Abcam, ab20737, RRID; AB_2056954), apoB (Rockland, AB742, RRID; AB_92217), GM130 (Cell Signaling, 12480, RRID; 2797933), Mab414 (Abcam, ab24609, RRID; AB_448181), IRDye800 conjugated goat anti-rabbit secondary (LI-COR, 926-32211, RRID; AB_2651127), IRDye680 conjugated goat anti-mouse secondary (LI-COR, 926-68070, RRID; AB_2651128), IRDye680 conjugated donkey anti-goat (LI-COR, 926-68074, RRID; AB_2650427), Alexa Fluor 680 conjugated goat anti-mouse secondary (Invitrogen, A21058, RRID; AB-2535724), donkey anti-mouse Alexa Fluor 488 (Thermo Fisher Scientific, A21202, RRID; AB_141607), anti-Rabbit Alexa Fluor 680 (Thermo Fisher, A21109, RRID; AB_2535758), anti-Rabbit Alexa Fluor 488 (Thermo Fisher, A11008, RRID;AB_143165)

Validation

anti-CLCC1, anti-TMEM41B, anti-VMP1, and anti-CES1/TGH were validated using genetic knockout of the endogenous gene with Cas9 and multiple targeted sgRNAs in human cancer cells by the current study. anti-BiP was validated by treating cells with ER stress inducers. All other primary antibodies were used only in manufacturer-validated species and applications; we verified the vendors' validation statements (accessed Oct 2025) and documented RRIDs where possible.

Eukaryotic cell lines

Policy information about cell lines and Sex and Gender in Research

Cell line source(s)

Huh7 cell line was a gift from Dr. Holly Ramage (University of Pennsylvania). HEK293T, U-2 OS, 786-O, and HepG2 cell lines were all obtained from UC Berkeley Cell Culture Facility. LX-2 cell line was from Merck & Co. Primary hepatocytes were collected from male mice.

Authentication

No additional authentication was performed after receipt of cells.

Mycoplasma contamination

All cell lines were negative for mycoplasma.

Commonly misidentified lines
(See ICLAC register)

Cell lines used in the study are not flagged in the Register of Misidentified Cell Lines.

Animals and other research organisms

Policy information about studies involving animals; ARRIVE guidelines recommended for reporting animal research, and Sex and Gender in Research

Laboratory animals

We used C57BL/6J mice (stock no. 00632), male and female, CLCC1 flox/flox from Dr. Greg Ku (UCSF). Mice used in these experiments were housed in the UC Berkeley barrier-free animal Li Ka Shing facility. Mice were housed with a 12 hour light/dark cycle at ambient temperature (23°F) with 30-70% relative humidity. Mice had free access to water and standard chow diet (LabDiet 5053). Experimentation was performed between 8-12 weeks of age.

Wild animals

This study did not involve wild animals

Reporting on sex

Both male and female mice were included in this study to account for potential sex-based differences in metabolic and cellular responses. Weight measurements (liver and body), lipid TLC, albumin, AST, and ALT levels were pooled across sexes as there were no statistical differences between the two. However, we analyzed lipoprotein profiles separately for males and females as there are known sex-based differences in lipid metabolism. Primary hepatocytes could only be isolated successfully from male mice, so images in Figure 5F are from males. FIB-SEM was performed on one male mouse per condition.

Field-collected samples

This study did not involve samples collected from the field.

Ethics oversight

All procedures involving mice were approved by the [Institution Name] IACUC (protocol #[ID], approved [Mon YYYY]) and conducted in accordance with the NIH Guide for the Care and Use of Laboratory Animals, PHS Policy, and the Animal Welfare Act. Note that full information on the approval of the study protocol must also be provided in the manuscript.

Plants

Seed stocks	N/A
Novel plant genotypes	N/A
Authentication	N/A

Flow Cytometry

Plots

Confirm that:

☒ The axis labels state the marker and fluorochrome used (e.g. CD4-FITC).

☒ The axis scales are clearly visible. Include numbers along axes only for bottom left plot of group (a 'group' is an analysis of identical markers).

☒ All plots are contour plots with outliers or pseudocolor plots.

☒ A numerical value for number of cells or percentage (with statistics) is provided.

Methodology

Sample preparation	Cells grown in 6-well plates were washed with PBS, trypsinized and centrifuged for 5 min at 500 x g. Cell pellets were stained with dyes as stated in methods section for 30 minutes and resuspended in PBS before analysis.
Instrument	LSRFortessa (BD Biosciences)
Software	Data was collected using BD FACSDiva v6.2 (BBD Biosciences) and analyzed using FlowJo v10(TreeStar).
Cell population abundance	Cells were not sorted during the procedure.
Gating strategy	Single cells were first gated using the same FSC-H vs SSC-H threshold (P1), and then gated using the same FSC-H vs FSC-A threshold (P2) across all samples. Using P2, FITC, Texas-Red, and Pacific-Blue signals of the gated populations were determined.

☒ Tick this box to confirm that a figure exemplifying the gating strategy is provided in the Supplementary Information.
