# Supplementary material for: CLCC1 promotes hepatic neutral lipid flux and nuclear pore complex assembly
Source: Nature. 2026 Feb 25;652(8109):462–70. doi: 10.1038/s41586-025-10064-4 (PMC13061601; doi:10.1038/s41586-025-10064-4)
Supplement: Supplementary file 6 — Summary of coarse-grained molecular dynamics simulations. A) Self-assembly coarse-grained molecular dynamics started from randomly placed lipids in a simulation box with the protein at the centre. B) Coarse-grained molecular dynamics simulations of proteins embedded in a single DOPC bilayer. C) Series of coarse-grained molecular dynamics simulations of the CLCC1 16-mer between two DOPC bilayers. I–IV correspond to the labels in Extended Data Fig. 9c. Each run was initiated from the final frame of the previous simulation. [file 41586_2025_10064_MOESM6_ESM.docx]

Supplementary Table S4.

|  | **A** | **Residues** | **Copies** | **Pos. restraints** | **# lipids** | **# solvent** | **Box size (Å)** | **Sim. time (μs)** | **T (K)** | **PBC** |
| --- | --- | --- | --- | --- | --- | --- | --- | --- | --- | --- |
|  | Dimer | 56-365 | 2 | Yes | 1 052 | 22 991 | 160x160x160 | 10 | 400 | Yes |
|  | Core | 197-368 | 16 | Yes (0-8 μs)  No (8-12 μs) | 2 500 | 72 113 | 230x230x230 | 12 | 400 | Yes |
|  | Full | 57-365 | 16 | Yes* | 4 750 | 138 276 | 340x340x200 | 2.4 | 400 | Yes |
|  | **B** | **Residues** | **Copies** | **Pos. restraints** | **# lipids** | **# solvent** | **Box size (Å)** | **Sim. time (μs)** | **T (K)** | **PBC** |
|  | Dimer | 56-365 | 2 | No | 1 838 | 63 946 | 253x253x156 | 4 | 310 | Yes |
|  | Full | 57-365 | 16 | No | 4 609 | 227 141 | 406x406x203 | 4 | 310 | Yes |
|  | **C** | **Residues** | **Copies** | **Pos. restraints** | **# lipids** | **# solvent** | **Box size (Å)** | **Sim. time (μs)** | **T (K)** | **PBC** |
| **I** | Full | 57-365 | 16 | Yes* | 8 788 | 227 511 | 411x411x227 | 0.05 | 310 | Yes |
| **II** | Full | 57-365 | 16 | Yes* | 8 788 | 420 780 | 519x519x228 | 0.5 | 310 | No |
| **III** | Full** | 57-365 | 16 | Yes* | 8 788 | 420 780 | 514x514x229 | 0.1 | 310 | No |
| **IV** | - | - | - | - | 5 620 | 114 779 | 302x302x226 | 1 | 310 | Yes |

*No position restraints were applied to the N-terminal domain, residue 57-185.

**Protein-lipid and protein-solvent interactions were switched off linearly
