# Supplementary material for: CLCC1 promotes hepatic neutral lipid flux and nuclear pore complex assembly
Source: Nature. 2026 Feb 25;652(8109):462–70. doi: 10.1038/s41586-025-10064-4 (PMC13061601; doi:10.1038/s41586-025-10064-4)

Figure 2, source data

D-F

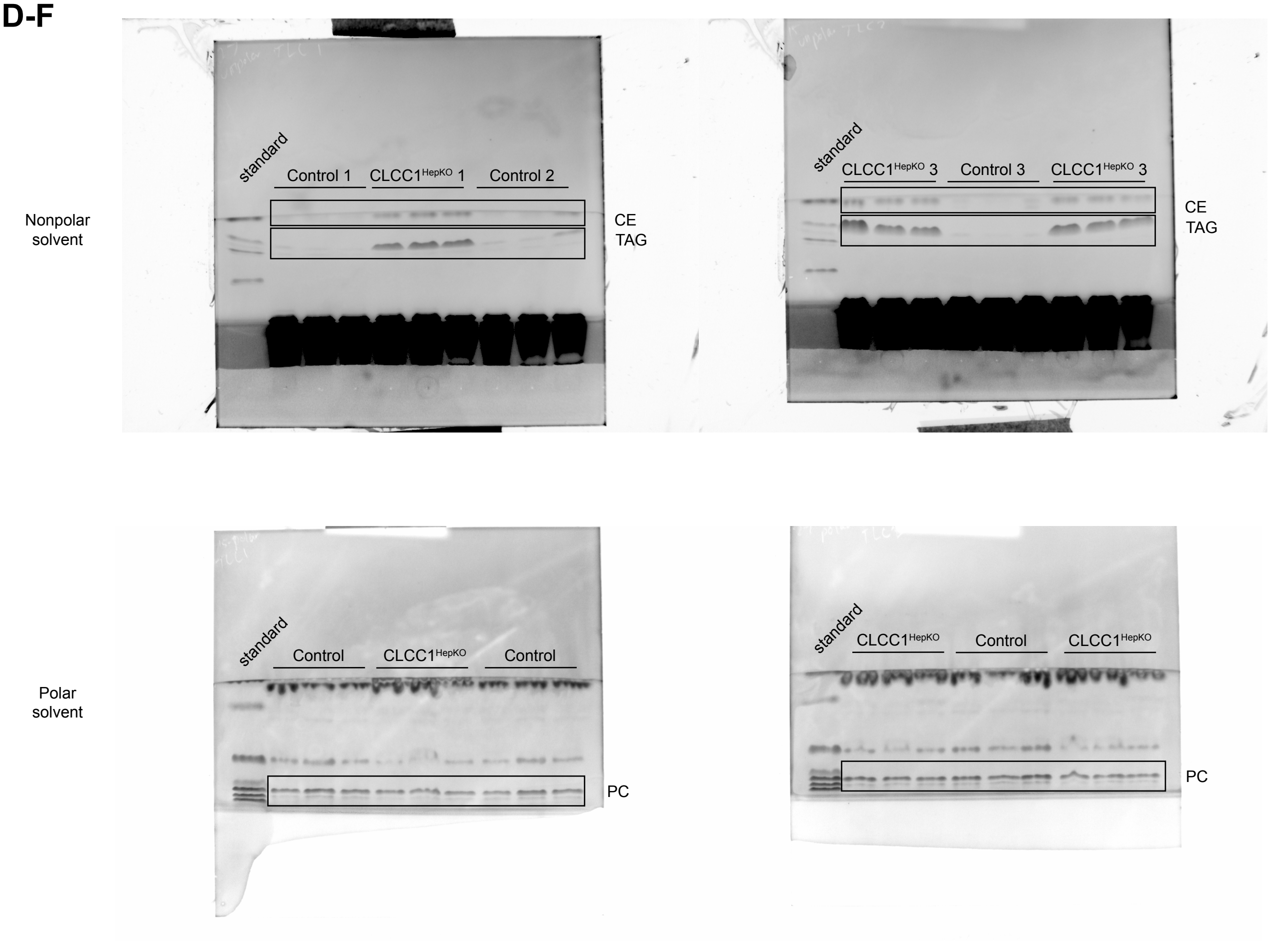

I

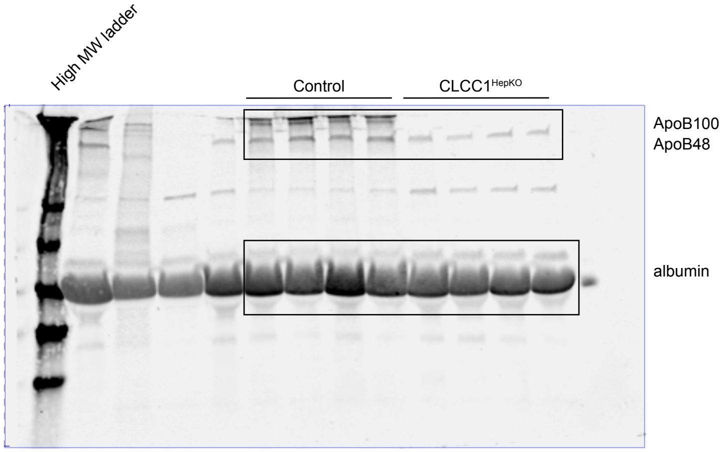

Figure 3, source data

B

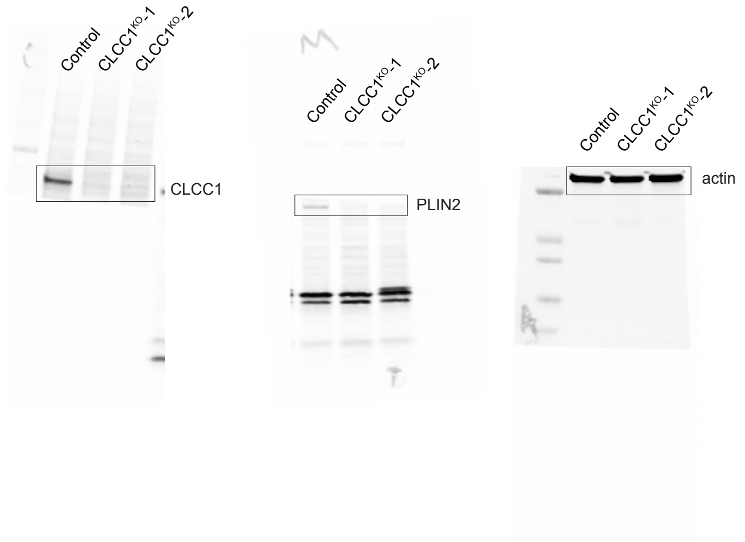

Extended Data Figure 2, source data

G

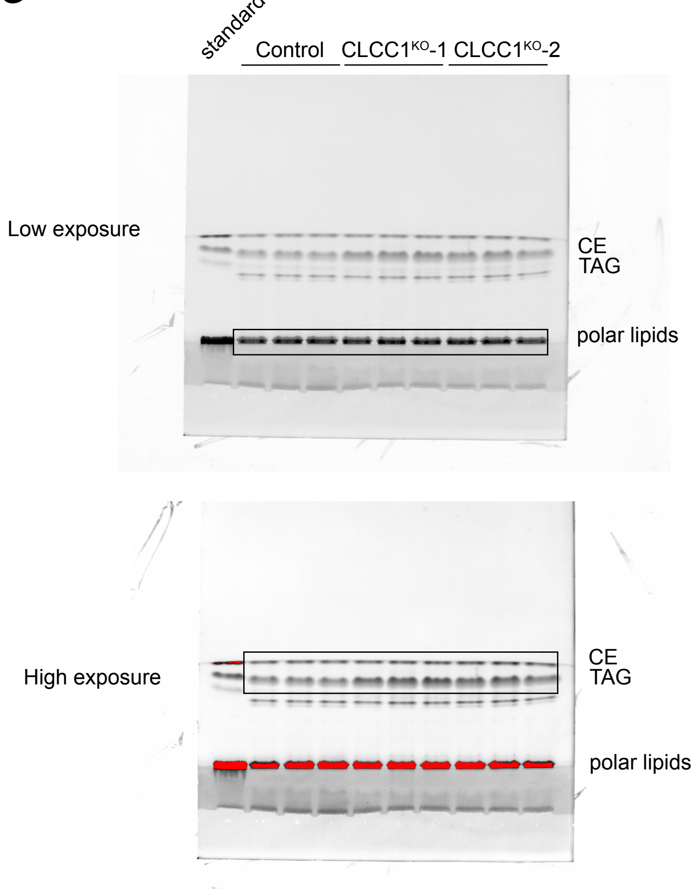

H

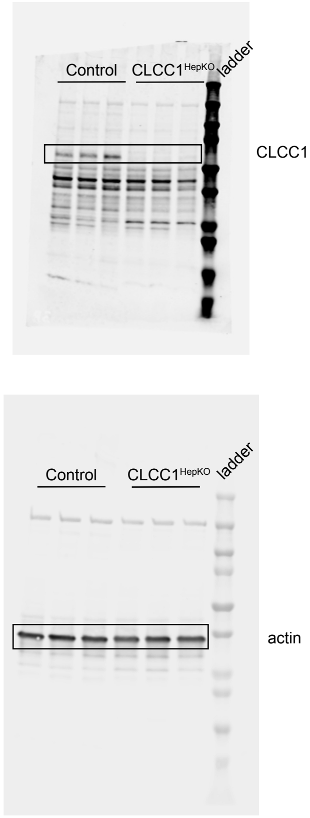

L

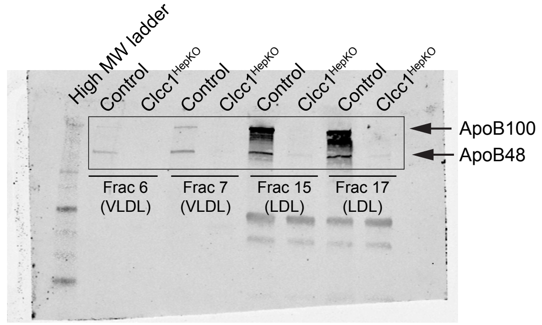

Extended Data Figure 3, source data

A

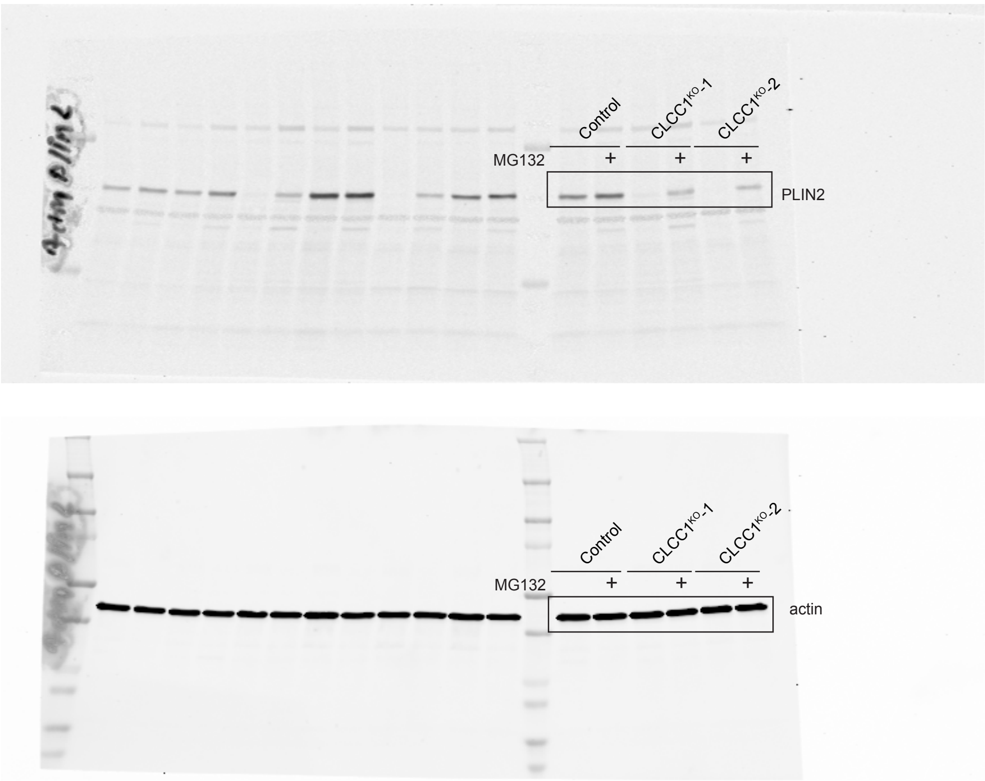

Extended Data Figure 4, source data

F

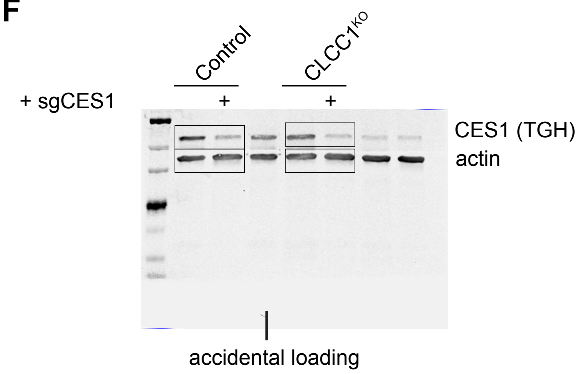

J

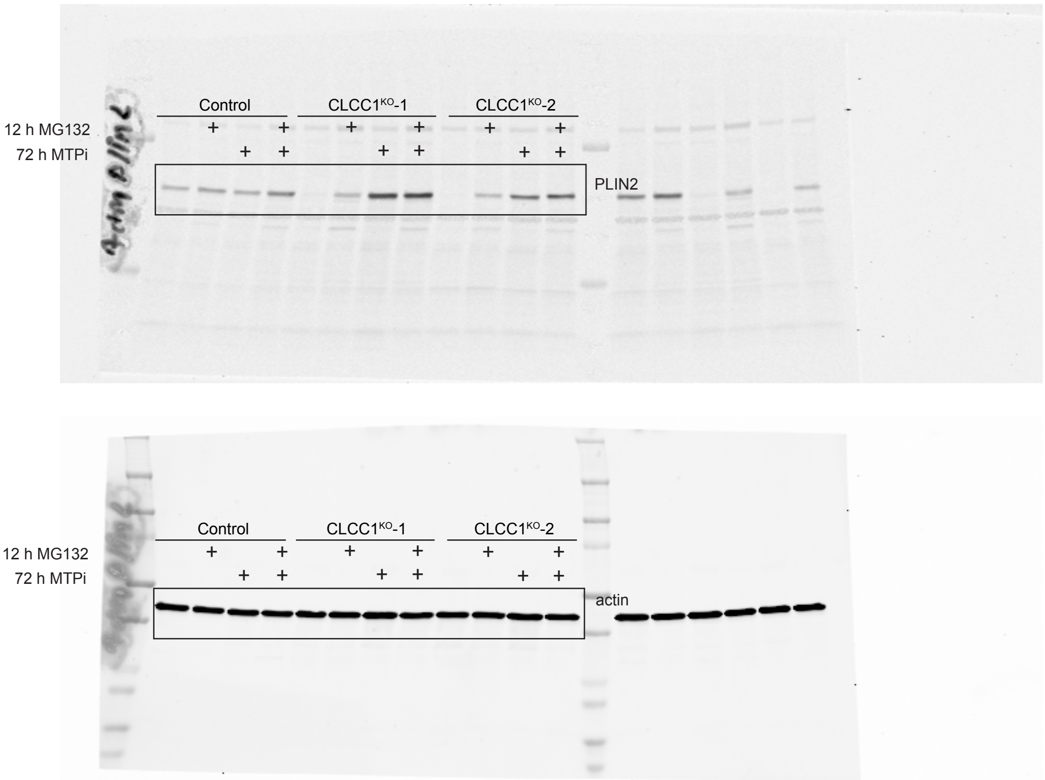

K

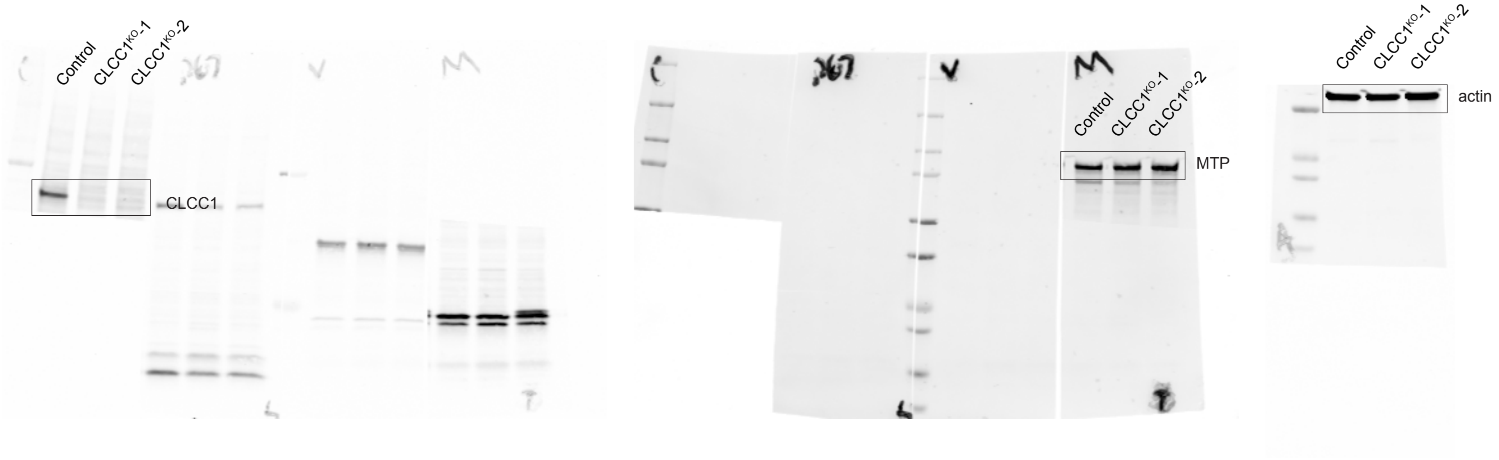

Extended Data Figure 5, source data

in triplicate

B

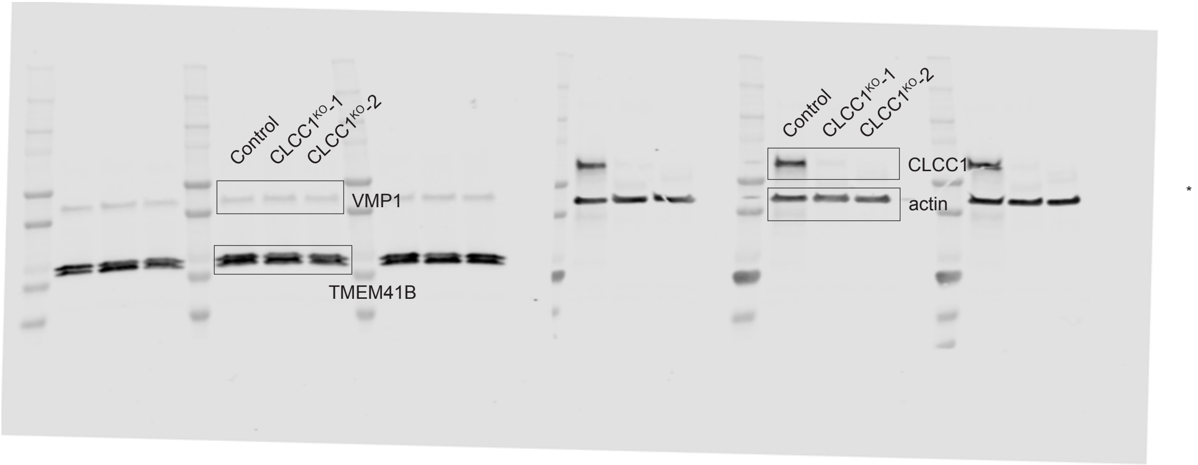

in duplicate

C-D

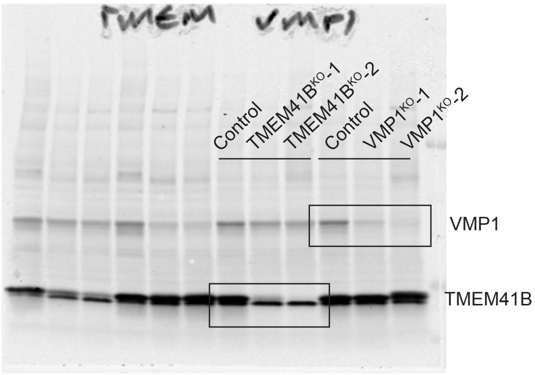

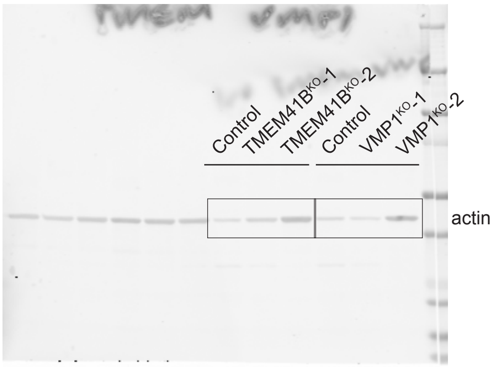

Extended Data Figure 9, source data

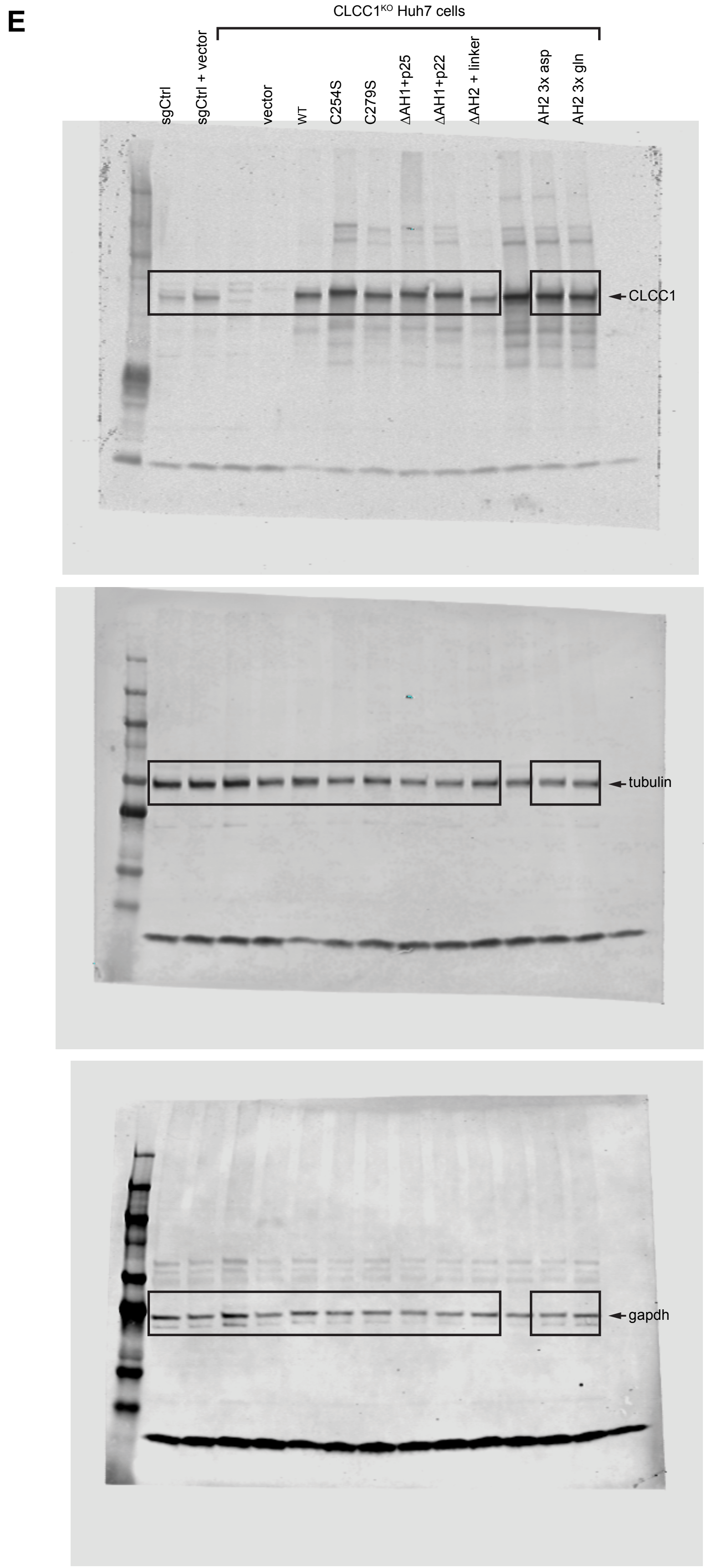

Supplementary Figure 3, source data

A

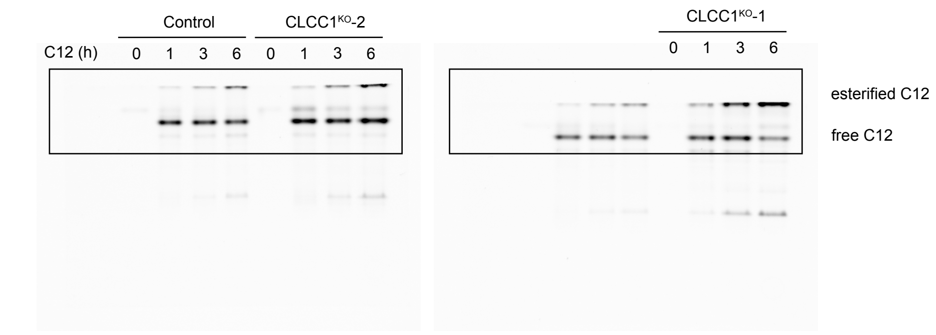

B

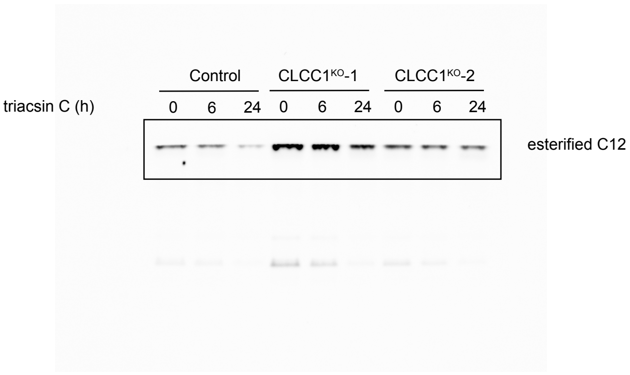

Supplementary Figure 4, source data

A

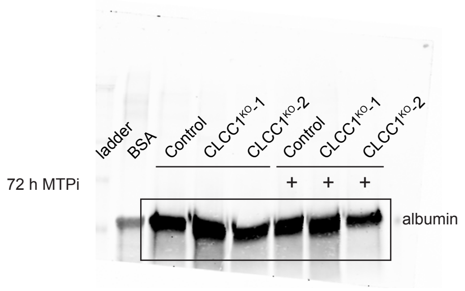

D

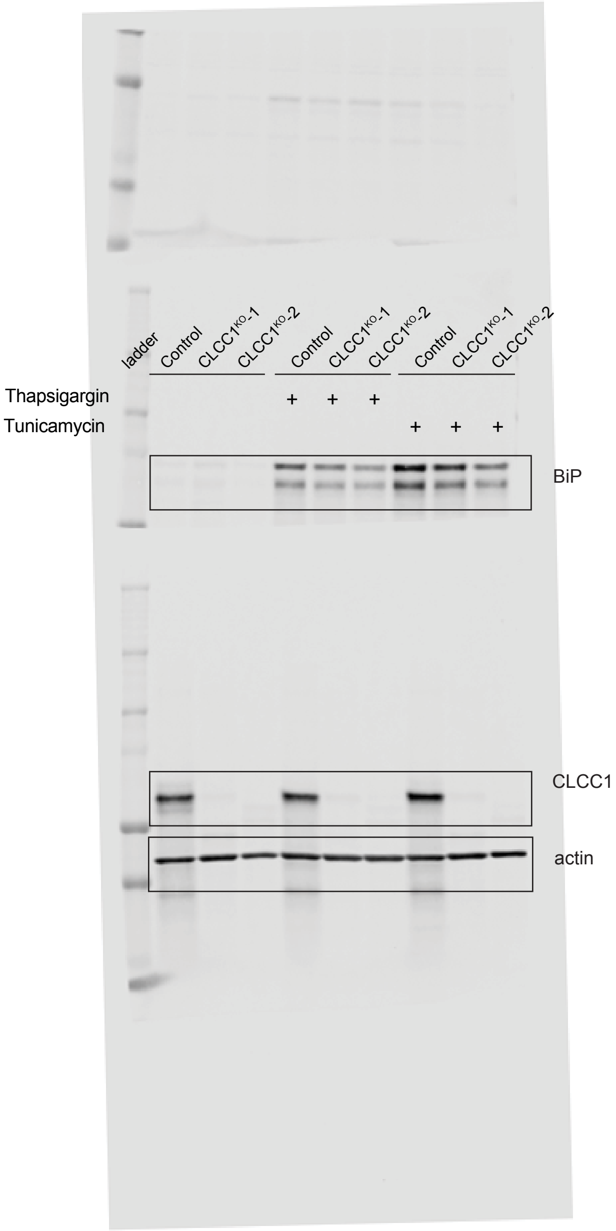

Supplementary Figure 5, source data

A

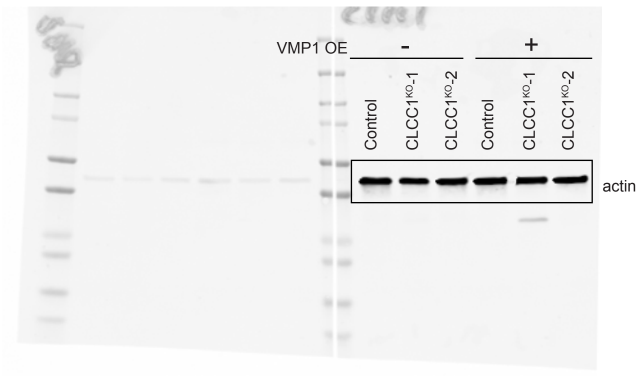

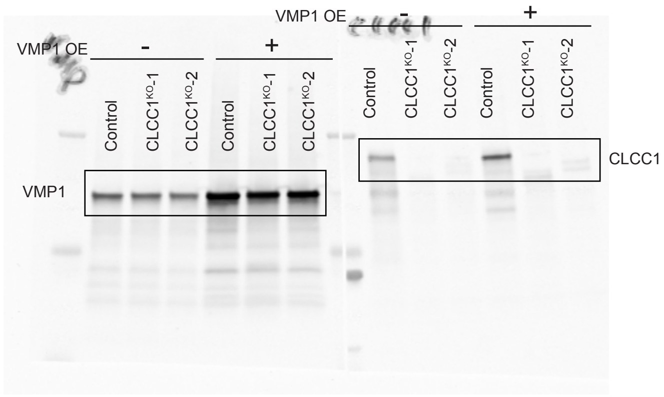

B

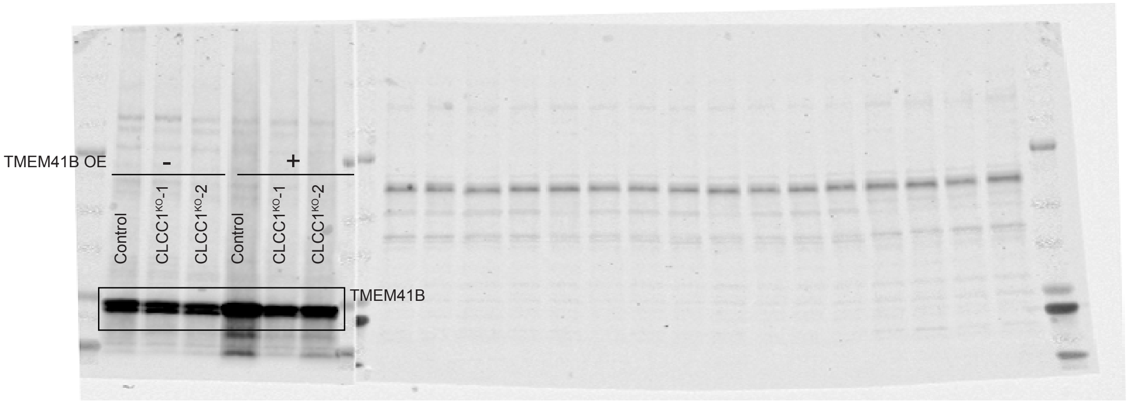

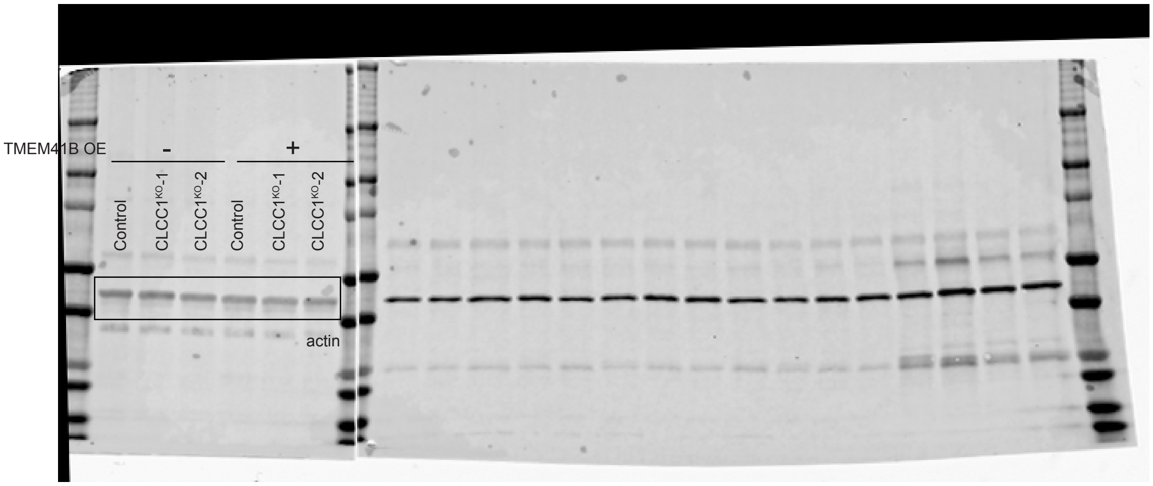

Supplementary Figure 8, source data

C

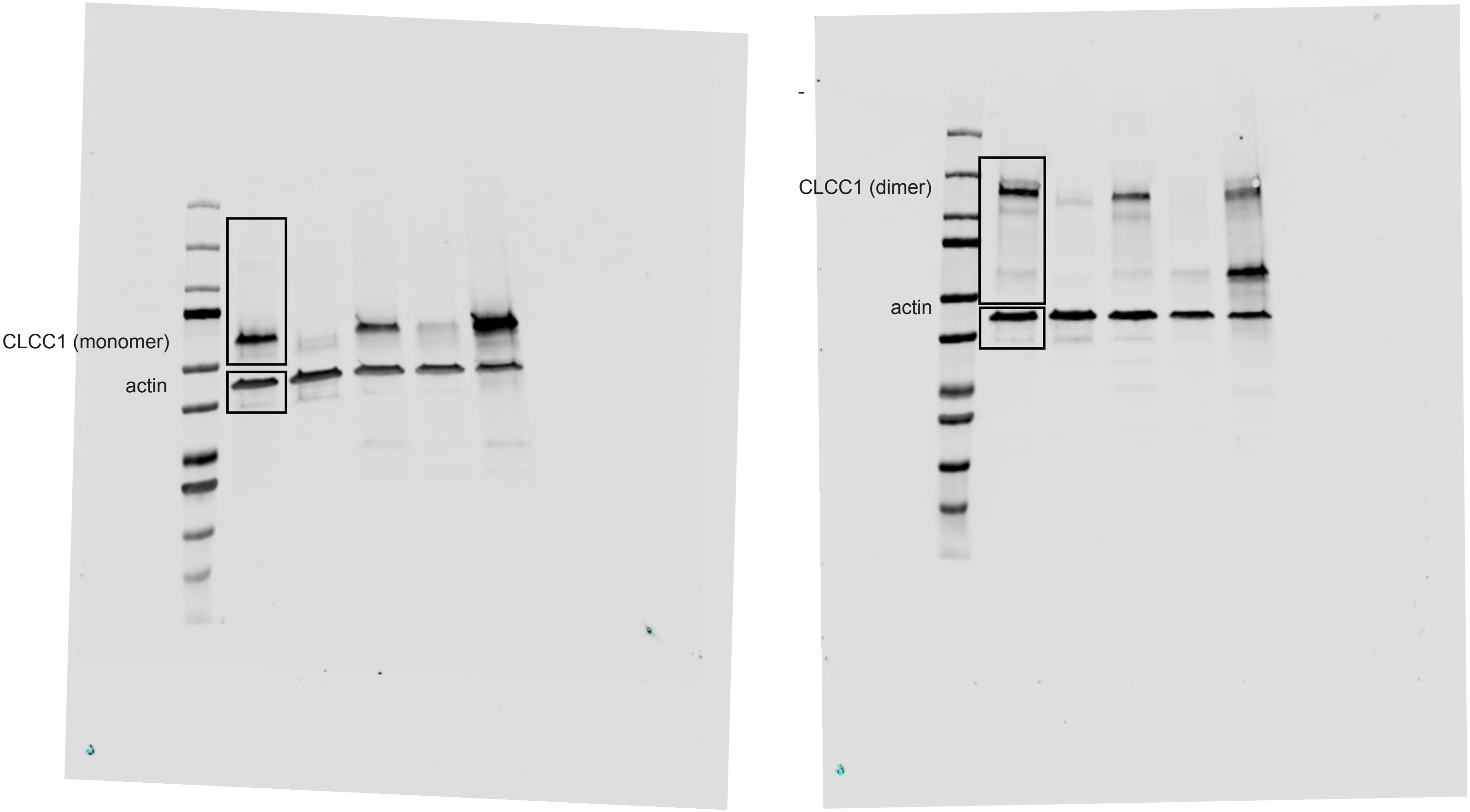

Supplementary Figure 11, source data

A

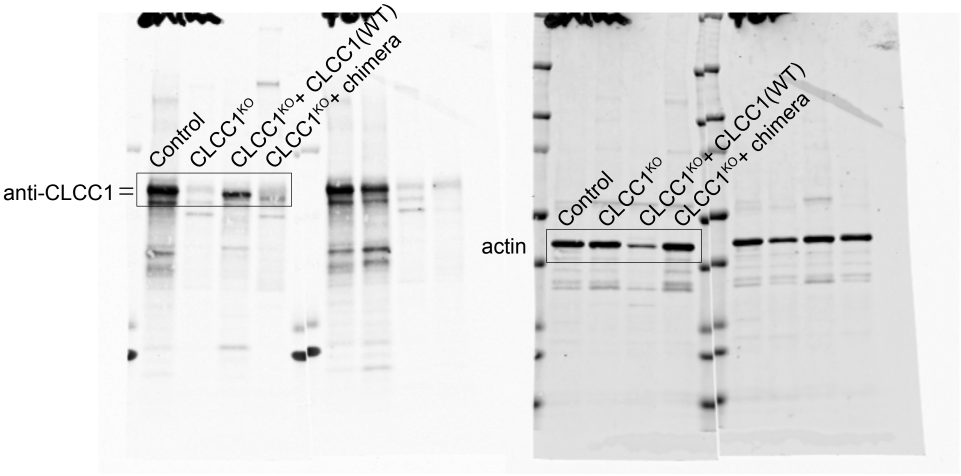

Supplement: Supplementary file 9 — Unprocessed blots for Figs. 2 and 3, Extended Data Figs. 2–5 and 9 and Supplementary Figs. 3–5, 8 and 11. [file 41586_2025_10064_MOESM9_ESM.pdf]
